# Lifecourse genome-wide association study meta-analysis refines the critical life stages for adiposity’s influence on breast cancer risk

**DOI:** 10.1126/sciadv.ady0374

**Published:** 2026-01-21

**Authors:** Grace M. Power, Laxmi Bhatta, Amanda M. Hughes, Carolina Medina-Gomez, Anne Richmond, Genevieve Leyden, Bethan Lloyd-Lewis, Eleanor Sanderson, Rebecca Richmond, Elizabeth C. Corfield, Daniel McCartney, Caroline Hayward, Irene Fontes Marques, Fernando Rivadeneira, Bjørn Olav Åsvold, Gibran Hemani, Janine F. Felix, Ben Brumpton, Alexandra Havdahl, George Davey Smith

**Affiliations:** ^1^ MRC Integrative Epidemiology Unit, University of Bristol, Bristol, UK.; ^2^ Population Health Sciences, Bristol Medical School, University of Bristol, Bristol, UK.; ^3^ Institute for Molecular Bioscience, The University of Queensland, Brisbane, Queensland, Australia.; ^4^ HUNT Center for Molecular and Clinical Epidemiology, Department of Public Health and Nursing, NTNU Norwegian University of Science and Technology, Trondheim, Norway.; ^5^ Division of Mental Health Care, St. Olavs Hospital, Trondheim, Norway.; ^6^ Department of Clinical and Molecular Medicine, NTNU Norwegian University of Science and Technology, Trondheim, Norway.; ^7^ PsychGen Centre for Genetic Epidemiology and Mental Health, Norwegian Institute of Public Health, Oslo Norway.; ^8^ Department of Internal Medicine, Erasmus MC, University Medical Center Rotterdam, Rotterdam, CA 3000, Netherlands.; ^9^ Centre for Genomic and Experimental Medicine, Institute of Genetics and Cancer, The University of Edinburgh, Edinburgh, UK.; ^10^ School of Cellular and Molecular Medicine, University of Bristol, Bristol, UK.; ^11^ Research Department, Lovisenberg Diaconal Hospital, Oslo, Norway.; ^12^ MRC Human Genetics Unit, Institute of Genetics and Cancer, University of Edinburgh, Western General Hospital, Edinburgh, UK.; ^13^ Generation R Study Group, Erasmus MC, University Medical Center Rotterdam, Rotterdam, Netherlands.; ^14^ Department of Pediatrics, Erasmus MC, University Medical Center Rotterdam, Rotterdam, Netherlands.; ^15^ HUNT Research Centre, Department of Public Health and Nursing, NTNU Norwegian University of Science and Technology, Levanger, Norway.; ^16^ Department of Endocrinology, Clinic of Medicine, St. Olavs Hospital, Trondheim University Hospital, Trondheim, Norway.; ^17^ Clinic of Medicine, St. Olavs Hospital, Trondheim University Hospital, Trondheim, Norway.; ^18^ PROMENTA Research Center, Department of Psychology, University of Oslo, Oslo, Norway.; ^19^ NIHR Bristol Biomedical Research Centre Bristol, University Hospitals Bristol and Weston NHS Foundation Trust, University of Bristol, Bristol, UK.

## Abstract

Previous evidence suggests that higher prepubertal adiposity protects against breast cancer risk. Whether this protection extends into early adulthood remains uncertain. We conducted genome-wide association studies on body mass index (BMI) in nulliparous women from menarche to <40 years across five cohorts, with additional analyses in three subintervals of this life stage. Results were meta-analyzed, and two-sample univariable and multivariable Mendelian randomization was applied within a lifecourse framework to assess the effect of BMI on breast cancer risk. Between menarche and <40 years, we observed heterogeneity in genetic effects. Genome-wide correlations further suggest that BMI during this early adult period may be partly influenced by distinct genetic factors compared with adiposity at other life stages. Higher genetically proxied BMI between menarche and 40 years reduced breast cancer risk. This protective effect attenuated after adjusting for prepubertal adiposity. These findings refine our understanding of adiposity’s role in breast cancer and highlight earlier life stages as critical windows for risk modulation.

## Teaser

Improving knowledge of adiposity’s genetic architecture across the lifecourse refines insights into its role in breast cancer.

## INTRODUCTION

Breast cancer is the most commonly diagnosed cancer in women worldwide, with more than 2.3 million new cases and 685,000 deaths reported in 2020 (*1*). Nearly 99% of women diagnosed at the earliest stage survive for 5 years or more, compared to ~32% of those diagnosed at the most advanced stage (*2*). This underscores the importance of improving early detection and highlights the need to identify preventative strategies as key public health objectives.

Breast tissue is particularly vulnerable to exposures between menarche and first full-term pregnancy—a critical window of susceptibility (*3*). After menarche, the breast epithelium undergoes rapid cellular proliferation leading to an increased risk of acquiring mutation during puberty and until the terminal ductal differentiation that accompanies the first full-term pregnancy (*4*, *5*). Evidence suggests that certain exposures, such as physical activity (*3*), may alleviate the risk of breast cancer, while others, such as alcohol consumption (*6*), a known breast carcinogen (*7*), may exacerbate it, by acting during this period of tumorigenic susceptibility. By investigating whether known factors influencing breast cancer risk also exert effects during this timeframe, we may be better able to identify strategies for more effective breast cancer risk detection and potential avenues for prevention. Focusing on this period may additionally enable a more precise assessment by minimizing the confounding effects of physiological changes associated with first birth.

Mendelian randomization (MR) is a technique that exploits the quasi-random distribution of genetic variants from parents to offspring, independent of the influence from other traits. Under specific assumptions, MR aims to estimate causal effects by reducing susceptibility to confounding factors, including confounding by undiagnosed existing disease and disease processes (reverse causation) (*8*, *9*). Recent developments in lifecourse MR methodology include multivariable MR (MVMR) (*10*–*12*). This approach enables the direct estimation of the effects of an exposure measured at a specific life stage, controlling for the same exposure measured at another life stage, on later life outcomes. Previous MVMR analyses suggest that a larger prepubertal body size, used as a proxy for body mass index (BMI), is protective against breast cancer (*13*–*16*). This protective effect persists even after controlling for later life adult body size, which has a negligible effect on breast cancer risk when prepubertal body size is controlled for. These findings align with conventional analyses (*17*–*23*) and have been further supported through a proxy-genotype MR approach, where offspring genotype proxies parental genotype to assess associations with parental disease risk (*24*). It has been proposed that such findings may be influenced by selection bias, as both childhood adiposity and breast cancer status could affect the likelihood of surviving to or participating in large-scale cohort studies (*25*). However, simulations examining the relationship between childhood body size and subsequent breast cancer risk indicate that, although bias can arise—particularly under interaction-driven selection (*26*)—the magnitude remains insufficient to account for the protective effects observed in MR analyses (*27*).

While a larger body size in childhood has been shown to reduce breast cancer risk, it also leads to earlier menarche, a known risk factor for breast cancer (*13*). This dual effect suggests that the protective direct effect of prepubertal body size on breast cancer could be larger than its total observed effect, as it may be partially attenuated by an increased risk mediated through earlier menarche. This interplay highlights two potentially independent pathways that may interact in complex ways. Despite convincing evidence for the protective effects of prepubertal body size on breast cancer risk, the time period at which this protective effect diminishes is yet to be determined using causal inference methods.

We aimed to estimate the effect of higher BMI between menarche and first full-term pregnancy—or throughout early adulthood (<40 years) in individuals who remain nulliparous—on breast cancer risk later in life. This previously understudied period represents a critical window of susceptibility to exposures influencing breast cancer risk. We first conducted genome-wide association studies (GWAS) of BMI in nulliparous women from five population-based cohorts across the full period from menarche to <40 years and then stratified the analyses into three groups: menarche to <20 years, 20 to <30 years, and 30 to <40 years. This approach allowed us to assess the consistency of genetic variant effects on BMI across early adulthood and to apply these profiles in downstream lifecourse MR analyses. We then assessed these effects on overall breast cancer and different subtypes, including ER status (ER^+^/ER^−^) and five molecular subtypes: luminal A–like, luminal B–like (HER2-positive), luminal B (HER2-negative-like), HER2-enriched, and triple-negative breast cancer using data from the Breast Cancer Association Consortium (BCAC) (*28*, *29*). While we were not able to assess effects by menopausal status directly, we examined subtype-specific effects as a means of providing preliminary insights given that some evidence indicates that certain subtypes may be more common before verses after menopause (*30*–*32*).

## RESULTS

Measurements of BMI between menarche and age < 40 years were available for 56,863 nulliparous women across five cohorts: the Avon Longitudinal Study of Parents and Children (ALSPAC), the Trøndelag Health Study (HUNT), the Norwegian Mother, Father and Child Cohort Study (MoBa), Generation R, and Generation Scotland. Corresponding phenotype sample sizes and cohort characteristics for age and BMI are presented in Table 1. Within the primary <40-year group, mean ages at measurement ranged from 23.9 years in ALSPAC to 29.4 years in Generation R, while mean BMI values varied between 22.8 kg/m^2^ in ALSPAC and 24.5 kg/m^2^ in Generation Scotland. To explore patterns across narrower age intervals within this broader period, we also conducted analyses stratified into three groups: menarche to <20 years, 20 to <30 years, and 30 to <40 years. Phenotypic sample sizes and summary measures of age and BMI for the full <40 group and each stratum are shown in Table 1.

**Table 1.** Descriptive statistics for nulliparous women across defined life stages. Descriptive statistics for nulliparous women between menarche and <40 years, menarche and <20 years, 20 and <30 years, and 30 and <40 years across all cohorts. ALSPAC, the Avon Longitudinal Study of Parents and Children; HUNT, the Trøndelag Health Study; MoBa, the Norwegian Mother, Father and Child Cohort Study; BMI, body mass index; N, number of nulliparous women with the trait measured; SD, standard deviation; min, minimum measurement; max, maximum measurement.

			Age (years)		BMI (kg/m^2^)	
	Cohort	*N*	Mean (SD)	Median (min-max)	Mean (SD)	Median (min-max)
ALL (MENARCHE TO <40 YEARS)	ALSPAC	5,047	23.92 (6.01)	24.00 (14.00–39.00)	22.79 (3.86)	21.98 (12.50–51.16)
	HUNT	7,683	24.40 (5.04)	23.7 (12.80–39.90)	23.40 (3.99)	22.50 (15.34–52.20)
	MoBa	38,687	25.75 (6.68)	27.00 (14.00–39.00)	23.16 (4.01)	22.38 (8.11–46.07)
	Generation R	2,396	29.44 (4.71)	30.13 (15.96–39.95)	23.07 (4.04)	22.09 (14.53–49.82)
	Generation Scotland	3,059	26.29 (5.95)	26.00 (14.00–39.00)	24.52 (5.28)	23.24 (13.13–57.94)
MENARCHE TO <20 YEARS	ALSPAC	2,047	17.78 (0.42)	17.75 (14.00–19.33)	22.79 (4.10)	21.92 (13.11–50.06)
	HUNT	1,093	16.90 (2.01)	17.00 (12.80–19.90)	21.95 (3.07)	21.48 (15.34–46.87)
	MoBa	7,834	14.74 (1.22)	14.00 (14.00–19.00)	20.91 (3.06)	20.45 (8.92–40.75)
	Generation R					
	Generation Scotland	430	18.15 (0.94)	18.00 (14.00–19.00)	22.88 (3.96)	22.05 (15.5–43.09)
20 TO <30 YEARS	ALSPAC	3,520	24.91 (2.12)	24.00 (20.00–29.00)	23.61 (4.44)	22.52 (14.22–57.88)
	HUNT	5,507	24.18 (2.69)	23.70 (20.00–29.90)	23.50 (3.99)	22.7 (15.60–52.20)
	MoBa	18,578	25.86 (2.51)	26.00 (20.00–29.00)	23.66 (4.05)	22.78 (8.11–45.31)
	Generation R	1,127	26.08 (2.86)	26.62 (20.01–29.99)	23.24 (4.47)	22.20 (14.53–49.82)
	Generation Scotland	1,745	24.41 (2.81)	24.00 (20.00–29.00)	24.31 (5.03)	23.12 (13.13–54.95)
30 TO <40 YEARS	ALSPAC	1,054	32.65 (2.45)	32.00 (30.00–39.00)	22.72 (3.65)	21.97 (12.50–47.30)
	HUNT	1,083	33.09 (2.60)	32.30 (30.00–39.90)	24.32 (4.45)	23.30 (17.00–48.50)
	MoBa	12,275	32.61 (2.38)	32.00 (30.00–39.00)	23.84 (3.97)	22.99 (8.50–46.07)
	Generation R	1,269	33.12 (2.25)	32.76 (30.00–39.95)	22.98 (3.67)	22.06 (16.90–43.34)
	Generation Scotland	884	33.98 (2.85)	34.00 (30.00–39.00)	25.72 (6.00)	24.05 (13.7–57.94)

### Life stage–stratified GWAS and meta-analysis

**Fig. 1.**
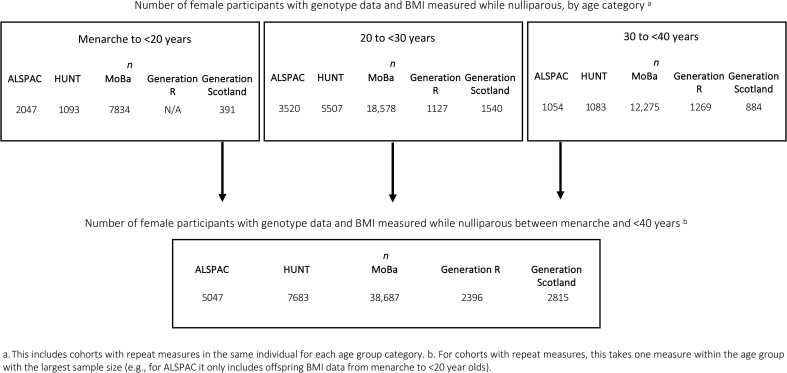
Overview of study participants. Overview of study participants used for this analysis. ALSPAC, the Avon Longitudinal Study of Parents and Children; HUNT, the Trøndelag Health Study; MoBa, the Norwegian Mother, Father and Child Cohort Study; BMI, body mass index; N, number of nulliparous women with the trait measured; N/A, not applicable.

Genome-wide association analyses (GWAS) for BMI were conducted in up to 56,628 nulliparous women with measures between menarche and age < 40 years, and results were meta-analyzed across cohorts. Stratified meta-analyses for the three life stages—menarche to <20 years, 20 to <30 years, and 30 to <40 years—included 11,365, 30,272, and 16,565, respectively (Fig. 1). For cohorts with repeat measures within one life stage (e.g., menarche to <40 years), we retained the time point with the largest sample size (e.g., for ALSPAC, only BMI data from participants <20 years were included).

Overall, 31, 3, 12, and 5 lead genetic variants were identified as associated with BMI in nulliparous women between menarche to <40 years, menarche to <20 years, 20 to <30 years, and 30 to <40 years before giving birth, respectively, based on conventional genome-wide significance (*P* < 5 × 10^−8^) and linkage disequilibrium (LD) clumping within each life stage to identify conditionally independent signals (table S1). 

In total, 45 unique single-nucleotide polymorphisms (SNPs) were identified across all groups. Nine loci reached genome-wide significance in every life-stage GWAS (menarche to <40 years, <20 years, 20 to <30 years, and 30 to <40 years). Additional overlap was observed between the combined menarche to <40 years analysis and the stratified groups, with 9 loci genome-wide significant in both the combined and <20 years groups, 26 in both the combined and 20 to <30 years groups, and 13 in both the combined and 30 to <40 years groups. Although such overlap is expected given the nested design, these findings indicate that, while some loci were detected only within certain intervals, a core set of loci remained consistently associated with BMI across adolescence and early adulthood.

Because clumping was conducted independently for each time window, some loci are represented by different lead SNPs across groups, reflecting tagging of the same underlying association signal. In addition, because the narrower age windows are nested within the broader menarche to <40 years group, many SNPs identified in the subgroups, or SNPs in high LD with them, also appear in the full-period analysis. To assess redundancy and overlap across stages, we applied LD clumping to the compilation of all 45 SNPs retained after stage-specific clumping. This revealed that they correspond to 31 independent genomic regions, indicating that several loci are shared across groups despite being tagged by different SNPs. Most of these independent signals were identified within the menarche to <40 group, which is expected given its larger sample size and broader coverage. SNPs that remained in the final set of 31 independent regions are marked in table S1.

Cochran’s *Q* statistic was used to evaluate heterogeneity in SNP-exposure associations across the five cohort studies included in the primary analysis. To account for multiple testing across the 31 SNPs, a Bonferroni correction was applied. After adjustment, none of the variants showed evidence of heterogeneity (*Q*_bonf_ < 0.05), suggesting consistent effect estimates across studies. This reinforces their validity in downstream MR analyses, reducing concerns that study-level differences may confound associations with the outcome. Full heterogeneity results, including uncorrected *P* values and *Q* statistics, are provided in table S2.

**Fig. 2.**
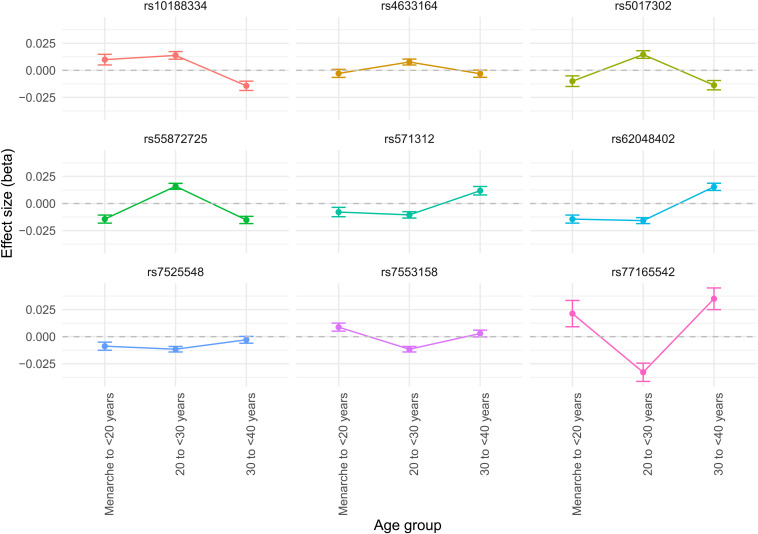
Age-specific SNP effect trajectories on BMI. Plot illustrating the age-specific effect trajectories of individual SNPs with significant heterogeneity (*Q*FDR < 0.05) on BMI across three defined life stages: menarche to <20 years, 20 to <30 years, and 30 to <40 years, in nulliparous women. Each plot represents a SNP exhibiting significant heterogeneity (*Q*FDR < 0.05), illustrating its effect size (beta) across life stages. Error bars denote 95% confidence intervals.

We visualized the life stage–specific effect trajectories of these SNPs at each life stage (menarche to <20 years, 20 to <30 years, and 30 to <40 years), with point size inversely proportional to the SE of the effect estimate (fig. S1). This revealed two notable patterns of heterogeneity: (i) SNP effects in the 20- to <30-year life stage differ markedly from those in the menarche to <20-year and 30- to <40-year life stages, and (ii) some SNPs show a reversal in effect after age 30. Among 45 independent variants, nine showed substantial heterogeneity across the three life stages (Cochrane's Q with false discovery rate correction; *Q*FDR < 0.05; Fig. 2), with the *Q*FDR serving as a heuristic metric to quantify age-dependent heterogeneity. These distinct patterns underscore age-specific heterogeneity that would likely be overlooked by linear meta-regression, highlighting the complexity of SNP effects on BMI over time.

### Heritability estimation, LD score regression, and genetic correlation analyses

We quantified the proportion of variance of the four meta-analyses undertaken on BMI in nulliparous women at different ages of European ancestry that can be explained by our SNP sets using linkage disequilibrium score regression (LDSC) (*33*). The GWAS summary statistic SNP-based heritability ranged from 0.11 (SE = 0.01) for BMI in nulliparous women between 30 and <40 year olds to 0.23 (SE = 0.02) for BMI in nulliparous women between 20 to <30 year olds (Table 2). These results indicate that genetic factors play an important role in BMI, particularly in the younger life stages. The number of genome-wide significant loci is broadly in line with expectations, increasing with greater statistical power, driven by higher heritability ($h^{2}$) and greater sample size, supporting a polygenic architecture across life stages.

**Table 2.** Summary of meta-analyzed genome-wide association results by life stage BMI categories. Summary metrics from meta-analyzed genome-wide association results for BMI in nulliparous women across four life-stage BMI categories: between menarche and <40 years, menarche and <20 years, 20 and <30 years, and 30 and <40 years. BMI, body mass index; SE, standard error; h², heritability (observed scale); LDSC, LD score regression.

	Age group category			
Metric	BMI in nulliparous women between menarche and <40 years	BMI in nulliparous women between menarche and <20 years	BMI in nulliparous women between 20 and <30 years	BMI in nulliparous women between 30 and <40 years
	Estimate (SE)	Estimate (SE)	Estimate (SE)	Estimate (SE)
Heritability (observed scale h^2^)	0.19 (0.01)	0.21 (0.03)	0.23 (0.02)	0.11 (0.01)
Genomic inflation factor (lambda GC)	1.27	1.07	1.17	1.09
LDSC intercept	1.01 (0.01)	1.01 (0.01)	1.00 (0.01)	1.01 (0.01)
Proportion of inflation (ratio)	0.03 (0.02)	0.13 (0.08)	0.01 (0.04)	0.07 (0.06)

**Fig. 3.**
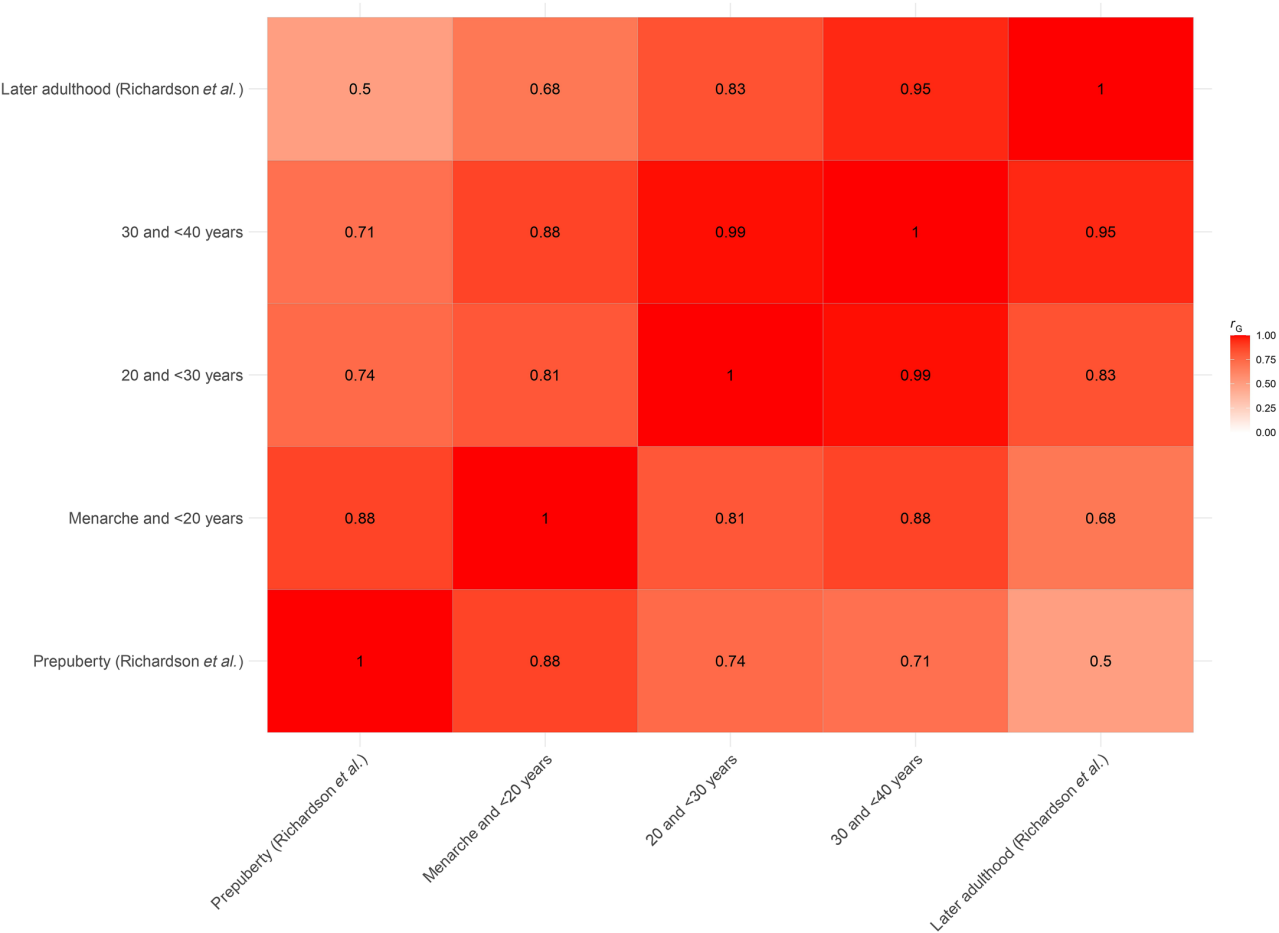
Genetic correlation heatmap for BMI and body size across life stages. Genetic correlation heatmap for BMI and body size (used as a proxy for BMI) across various life stages in females, spanning from prepuberty through menarche and first birth to later adulthood.

The genetic correlations (*r*_G_) observed between prepubertal body size (used as a proxy for BMI) in females and BMI at different stages in nulliparous women from menarche to <40 years reveal consistently strong associations, as expected when investigating a similar phenotype (BMI and body size) across different life stages. The *r*_G_ of 0.76 (SE = 0.02, *P* = 2.13 × 10^−223^) between prepubertal body size in females and BMI in nulliparous women <40 years indicates a high degree of shared genetic influence (table S3). This trend was mirrored at more granular age intervals, with *r*_G_ values of 0.88, 0.74, and 0.71 for BMI in nulliparous women from menarche to <20, 20 to <30, and 30 to <40 years, respectively (Fig. 3). These results suggest that prepubertal body size has a slightly stronger genetic correlation in younger life stages, particularly for BMI measured before age 20. Similarly, body size (used as a proxy for BMI) in later life adult women shows strong genetic overlap with BMI measured in nulliparous women at various age periods, with *r*_G_ values ranging from 0.68 (for BMI in nulliparous women from menarche to <20 years) to 0.95 (for BMI in nulliparous women aged 30 to <40 years). The consistently high *r*_G_ values across all comparisons, particularly the near-perfect correlation between body size measured in later life adult women and BMI in nulliparous women aged 30 to <40 (*r*_G_ = 0.95, SE = 0.05), may reflect an enduring genetic influence of BMI from adolescence into later reproductive years (Fig. 3 and table S3). The genetic correlation is unaffected by differences in measurement scale, as it reflects shared genetic architecture rather than observed variance. One *r*_G_ estimate calculated using LDSC slightly exceeded 1, which is a known feature of the method due to sampling variability and its unconstrained estimation framework. This value is not represented in the figure but is included in table S3, where all *r*_G_ values are reported. While overlapping samples can contribute to inflation, LDSC intercepts adjust for this, and such values are not necessarily indicative of bias or methodological error.

### Lifecourse Mendelian randomization analysis

**Fig. 4.**
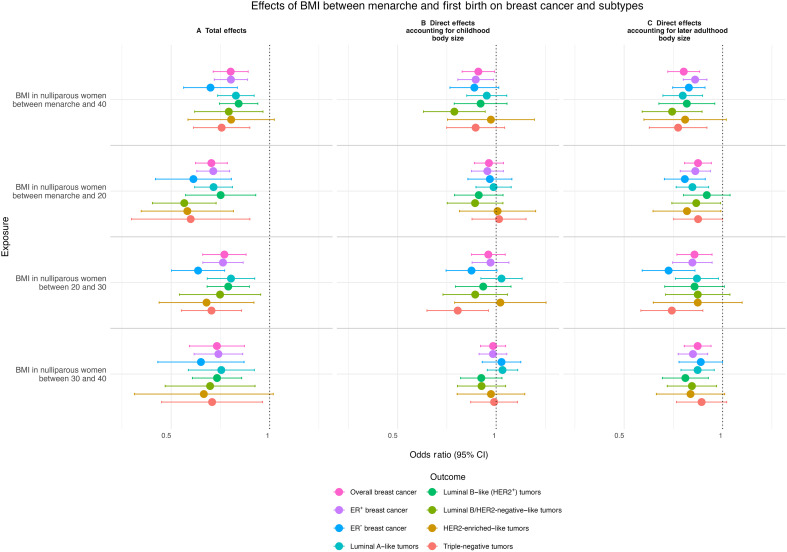
Univariable and multivariable MR estimates of BMI effects on breast cancer. Univariable and multivariable MR analyses for BMI between menarche and first birth on breast cancer (overall and subtype samples). (**A**) The total effects (univariable MR). (**B**) The direct effect accounting for childhood body size. (**C**) The direct effect accounting for later-life adulthood body size (multivariable MR). The plots present the odds ratio of breast cancer per SD increase in natural log-transformed BMI. Error bars indicate 95% confidence intervals (CI) around the point estimates from IVW univariable MR and IVW multivariable MR analyses. BMI, body mass index.

Univariable MR analyses indicated evidence that a one SD increase in genetically predicted log-transformed BMI in nulliparous women between menarche and <40 years reduced the risk of overall breast cancer [inverse variance weighted (IVW) odds ratio, 95% confidence interval: 0.76, 0.67 to 0.86, *P* = 1.27 × 10^−5^] (Fig. 4 and table S4). Similar inverse effects were observed across most breast cancer subtypes, except for the HER2-enriched subtype, which showed little evidence of protection. In MVMR accounting for later life adult body size, higher genetically predicted BMI in this window continued to provide evidence of a reduced risk of overall breast cancer and most subtypes, with HER2-enriched disease remaining the exception. Adjustment for prepubertal body size led to a marked, although not complete, attenuation of these effects, consistent with part of the protection being shared with childhood adiposity. Some evidence of a protective effect was observed for luminal B–like (HER2^+^) tumors (Fig. 4 and table S4).

In the narrower life-stage periods obtained by partitioning the broader fertile-window measure, univariable MR analyses indicated evidence that higher genetically predicted BMI in nulliparous women between menarche to <20, 20 to <30, and 30 to <40 years reduced the risk of overall breast cancer and most subtypes, although the strength and precision of effects varied by subtype and life-stage period. After accounting for later life adult body size, protective effects were largely retained, with most estimates still supporting a reduction in risk. In contrast, adjusting for prepubertal body size removed much of the apparent protection in these narrower periods. For triple-negative disease, effect estimates suggested modest residual protection in the 20 to <30 window after accounting for childhood body size. Weighted median estimates were consistent in direction and magnitude. MR-Egger analyses provided little evidence of horizontal pleiotropy for the combined menarche to <40 exposure; for the narrower life-stage windows, estimates were imprecise due to limited statistical power (table S4). To further assess the validity of the inferred causal direction, we applied Steiger filtering, which tests whether the genetic instruments explain more variance in the exposure than in the outcome. Across all life stages, the proportion of variance explained (*R*^2^) was consistently higher for BMI than for breast cancer, supporting the hypothesized direction of effect from BMI to breast cancer (table S5).

Using Mendelian randomization robust adjusted profile score (MR-RAPS) with an instrument selection threshold of *P* < 1 × 10^−5^, genetically predicted BMI between menarche and <40 years, as well as between 20 and <30 years, showed strong evidence of a protective effect on overall breast cancer risk. Weaker evidence was observed for BMI between 30 and <40 years, while very little evidence of an effect was found for BMI between menarche and <20 years (table S6). MVMR results should be interpreted with caution since conditional *F*-statistic was <10 for each of the BMI exposures included in these models, indicating potential for weak instrument bias (table S4).

### Sensitivity analyses

To minimize pregnancy-related confounding, we restricted the BMI GWAS to nulliparous women, which enabled a clearer assessment of BMI’s direct influence across life stages. However, this restriction may introduce selection (collider) bias (*34*) if genetically proxied parity is associated with BMI within the nulliparous sample, as parity was not conditioned on in the breast cancer outcome GWAS. Our analyses indicated very little evidence that genetically proxied parity associated with BMI in nulliparous women between menarche and <40 years, menarche and <20 years, and 20 and <30 years. There was some evidence that genetically proxied parity associated with lower BMI in nulliparous women between 30 and <40 years (table S7). There was very little evidence of effect in the reverse direction (BMI-associated variants on parity). These results suggest collider bias due to selecting nulliparous women is likely to be of little concern within this study.

After adjusting for age at menarche, the estimated effect of BMI in nulliparous women between menarche and <40 years on overall breast cancer remains largely consistent with the UVMR analyses (table S8). Age at menarche itself showed a little direct effect on overall breast cancer or most subtypes once BMI was accounted for.

## DISCUSSION

In this study, we undertook GWAS and MR analyses within a lifecourse framework to (i) assess the consistency of the genetic effects of BMI across different life stages and (ii) investigate the effects of genetically proxied BMI in nulliparous women between menarche and <40 years on the risk of overall breast cancer and seven subtypes. This work builds on previous research to further the investigation into the influence of BMI during critical periods on breast cancer risk (*13*, *14*, *35*). Motivated by recent findings suggesting that a larger prepubertal body size, used as a proxy for BMI, may offer protection against breast cancer risk, while later life body size shows very little effect, we aimed to better understand the interval between puberty and later life. Specifically, we focused on the period between menarche and first full-term pregnancy, a crucial window of vulnerability for later life breast cancer development.

We observed variation in genetic effects on BMI across different life stages for nine of the 45 discovery variants identified in this study. Some of this variation may reflect statistical fluctuation introduced by selecting lead SNPs based on age-stratified significance thresholds. However, several variants showed clear changes in the direction of effect across the life stage groups. While some directional shifts may reflect genuine developmental effects, we cannot conclusively distinguish these from statistical artifacts that may arise from mechanisms such as the winner’s curse or age-related selection bias, which could exaggerate apparent fluctuations in effect size. In addition, genome-wide cross–life stage genetic correlations demonstrated variation, e.g., when comparing prepubertal body size and later life body size with BMI from menarche to <40 years.

In the univariable MR analyses, higher BMI in nulliparous women from menarche to <40 years was found to consistently reduce risk of overall breast cancer and most subtypes. Similar protective effects were observed when BMI was measured within narrower periods of this window (menarche to <20, 20 to <30, and 30 to <40 years). These protective effects largely remained after accounting for later life adult body size. In contrast, adjusting for prepubertal body size in MVMR led to greater attenuation, indicating that part of the protective effect of BMI in early reproductive life may be explained by body size before puberty. The attenuation was more pronounced for the narrower life-stage periods than for the full fertile-window measure, reflecting both biological and methodological influences. Biologically, part of the apparent effect across all reproductive-age windows overlaps with childhood adiposity, so adjustment for prepubertal body size reduces the estimates. Methodologically, the narrower age-specific GWASs have substantially smaller sample sizes than the prepubertal childhood GWAS generated using UK Biobank data. In MVMR, this imbalance can magnify the degree of attenuation by limiting power to detect effects independent of prepubertal body size. By contrast, the broader reproductive-age GWAS generated in this study is derived using a larger sample and therefore shows less attenuation, despite adjustment for the same childhood body size data. No such attenuation was observed when adjusting for later-life body size, although similar sample size imbalances exist. This indicates that differential power alone may not fully explain the observed differences. Future methodological work is needed to examine how sample size disparities across life-stage GWASs influence estimates in MVMR and to develop strategies for accounting for this in lifecourse applications.

Our findings are in line with previous MR and conventional epidemiological studies reporting a strong protective effect of early-life adiposity on breast cancer risk (*13*–*24*). Simulations suggest that selection bias alone is unlikely to account for this association (*27*). Although selection bias may arise in these settings, it falls short of the effect sizes reported in earlier work. This study extends these earlier analyses by exploring adiposity at additional life stages. The attenuation of associations with postmenarche BMI after adjusting for prepubertal body size suggests that earlier adiposity may, at least in part, confound estimates for later periods. Given that the protective effect of early-life adiposity is unlikely to arise from selection bias alone, these results reinforce the importance of accounting for early-life exposures when evaluating causal effects across the lifecourse.

Evidence suggests that two distinct early-life pathways influence breast cancer risk: one linked to greater body size and the other to earlier age at menarche (*13*). While a larger body size in early life appears protective, it may also accelerate age at menarche, which is itself a risk factor for breast cancer, suggesting that these pathways are not only independent but may interact in complex ways. To isolate the direct effect of BMI independent of pubertal timing, we applied MVMR controlling for age at menarche. However, the genetic architecture of age at menarche and early-life adiposity overlaps substantially (*36*). A recent genome-wide structural equation model integrating MoBa childhood BMI, recalled adiposity at age 10 in UK Biobank, and age at menarche estimated that nearly half of the genetic variants associated with menarche act through early-life adiposity (*36*, *37*). This overlap has also been supported by longitudinal twin studies (*38*). While this indicates strong shared genetic pathways, a proportion of menarche-associated variants likely do act independently of adiposity. Nonetheless, BMI-related pathways to earlier menarche may contribute more strongly to identified SNP signals than non-BMI influences, complicating interpretation of MVMR estimates. Thus, although MVMR provides a valuable sensitivity analysis, it may not fully disentangle the independent contributions of adiposity and pubertal timing, particularly where shared pathways act differently across breast cancer subtypes. Furthermore, increased prepubertal body size has been shown to decrease breast density, while age at menarche has been linked to higher breast density, which is another important risk factor for breast cancer (*35*, *39*). This distinction between pathways highlights the need to examine potential mediating factors, which may help clarify the mechanisms at play. A deeper investigation into these separate but intersecting pathways could reveal previously unidentified insights into breast cancer risk.

The relationship between body size and breast cancer risk is further complicated by menopausal status. In conventional epidemiological settings, higher BMI has been linked to an increased risk of breast cancer in postmenopausal women and a decreased risk of breast cancer in premenopausal women (*40*–*43*). A plausible explanation includes the differing levels of oestrogen exposure between women experiencing overweight and normal weight (*44*). Premenopausal women experiencing overweight tend to have longer anovulatory cycles, which reduces their exposure to ovarian hormones, potentially lowering their breast cancer risk. After menopause, fat tissue serves as another source of oestrogen production, which increases breast cancer risk among overweight women (*45*–*48*). While our study did not specifically examine the relationship between overweight status and breast cancer pre- and postmenopause, effect estimates were broadly consistent across subtypes, with only modest differences in magnitude. The most evident departure from this pattern was seen in the HER2-enriched subtype, which showed little evidence of protection in either univariable or MVMR. This may reflect its distinct biology and the clinical observation that overweight and obesity can worsen prognosis in early-stage HER2-positive breast cancer (*49*). Lacking hormonal regulation, HER2-enriched tumors that depend on aberrant HER2 pathway activation may be less affected by adiposity-related changes in oestrogen, sex hormone-binding globulin (SHBG), or insulin-like growth factor 1 (IGF-1). In contrast, luminal B–like (HER2^+^) tumors appeared to retain strong evidence of a residual protective effect across the full reproductive-life window (menarche to <40 years), even after adjustment for prepubertal body size. This may reflect the continued influence of hormone-dependent pathways in luminal B–like (HER2^+^), which could mediate BMI-related protection through endocrine or metabolic mechanisms despite HER2 expression. Previous MR studies of childhood body size support such subtype-specific mechanisms; for example, mammographic density, particularly dense area, appears to mediate much of the protective effect for ER-positive but not ER-negative breast cancer (*35*), and hormonal mediators such as SHBG and IGF-1 appear relevant mainly for ER-positive disease (*13*). These findings underscore the importance of considering breast cancer subtypes when evaluating the long-term effects of adiposity across the lifecourse.

This study is an important analysis with multiple strengths. It focuses on a previously unexamined life stage—the period between menarche and under 40 years in nulliparous women—to investigate the impact of increased BMI on breast cancer risk using causal inference methods. By examining this life stage, we provide evidence on how adiposity before and during early reproductive life may influence breast cancer development. This focus also minimizes potential confounding from the physiological changes associated with a first pregnancy, allowing clearer interpretation of life stage–specific effects. To strengthen the analysis, we integrated data from five large European longitudinal cohort studies. This approach allowed us to gain key insights into the consistency of BMI-related genetic effects across different stages of life. In addition, these data are not only useful for this particular study but offer a valuable resource for future research into the effects of BMI in women at this life stage on other health outcomes.

This study has several limitations that should be considered when interpreting the findings. First, low conditional *F*-statistics were observed in the MVMR analysis, indicating potential for weak instrument bias. As a result, our MVMR findings should be interpreted with some caution. However, the pattern of attenuation observed when adjusting for prepubertal body size was not replicated when adjusting for later life adult body size, despite both exposures being derived from GWAS with similar sample sizes and instrument strength. This asymmetry supports the interpretation that the differences in effect estimates are unlikely to be driven by weak instrument bias alone and reflect biologically distinct influences of body size at different developmental stages. In addition, while we combined data from five large European longitudinal cohorts, the sample size remains smaller (*N* = 56,628) than that available for comparable measures of prepubertal and later life body size derived using UK Biobank data (*N* = 246,511). This reduced sample size may limit statistical power for detecting smaller effect sizes. Second, we used a prepubertal GWAS based on reported body size rather than measured BMI, as it offered a much larger sample size than any other GWAS of BMI at this life stage. However, this choice may distort MVMR analysis due to measurement error or differences in variance of effect sizes. Future work to precisely estimate childhood body size genetic effects are warranted. Third, there is the possibility of participant overlap between the HUNT and MoBa cohorts. The HUNT Study began in 1984–86, recruiting adults aged ≥20 in Nord-Trøndelag County across four cycles for over 30 years (*50*). Its Young-HUNT substudies recruited adolescents aged 13 to 19 in four waves between 1995 and 2019. MoBa, in contrast, recruited pregnant women nationally between 1999 and 2008 via routine prenatal care (*51*). Although up to 10% of MoBa participants resided in the Nord-Trøndelag catchment area, the differing recruitment criteria and time frames mean that any overlap with HUNT participants is expected to be minimal and unlikely to materially influence the analyses. Fourth, the inclusion criteria for this study resulted in substantially smaller analytic samples compared to the total number of participants enrolled in each cohort. Although genotyping was not based on specific phenotypes, we acknowledge that, as in all large-scale genetic studies, selection bias due to differential data availability may still be present. Last, as our analysis was restricted to individuals of European ancestry, the generalizability of our findings to other populations is limited. Further research is needed to confirm these findings in more diverse populations.

This study offers important insights into the genetic influences on BMI across different life stages and its causal relationship with breast cancer risk, focusing on the period between menarche and under 40 years in nulliparous women as a key window of susceptibility. While higher BMI in this interval appeared strongly protective against breast cancer in univariable analyses, the effect substantially, although not entirely, attenuated after accounting for childhood body size, used as a proxy for BMI. This pattern suggests that the protection may arise from the combined influence of greater adiposity in both childhood and early adulthood. These results have important implications for breast cancer prevention, underscoring earlier life stages as critical periods for potential interventions.

## MATERIALS AND METHODS

This study adheres to the Strengthening The Reporting of Observational Studies in Epidemiology Using Mendelian Randomisation guidelines (table S9).

### Study design and data sources

#### Participating cohorts

This study included five large, population-based prospective cohorts with available genomic and phenotypic BMI data from nulliparous women between menarche and age < 40 years: the ALSPAC, the HUNT, the MoBa, Generation R, and Generation Scotland. Participant selection is shown in Fig. 1.

*Avon Longitudinal Study of Parents and Children*. ALSPAC is a birth cohort which enrolled 14,541 pregnant women living in Avon, England with expected dates of delivery between 1 April 1991 and 31 December 1992 (the estimated recruitment rate of those eligible was 80%) (*52*–*54*). Further enrolments after 1998 resulted in a baseline sample of 14,901 children alive at 1 year of age. Some study data were collected and managed using Research Electronic Data Capture electronic data capture tools hosted at the University of Bristol (*55*). Please note that the study website contains details of all the data that are available through a fully searchable data dictionary and variable search tool: http://bristol.ac.uk/alspac/researchers/our-data/. We used data collected on women before pregnancy and their daughters in the 17- and 24-year follow up groups that had information on genotype, menarche, BMI, and age. We included 3125 ALSPAC mothers and 3496 daughters from the offspring generation in the age-stratified analysis. In the analysis focused on individuals between menarche and first birth (if pregnancy occurred within this timeframe), we included 3125 ALSPAC mothers and 1922 daughters.

*Trøndelag Health Study*. HUNT is a cohort study of the adult population in Trøndelag County, Norway and comprises ~230,000 individuals aged ≥20 years (*50*, *56*). Individuals were recruited in four surveys from 1984 to 2019, of which the HUNT2 and HUNT3 surveys were included in the present study. Invitee participation rates were 69 and 54%, respectively. Data on perinatal outcomes are available via linkage to the Medical Birth Registry of Norway (MBRN) (*57*) for individuals born from 1967 onward. The full recruited sample of mothers was 64,098. A total of 69,716 offspring were available in HUNT, with genotype and measured phenotype information, of which 36,938 were female (*58*, *59*). A total of 7683 individuals were used in this study.

*Norwegian Mother, Father and Child Cohort Study*. MoBa is a population-based pregnancy cohort study conducted by the Norwegian Institute of Public Health (*51*). Participants were recruited from all over Norway from 1999 to 2008, with 41% of all pregnant women invited consenting to participate. Blood samples were obtained from both parents during pregnancy and from mothers and children (umbilical cord) at birth (*60*). The first child was born in October 1999 and the last in July 2009. The cohort includes approximately 114,500 children, 95,200 mothers, and 75,200 fathers. In addition, MoBa was linked to data from the Medical Birth Registry of Norway (MBRN), a national health registry containing information about all births in Norway from 1967 onward (*57*). This analysis used a total of 31,471 MoBa first time mothers and 7216 daughters from the offspring generation with information on genotype, menarche, BMI, age, and parity.

*Generation R*. The Generation R Study is a population-based prospective cohort from fetal life onward in Rotterdam, the Netherlands (*61*). Women with an expected delivery date between April 2002 and January 2006 living in Rotterdam were eligible to enroll the study, and written informed consent was obtained from all participants. A total 9778 mothers, who gave birth to 9749 live-born children, enrolled in the study. Genotype data were available for a total of 7236 mothers, of which 3450 were first-time mothers of singleton children and had information on prepregnancy BMI (*62*). Maternal age and prepregnancy weight were reported by the mother, and height was measured at intake. A total of 2396 women contributed to this GWAS.

*Generation Scotland*. Generation Scotland is a population-based cohort with ~20,000 participants from across Scotland, aged between 18 and 98 years. During 2006 to 2010, potential participants (aged 35 to 65 years) were identified and invited to join at random from collaborating general medical practices in Scotland. Participants were asked to identify ≥1 first-degree relatives aged ≥18 years who would also be able to participate. Subsequently, participants attended a staffed research clinic, completed a health questionnaire, and had physical and clinical characteristics measured according to a standardized protocol. Fasting blood and urine samples were collected, according to standard operating procedures. The full recruited sample of female participants was 14,152, of which 3059 were included in this study and 2815 contributed to the GWAS.

#### Eligibility criteria and analytic sample

Given the focus of the research question, only female participants were included in the GWAS. Inclusion criteria required that participants had genotype data that passed quality control (QC), had reached menarche, and had BMI measured while still nulliparous. For mothers in birth cohorts, this corresponded to prepregnancy BMI at the time of the index pregnancy, provided they had not given birth previously. Participants also needed to have complete covariate information. Analyses were further restricted to individuals aged under 40 to focus on early adulthood and the reproductive years. To minimize potential population stratification bias, we further limited the sample to participants of European genetic ancestry.

### Genotyping, quality control, and imputation

Genotyping, QC, and imputation for each cohort are described in detail in the Supplementary Text and elsewhere (*59*, *62*–*64*). Briefly, participants were genotyped using genome-wide SNP arrays, and imputation was performed using standard reference panels following cohort-specific QC protocols. ALSPAC mothers and children were genotyped using Illumina Human660K and HumanHap550 arrays, respectively, with imputation via Haplotype Reference Consortium (HRC) v1.1. HUNT samples were genotyped using three Illumina HumanCoreExome arrays, with imputation performed using Minimac3 and a customized HRC v1.1 reference panel (*59*, *63*). MoBa samples were genotyped across 24 batches with varying selection criteria, centers, and arrays. The European Genome-Phenome Archive HRC v1.1 (study ID EGAS00001001710) was used for phasing and imputation (*51*, *64*). Generation R mothers were genotyped using Illumina GSA-MD 2.0 and 3.0 arrays, with imputation against the 1000 Genomes Phase 3 v5 reference panel (*61*, *62*). Generation Scotland samples were genotyped using the Illumina HumanOmniExpressExome-8v1 chip, with imputation via HRC.r1-1 on the Sanger Imputation Server.

### Statistical methods

#### Life stage–stratified GWAS and meta-analysis

We aimed to establish life stage–specific genetic effect profiles for BMI. This was to (i) assess the consistency of genetic variant effects on BMI across different life stages [including prepubertal and later life adulthood, where body size was used as a proxy for BMI based on previously derived GWAS from UK Biobank data (*14*)] and (ii) applying these profiles in downstream lifecourse MR analyses. To achieve this, we conducted GWAS on life stage–stratified BMI values across a life stage that had not previously been investigated. Specifically, separate GWAS analyses were run on nulliparous women between menarche and <40 years. These analyses were further stratified into three life stages: <20 years, 20 to <30 years, and 30 to <40 years. We define these as life stages, as they are anchored to each individual’s age at menarche—a biological milestone that varies between individuals—rather than being defined by absolute age alone. For BMI measurements from mothers in birth cohorts, we extracted prepregnancy data from women who had not given birth previously. For cohorts with repeat measures within a single life stage (e.g., menarche to <40 years), we selected the measure with the largest sample size (e.g., ALSPAC offspring BMI data from menarche to <20 year olds). A complete-case approach was used, excluding individuals with missing values from the analysis. Because of the skewed distribution of BMI in the data, a natural log transformation was applied before running the GWAS analyses within each cohort. Each cohort followed a shared GWAS framework using GCTA v1.93.2beta (*65*). While general QC guidelines and analysis scripts were provided via a GitHub repository, individual studies conducted preimputation QC independently, applying thresholds appropriate to their cohort data [Supplementary Text and elsewhere (*59*, *62*–*64*)]. All analyses were restricted post hoc to common genetic variants with a minor allele frequency ≥ 0.01. Genome-wide association analyses were conducted using GCTA’s fastGWA linear mixed model (*66*). To adjust for related individuals (e.g., mothers and daughters or other close relatives), a genetic relationship matrix (GRM) was constructed from participants and converted into a sparse GRM using a relatedness threshold of 0.05. Age and the top 20 genetic principal components were included as covariates, with the principal components adjusting for population structure.

We meta-analyzed summary statistics from the five cohorts for each life stage–specific GWAS separately, using a fixed effects model used in METAL version 2020-05-05 (*67*).

#### Heritability estimation, LD score regression, and genetic correlation analyses

We carried out further standard QC procedures (*68*) including tests for bias due to population structure [genomic control inflation factor (λ) and LDSC (*69*)]. We also estimated SNP heritability and assessed genetic correlations between our meta-analyzed GWAS and GWAS derived using UK Biobank data of prepubertal body size and later life adult body size (*14*). Analyses were performed using the LDSC software v1.0.0 (https://github.com/bulik/ldsc) using default parameters. We used LD scores from European participants of the 1000 genomes project that can be downloaded from the LDSC website. We generated Manhattan plots which contain horizontal lines drawn at −log_10_(1 × 10^−5^) for “suggestive associations” and −log_10_(5 × 10^−8^) for the “genome-wide significant” threshold and Quantile-Quantile (QQ) plots (figs. S2 to S5) (*70*).

We used LD clumping with an *r*^2^ threshold of 0.001 and a *P* value threshold of 5 × 10^−8^ to select a set of independent instruments for BMI at each life stage. This was performed using PLINK (*71*) and genotype data from European individuals from phase 3 v5 enrolled in the 1000 genomes project as a reference panel (*72*).

We visualized the life stage–specific effect trajectories of these SNPs at each life stage (menarche to <20 years, 20 to <30 years, and 30 to <40 years), with point size inversely proportional to the SE of the effect estimate. We focused exclusively on SNPs derived from the life stages analyzed in this study and did not include those from other studies due to differences in effect size scales. For example, body size measures for prepubertal and later adulthood stages, derived from UK Biobank data, were based on body size categories rather than BMI, making direct comparisons inconsistent (*14*).

#### External GWAS data sources

We used publicly available breast cancer data from the BCAC from 2017 (*N* = 228,951; ER^+^/ER^−^ samples, extracted from OpenGWAS under IDs: ieu-a-1127 and ieu-a-1128) (*28*) and the latest release of BCAC from 2020 [*N* = 247,173; overall sample and five molecular subtypes: luminal A–like, luminal B–like (HER2-positive), luminal B (HER2-negative-like), HER2-enriched, and triple-negative breast cancer] (*29*) (details in table S10). The 2017 dataset included 69,501 ER^+^ cases and 21,468 ER^−^ cases, with 105,974 shared controls. The 2020 dataset included 63,767 luminal A–like cases, 15,942 luminal B–like (HER2-positive) cases, 15,942 luminal B (HER2-negative)–like cases, 10,628 HER2-enriched cases, and 8602 triple-negative breast cancer cases, all with 91,477 controls. Luminal A–like tumors are characterized by ER^+^ and/or PR^+^, HER2^−^ status, and low histologic grade (grades 1 to 2); luminal B–like (HER2^+^) tumors are characterized by ER^+^ and/or PR^+^ and HER2^+^ status; luminal B/HER2-negative–like tumors are characterized by ER^+^ and/or PR^+^, HER2^−^ status, and high histologic grade (grade 3); HER2-enriched-like tumors are defined as HER2^+^, ER^−^, and PR^−^; and triple-negative tumors are defined as ER^−^, PR^−^, and HER2^−^. The consortium structure and genotyping procedures are described elsewhere (https://www.ccge.medschl.cam.ac.uk/ and https://www.ccge.medschl.cam.ac.uk/). The study groups in the BCAC consortium do not include ALSPAC, HUNT, MoBa, Generation R, Generation Scotland, or UK Biobank cohorts.

For comparative purposes, we utilized previously conducted GWASs of prepubertal and later life adult body size, used as a proxy for BMI, previously undertaken in the UK Biobank study on 246,511 females. These GWAS represent the largest available sample size for body size in these life stages. GWASs were adjusted for age and genotyping chip and genetic variants were selected on the basis of *P* < 5 × 10^−8^ and *r*^2^ < 0.001 (*14*, *73*, *74*). UK Biobank data were collected between 2006 and 2010 on individuals aged between 40 and 69 years old at baseline, including data from clinical examinations, assays of biological samples, detailed information on self-reported health characteristics, and genome-wide genotyping, using a prospective cohort study design (*74*). The prepubertal body size measure utilized recall questionnaire data, involving responses from adult participants who were asked whether, compared to the average, they were “thinner,” “about average,” or “plumper,” when they were aged 10 years old. The adult body size variable was derived using clinically measured BMI data (mean age, 56.5 years). It was then separated into a three-tier variable using the same categories as the childhood body size measure for comparability; “thinner” (21.1 to 25 kg/m^2^), “about average” (25 to 31.7 kg/m^2^), and “plumper” (31.7 to 59.9 kg/m^2^) (*14*). These scores have been independently validated in three distinct cohorts, providing verification that these genetic instruments can reliably separate childhood and adult BMI (*14*, *75*, *76*).

To disentangle whether our results were influenced by age at menarche, we used publicly available data [age when periods started (menarche)] from the UK Biobank cohort where female individuals were asked “How old were you when your periods started?”. This GWAS was conducted in 2014 (*N* = 182,416, extracted from OpenGWAS under ID: ieu-a-1095). To investigate the potential for collider bias resulting from restricting our analyses to nulliparous women, we used publicly available parity data (number of live births) from the UK Biobank cohort from 2018 (*N* = 250,782, extracted from OpenGWAS under ID: ukb-b-1209). This phenotype includes individuals with zero live births, thereby capturing variation from nulliparity onward and serving as a useful proxy for parity-related bias.

#### Lifecourse Mendelian randomization analysis

**Fig. 5.**
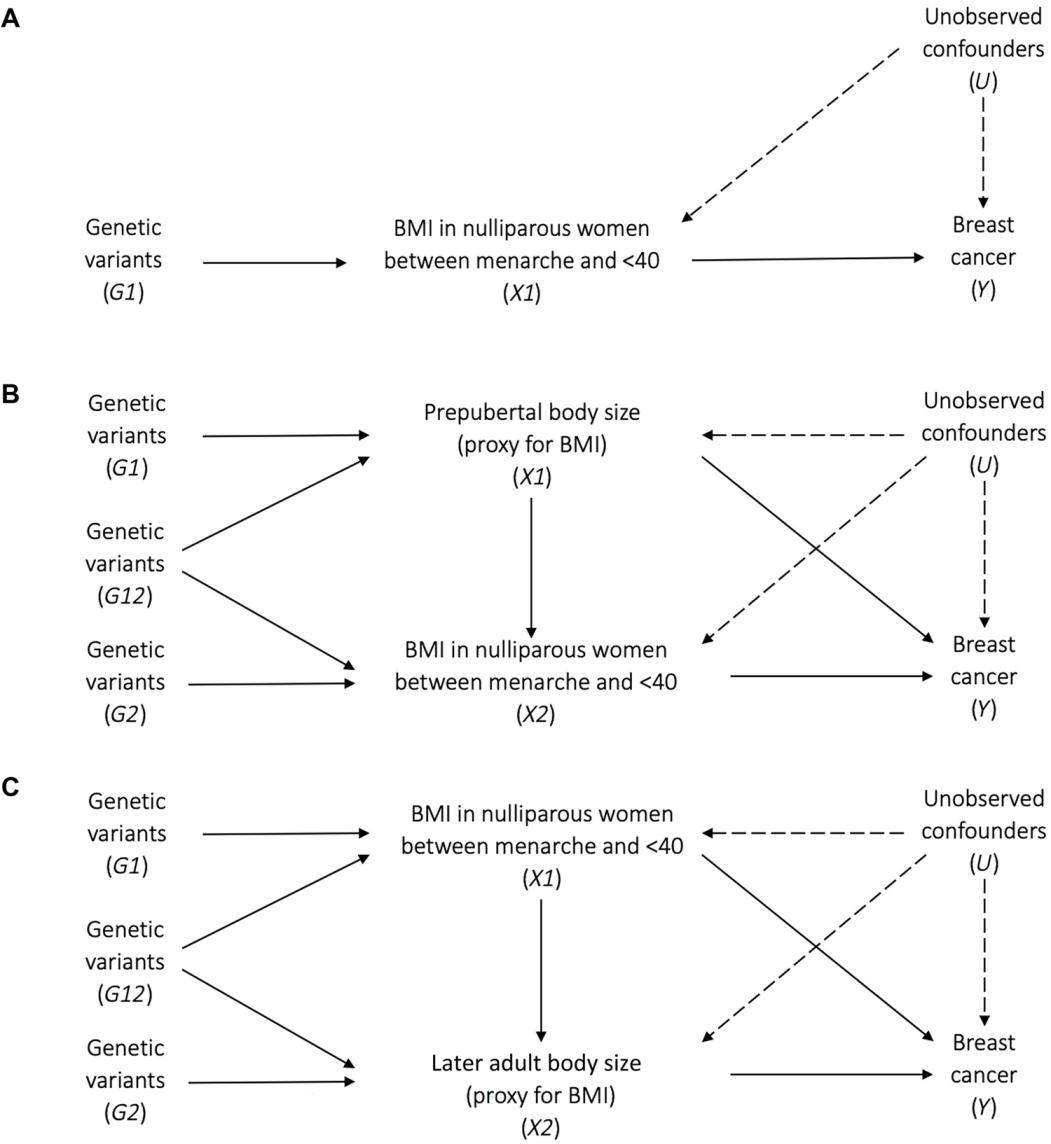
Directed acyclic graphs illustrating causal scenarios for life stage–specific BMI effects on breast cancer risk. Directed acyclic graphs indicating three scenarios describing how BMI in nulliparous women between menarche and <40 years may influence breast cancer risk in later life. (**A**) BMI between menarche and <40 years has a total causal effect on breast cancer risk. (**B**) BMI between menarche and <40 years has a direct causal effect on breast cancer risk after accounting for prepubertal body size. (**C**) BMI between menarche and <40 years has a direct causal effect on breast cancer risk after accounting for later-life adult body size. In all scenarios, unobserved confounders (U) may affect the exposures and the outcome. BMI, body mass index.

Our primary two-sample MR analyses used the IVW estimator, implemented in the TwoSampleMR R package (*77*). When genetic variants are used as instrumental variables in MR, the assumptions of instrumental variables must be met, i.e., the genetic variants used must (i) be strongly associated with the exposure of interest (“relevance”), (ii) not share common causes with the outcome (“independence”), and (iii) not affect the outcome other than through the exposure (“exclusion-restriction”) (*78*). We conducted sensitivity analyses, which relax the assumptions made about horizontal pleiotropy, including MR Egger regression (*79*), and the weighted median-based estimator (*80*). We also applied Steiger filtering (*81*) to test the direction of causality between BMI from menarche to first birth and breast cancer risk, by assessing whether the genetic instruments explained more variance in the exposure than in the outcome. In addition, we conducted analyses using the MR-RAPS method which is robust to weak instruments and systematic pleiotropy (*82*). For these MR-RAPS analyses, we applied a liberal SNP selection threshold of *P* < 1 × 10^−5^ to allow the inclusion of a larger set of instruments. We ran IVW MVMR, an extension of MR that employs multiple genetic variants associated with multiple measured risk factors, to calculate the direct and indirect effects of BMI between menarche and first birth on breast cancer outcomes (Fig. 5, A to C). For each MVMR model, we included all SNPs associated with either of the two traits, encompassing both trait-specific (G1 and G2) and shared (G12) variants, as illustrated in Fig. 5 (B and C). Genetic variants associated with each exposure were first pruned for LD to ensure instrument independence. The resulting SNP lists were then combined across exposures, and any duplicate variants present in both instruments were retained only once in the initial combined set. This full combined list was then repruned for LD to further ensure independence across all instruments used in the MVMR analysis. This final pruned set was used to extract harmonized summary statistics for all traits. As the MVMR framework accounts for correlations between exposures and accommodates pleiotropy, this approach supports estimation of exposure-specific effects and facilitates clearer interpretation of the independent contributions of each exposure.

This analysis accounted for prepubertal and later life adult body size separately (Fig. 5, B and C). These two additional time points were not included together in a single model due to the risk of weak instrument bias. Instrument strength was evaluated using the two-sample conditional *F*-statistic described elsewhere (*83*).

To improve interpretability and biological relevance, all MR effect estimates have been scaled to represent the effect per one SD increase in log-transformed BMI at each of the life stages. In addition, these estimates reflect the effect of genetic liability to BMI over particular life stages and should not be interpreted as equivalent to the effect size or duration of a time-limited intervention (*84*). This distinction is important when considering the clinical or public health implications of our findings.

*Sensitivity analyses*. To minimize pregnancy-related confounding, we restricted the BMI exposure GWAS to nulliparous women, enabling a clearer assessment of BMI’s direct influence across life stages. In contrast, the breast cancer outcome GWAS included both nulliparous and parous women. This discrepancy in selection mechanisms introduces potential selection bias in the genetic effect estimates for BMI but not for breast cancer. Specifically, conditioning on parity in the BMI GWAS may induce bias in the estimated SNP effects on BMI (${\hat{\beta }}_{\text{Gx}}$), whereas breast cancer estimates (${\hat{\beta }}_{\text{Gy}}$), remain unaffected as parity was not conditioned on in the outcome GWAS (Eq. 1).

In this two-sample MR setting, the MR estimate is given by

$${\hat{\beta }}_{\text{MR}}=\frac{{\hat{\beta }}_{\text{Gy}}}{{\hat{\beta }}_{\text{Gx}}}$$ (1)

where

$$E\left({\hat{\beta }}_{\text{Gx}}\right)=\beta_{\text{Gx}}+\text{bias}$$

$$E\left({\hat{\beta }}_{\text{Gy}}\right)=\beta_{\text{Gy}}$$

Since ${\hat{\beta }}_{\text{Gy}}$ is not subject to the same selection mechanism and there is no reason to assume that the selection-induced bias in $\beta_{\text{Gx}}$ is correlated with ${\hat{\beta }}_{\text{Gy}}$, a major spurious association in the MR analysis is unlikely. However, selection bias in the BMI GWAS could distort the MR estimate, with the direction and magnitude depending on whether and how BMI influences parity within the restricted sample. In an extreme scenario, it could induce false-positive associations with BMI that arise due to collider bias although this is unlikely at current sample sizes.

To empirically assess this, we performed univariable MR analyses estimating the effect of parity on BMI in nulliparous women across different life stages (menarche to <40 years, <20 years, 20 to <30 years, and 30 to <40 years). While parity itself cannot causally influence BMI in nulliparous women, genetic variants associated with parity may exhibit pleiotropic effects on BMI through shared metabolic and reproductive pathways. If these parity-associated variants also influence BMI within our restricted sample, it would suggest selection bias related to reproductive behavior, implying that conditioning on parity in the BMI GWAS may have introduced collider bias. We additionally conducted the reverse analysis, estimating the effect of early-life BMI-associated variants on parity, to assess the potential for bias in the opposite direction.

In addition, a later onset of menarche has been linked to a reduced risk of breast cancer (*85*, *86*). Childhood body size has been shown to accelerate the timing of menarche, while an earlier menarche increases the likelihood of increased body size in adulthood (*87*, *88*). With these traits sharing a complex and interconnected relationship, we conduct sensitivity analyses estimating the effect of BMI in the life stages analyzed in nulliparous women on overall breast cancer accounting for age at menarche in MVMR analyses. Age at menarche is treated as a confounder, given its influence on both BMI and breast cancer risk through hormonal and metabolic pathways. Including it in the MVMR model ensures that observed BMI effects are not simply a reflection of differences in pubertal timing. However, we note that MVMR adjustment for age at menarche is complicated by the substantial genetic overlap between prepubertal BMI and menarche timing (*36*). We conducted statistical analyses in R version 4.3.3 (*89*).

### Research ethics and informed consent

Informed consent for cohort participation was obtained from all participants, and ethical approval was obtained from the Regional Committee for Medical and Health Research Ethics, Central Norway (REK Central application number 2018/2488) (HUNT), the ALSPAC Ethics and Law Committee and the Local Research Ethics Committees (ALSPAC), and the Medical Ethical Committee of Erasmus MC, University Medical Center Rotterdam, approved the study (MEC 198.782/2001/31) (Generation R), and The Regional Committees for Medical and Health Research Ethics (REK application number 2016/1702) (MoBa). Written consent was obtained from all participants in Generation Scotland. All components of Generation Scotland received ethical approval from the NHS Tayside Committee on Medical Research Ethics (REC reference number: 05/S1401/89). Generation Scotland has also been granted Research Tissue Bank status by the East of Scotland Research Ethics Service (REC reference number: 20-ES-0021).

## Supplementary Materials

### This PDF file includes:

Supplementary Text Figs. S1 to S5 Tables S1 to S10 References
