# Supplementary material for: Lifecourse genome-wide association study meta-analysis refines the critical life stages for adiposity’s influence on breast cancer risk
Source: Sci Adv. 2026 Jan 21;12(4):eady0374. doi: 10.1126/sciadv.ady0374 (PMC12822656; doi:10.1126/sciadv.ady0374)
Supplement: Supplementary file 1 — Supplementary Text Figs. S1 to S5 Tables S1 to S10 References [file sciadv.ady0374_sm.pdf]

Science Advances
AAAS

Supplementary Materials for

Lifecourse genome-wide association study meta-analysis refines the critical life stages for adiposity's influence on breast cancer risk

Grace M. Power *et al.*

Corresponding author: Grace M. Power, grace.power@bristol.ac.uk

Sci. Adv. **12**, eady0374 (2026)
DOI: 10.1126/sciadv.ady0374

This PDF file includes:

Supplementary Text
Figs. S1 to S5
Tables S1 to S10
References

Supplementary Text. Further information on genotyping, quality control and imputation in each cohort.

ALSPAC mothers were genotyped using the Illumina Human660K quad single nucleotide polymorphism (SNP) chip, and ALSPAC children using the Illumina HumanHap550 quad genome-wide SNP genotyping platform (91). ALSPAC fathers were genotypes using the 1000 Genomes phase 1 panel (92). Genotype data for all groups were imputed using the Haplotype Reference Consortium v1.1 reference panel after applying QC filters that excluded SNPs with minor allele frequency (MAF) ≤1%, call rate ≤95%, and deviation from Hardy–Weinberg equilibrium (HWE). Samples were further excluded based on incorrect sex assignment, evidence of cryptic relatedness, and non-European ancestry.

HUNT samples were genotyped using one of three different Illumina HumanCoreExome arrays (HumanCoreExome12 v1.0, HumanCoreExome12 v1.1, and UM HUNT Biobank v1.0) (59, 63). Genomic positions, strand orientation, and reference alleles were determined by aligning probe sequences against the human genome (Genome Reference Consortium build 37 and revised Cambridge Reference Sequence of mitochondrial DNA; http://genome.ucsc.edu) using BLAT (93). Ancestry was inferred by projecting genotyped samples onto principal components derived from the Human Genome Diversity Project (HGDP) reference panel (938 unrelated individuals; downloaded from http://csg.sph.umich.edu/chaolong/LASER/) (94, 95) using PLINK v1.9039 (71). The resulting genotype data were phased using Eagle2 v2.340 (96). Imputation was performed on the samples of recent European ancestry using Minimac3 (v2.0.1, http://genome.sph.umich.edu/wiki/Minimac3)(97) with default settings (2.5 Mb reference based chunking with 500 kb windows) and a customized Haplotype Reference consortium release 1.1 (HRC v1.1) for autosomal variants and HRC v1.1 for chromosome X variants (91). SNPs with MAF <1% and call rate <95% were excluded, and samples were removed if they showed excess heterozygosity (±3 standard deviations from the mean) or were identified as ancestral outliers based on principal component analysis. Additionally, deviations from Hardy-Weinberg equilibrium ($p < 1\times10^{-6}$) led to SNP exclusion to maintain genotype quality. This process was explained previously (58).

MoBa samples were genotyped through several research projects, across 24 genotyping batches with varying selection criteria, genotyping centers, and genotyping arrays. Detailed information on batch selection criteria, genotyping, pre-imputation quality control (QC),

phasing, imputation, and post-imputation QC are described in full elsewhere (64). The establishment of MoBa and initial data collection was based on a license from the Norwegian Data Protection Agency and approval from The Regional Committees for Medical and Health Research Ethics (REK). The MoBa cohort is currently regulated by the Norwegian Health Registry Act. The MoBa analyses of BMI were carried out as part of the Lifecourse GWAS Consortium through the PsychGen multimorbidity project (REK 2016/1702), and in contribution to the project “Combining Mendelian Randomisation and Depression Trajectories to Better Inform Intervention Timing” (90).

Generation Scotland samples were genotyped using the Illumina HumanOmniExpressExome-8v1 chip, and genotype calling was performed with the Beadstudio-Gencall v3 algorithm. QC excluded SNPs with MAF <1%, call rate <98%, and deviation from HWE (p-value $<1\times10^{-6}$). Samples were excluded if they had a call rate <98%. Phasing was conducted using SHAPEIT2 with the duoHMM option, and imputation was performed using the Haplotype Reference Consortium (HRC.r1-1) reference panel via the Sanger Imputation Server. Post-imputation filtering removed SNPs with an imputation quality score <0.4, duplicate variants, and monomorphic SNPs.

Generation R mothers were genotyped using the Illumina GSA-MD 2.0 and 3.0 arrays (62). Genotype data were imputed against the 1000 Genomes Phase 3 v5 reference panel following QC filters that excluded SNPs with MAF <0.01%, call rate <99%, and deviation from HWE. Samples were further excluded for incorrect sex assignment, genetic duplicates, and non-European ancestry.

Figure S1. Age-Specific SNP Effect Trajectories on BMI. Plot illustrating the age-specific effect trajectories of individual SNPs on BMI across three life-stage categories, menarche to <20 years, 20 to <30 years, and 30 to <40 years, in nulliparous women. Each line in the plot represents a single SNP, tracing its effect size (beta) across these age intervals. The point sizes are inversely proportional to the standard error of the effect estimates, meaning larger points indicate stronger associations with greater weight in the heterogeneity test. The plot facets divide SNPs based on whether they exhibit significant heterogeneity (Q FDR < 0.05) or not (Q FDR ≥ 0.05).

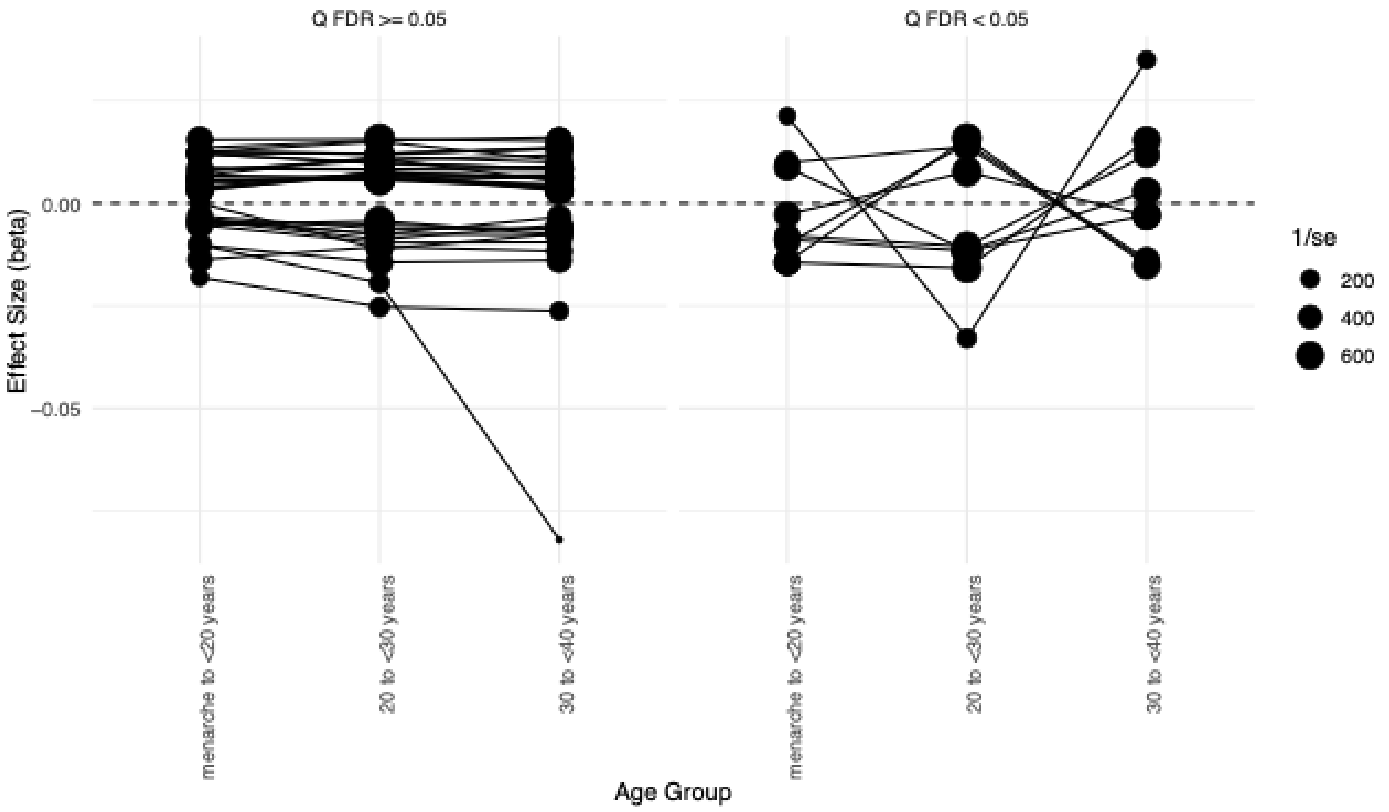

Figure S2A. Manhattan Plot for Meta-Analysed GWAS of Body Mass Index Between Menarche and <40 Years. Manhattan plot displaying the results of the meta-analysed genome-wide association study (GWAS) for body mass index (BMI) in nulliparous women between menarche and <40 years. The x-axis represents genomic position by chromosome, and the y-axis represents the –log10(p-values) of the SNPs. Horizontal lines indicate the thresholds for suggestive associations (–log10(1×10^{-5}), blue) and genome-wide significance (–log10(5×10^{-8}), red).

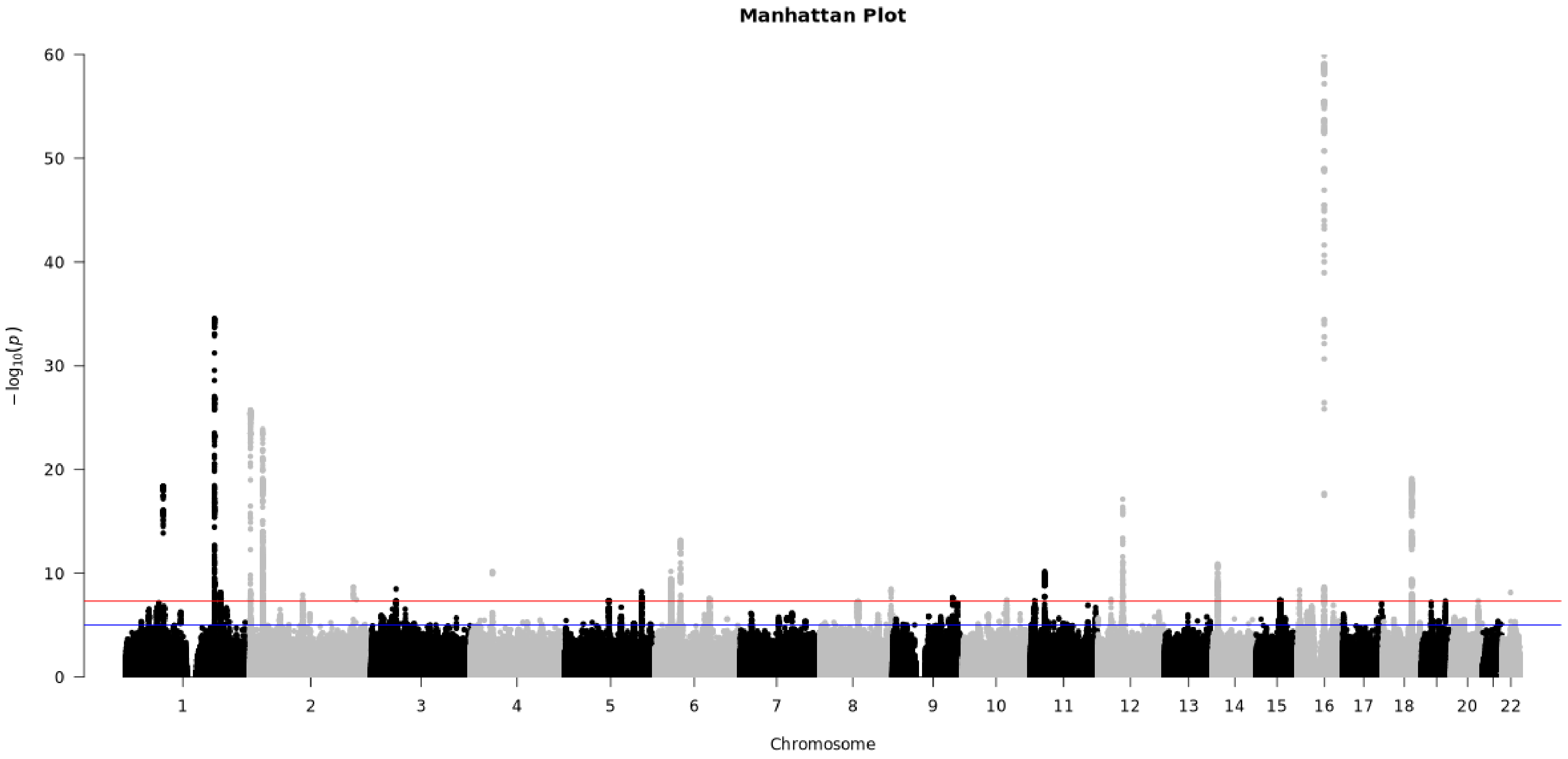

Figure S2B. QQ Plot for Meta-Analysed GWAS of Body Mass Index Between Menarche and <40 Years. QQ plot illustrating the observed versus expected –log10(p-values) from the meta-analysed genome-wide association study (GWAS) for body mass index (BMI) in nulliparous women between menarche and <40 years. The red line represents the null hypothesis of no association.

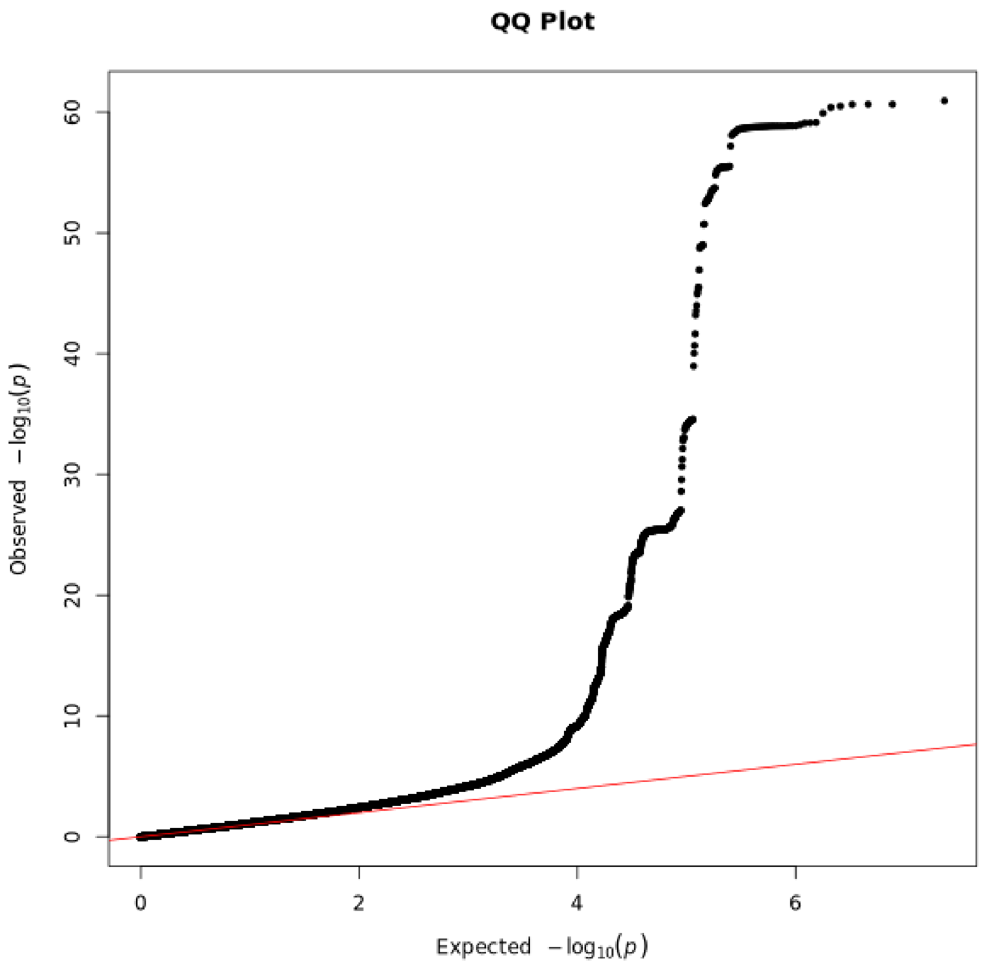

Figure S3A. Manhattan Plot for Meta-Analysed GWAS of Body Mass Index Between Menarche and <20 Years. Manhattan plot displaying the results of the meta-analysed genome-wide association study (GWAS) for body mass index (BMI) in nulliparous women between menarche and <20 years. The x-axis represents genomic position by chromosome, and the y-axis represents the –log10(p-values) of the SNPs. Horizontal lines indicate the thresholds for suggestive associations (–log10(1×10^{-5}), blue) and genome-wide significance (–log10(5×10^{-8}), red).

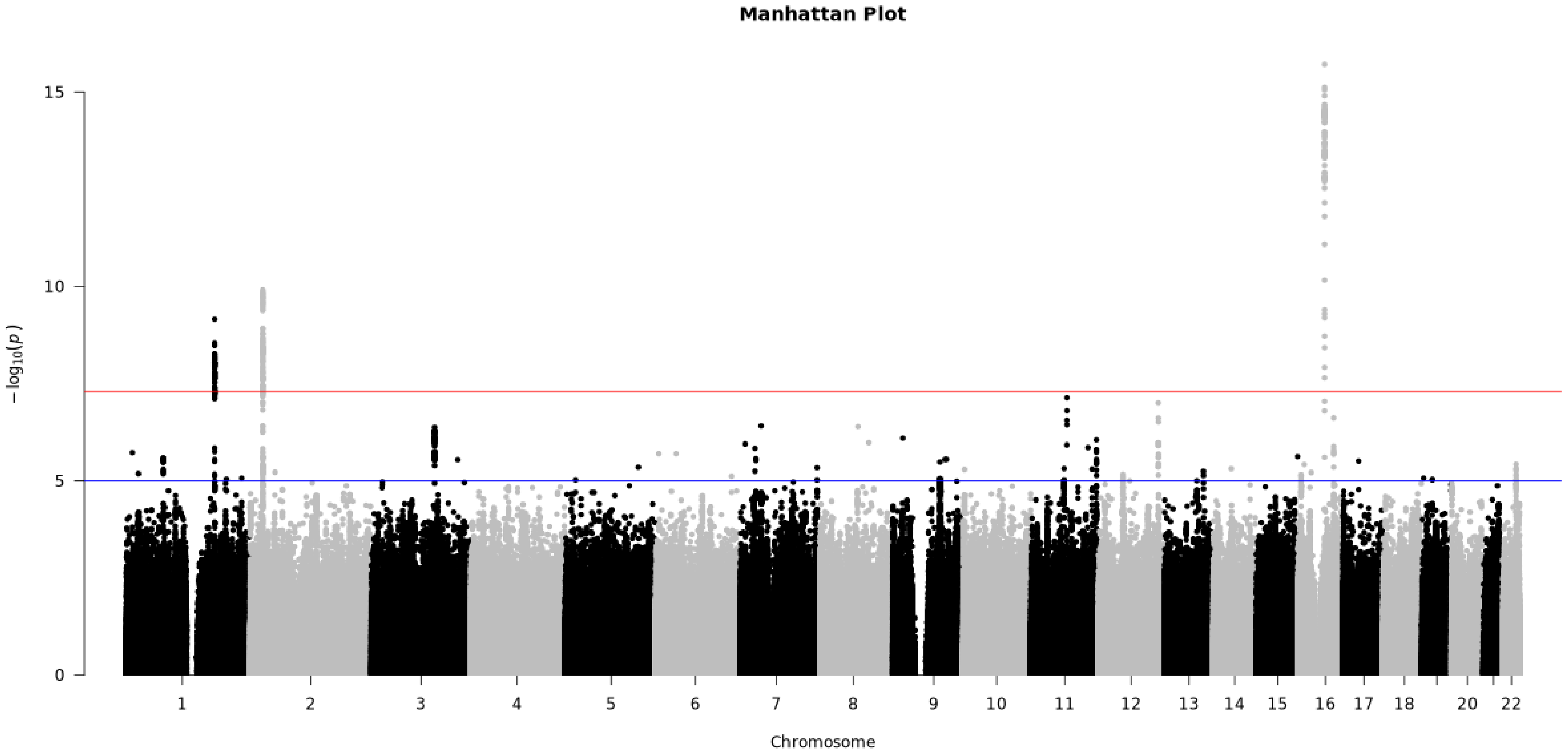

Figure S3B. QQ Plot for Meta-Analysed GWAS of Body Mass Index Between Menarche and <20 Years. QQ plot illustrating the observed versus expected –log10(p-values) from the meta-analysed genome-wide association study (GWAS) for body mass index (BMI) in nulliparous women between menarche and <20 years. The red line represents the null hypothesis of no association.

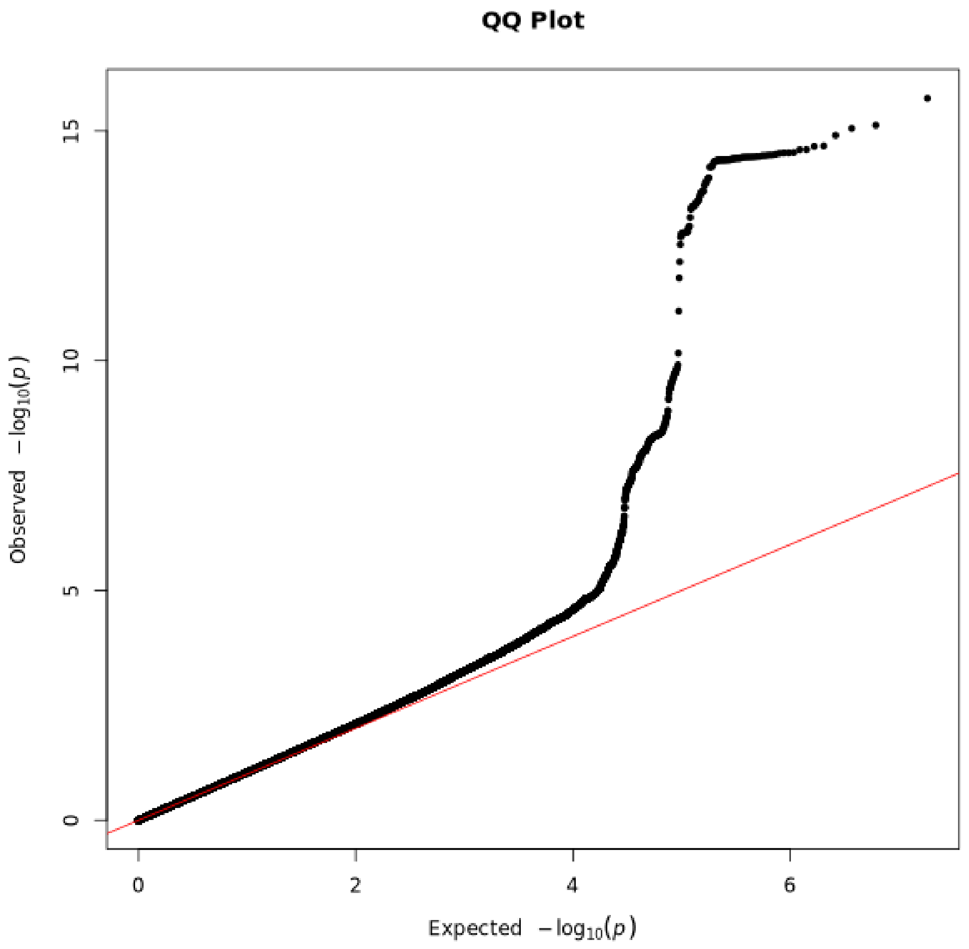

Figure S4A. Manhattan Plot for Meta-Analysed GWAS of Body Mass Index Between 20 and <30 Years. Manhattan plot displaying the results of the meta-analysed genome-wide association study (GWAS) for body mass index (BMI) in nulliparous women between 20 and <30 years. The x-axis represents genomic position by chromosome, and the y-axis represents the –log10(p-values) of the SNPs. Horizontal lines indicate the thresholds for suggestive associations (–log10(1×10^{-5}), blue) and genome-wide significance (–log10(5×10^{-8}), red).

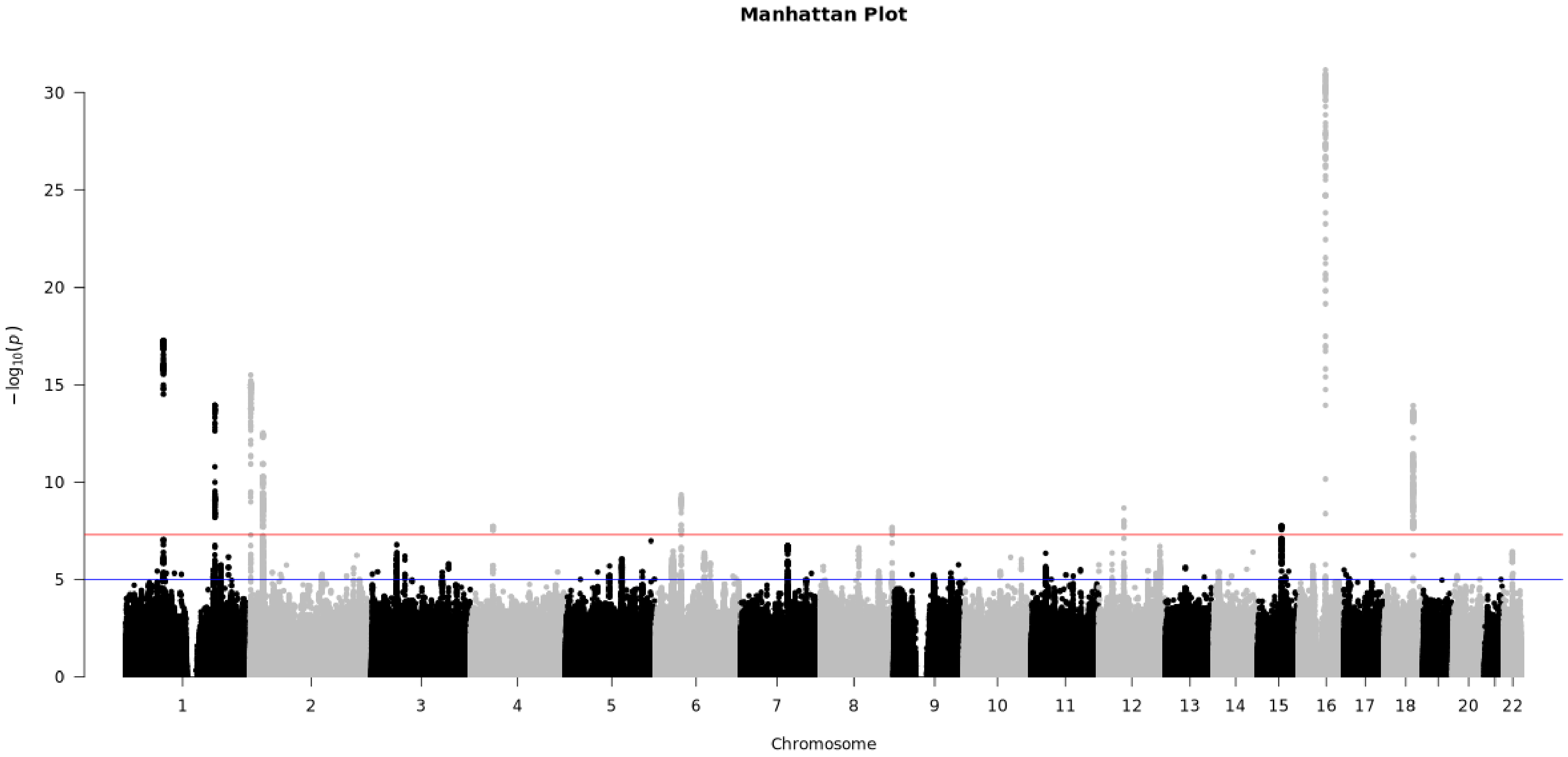

Figure S4B. QQ Plot for Meta-Analysed GWAS of Body Mass Index Between 20 and <30 Years. QQ plot illustrating the observed versus expected –log10(p-values) from the meta-analysed genome-wide association study (GWAS) for body mass index (BMI) in nulliparous women between 20 and <30 years. The red line represents the null hypothesis of no association.

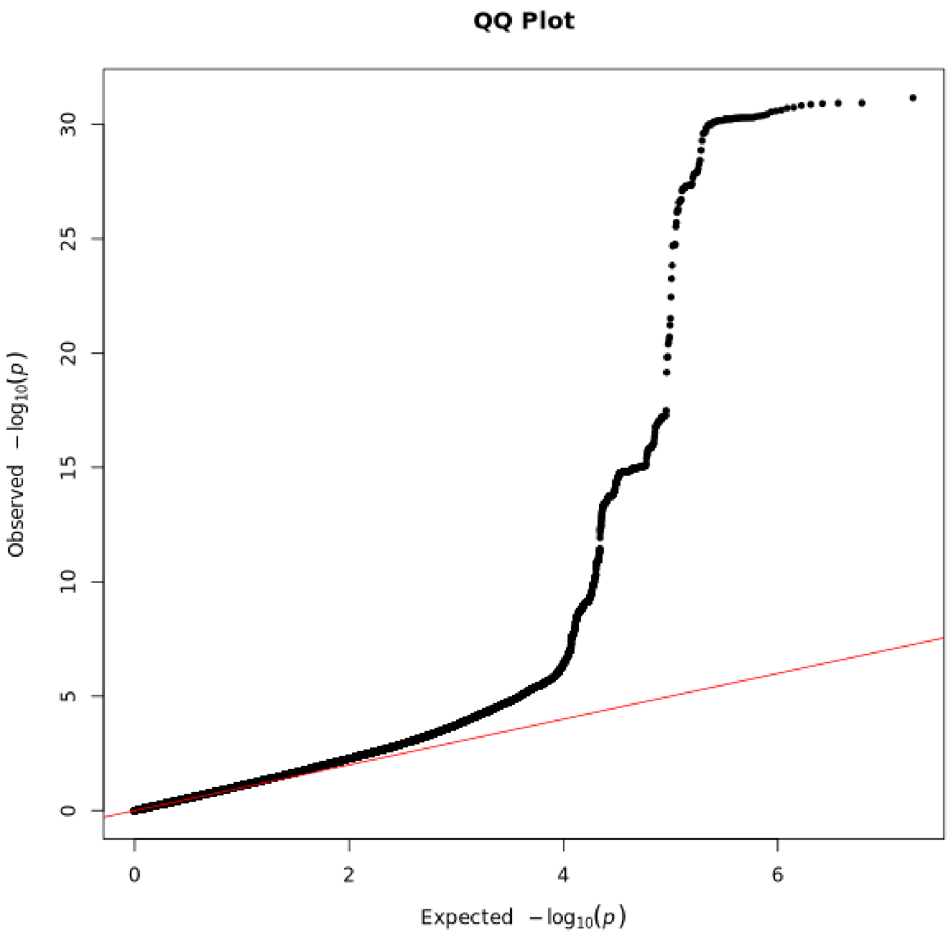

Figure S5A. Manhattan Plot for Meta-Analysed GWAS of Body Mass Index Between 30 and <40 Years. Manhattan plot displaying the results of the meta-analysed genome-wide association study (GWAS) for body mass index (BMI) in nulliparous women between 30 and <40 years. The x-axis represents genomic position by chromosome, and the y-axis represents the –log10(p-values) of the SNPs. Horizontal lines indicate the thresholds for suggestive associations (–log10(1 × 10^{-5}), blue) and genome-wide significance (–log10(5 × 10^{-8}), red).

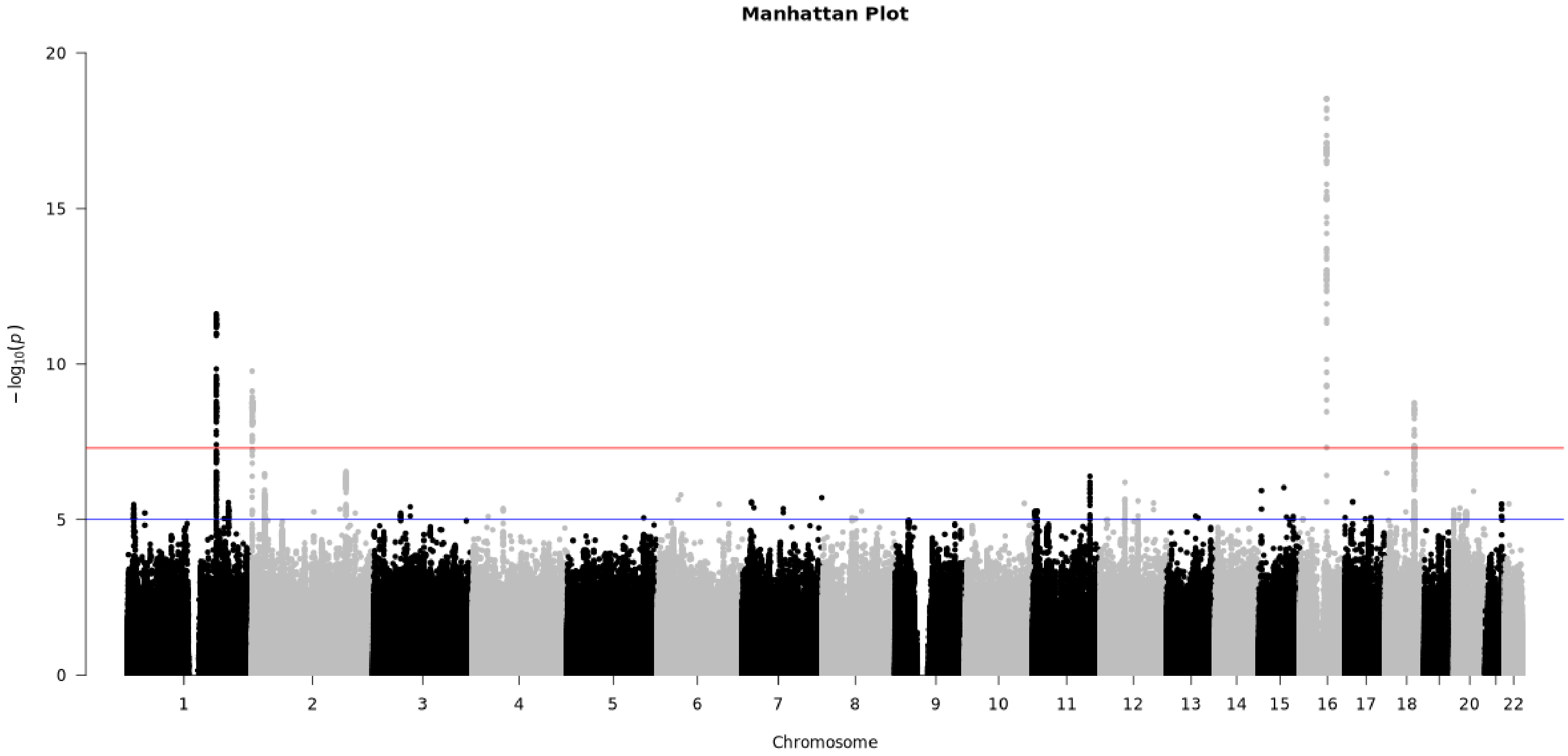

Figure S5B. QQ Plot for Meta-Analysed GWAS of Body Mass Index Between 30 and <40 Years. QQ plot illustrating the observed versus expected –log10(p-values) from the meta-analysed genome-wide association study (GWAS) for body mass index (BMI) in nulliparous women between 30 and <40 years. The red line represents the null hypothesis of no association.

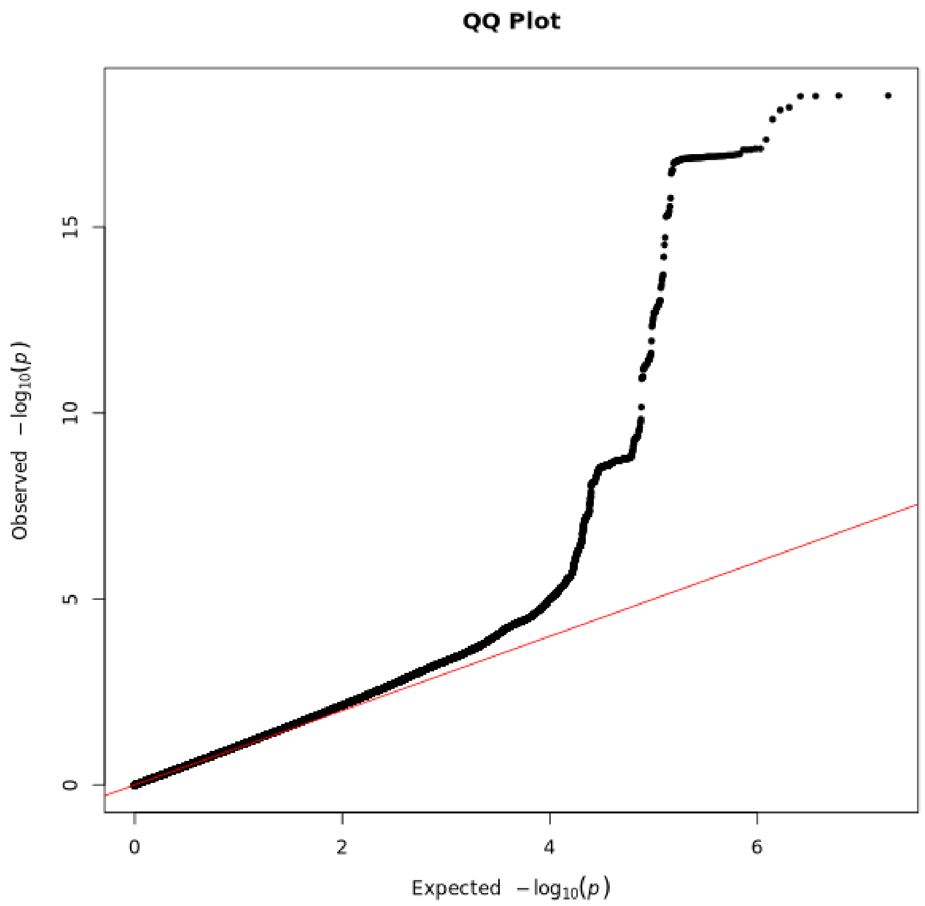

Table S1. Meta-Analysed Genome-Wide Association Study Results for Body Mass Index Across Life-Stage Categories. Meta-analysed genome-wide association study (GWAS) results for body mass index (BMI) in nulliparous women between menarche and <40 years, menarche and <20 years, 20 and <30 years, and 30 and <40 years.

SNP - single nucleotide polymorphism identifier, beta^ - effect estimate coefficient for SNP on log-transformed BMI, beta - effect estimate coefficient for SNP on body size category, SE- standard error of the effect estimate, P - corresponding p-value, * - indicates SNPs retained in the final set identified through LD clumping across all time windows.

- (orange) Independent discovery SNPs for BMI in nulliparous women between menarche and <40 years
- (green) Independent discovery SNPs for BMI in nulliparous women between menarche and <20 years
- (blue) Independent discovery SNPs for BMI in nulliparous women between 20 and <30 years
- (pink) Independent discovery SNPs for BMI in nulliparous women between 30 and <40 years

							Age group cateogry																							
							BMI in nulliparous women between menarche and <40 years				BMI in nulliparous women between menarche and <20 years				BMI in nulliparous women between 20 and <30 years				BMI in nulliparous women between 30 and <40 years				Prepubertal body size (Richardson et al.)				Later life adult body size (Richardson et al.)			
SNP	Chromosome	Base position (build hg19)	Base position (build hg38)	Closest gene	Effect allele	Other allele	Effect allele frequency	Beta^	Standard Error	P	Effect allele frequency	Beta^	Standard Error	P	Effect allele frequency	Beta^	Standard Error	P	Effect allele frequency	Beta^	Standard Error	P	Effect allele frequency	Beta	Standard Error	P	Effect allele frequency	Beta	Standard Error	P
rs62033406	16	53824226	53790314	FTO	G	A	0.418	0.016	0.001	1.15E-61	0.418	0.016	0.002	1.95E-16	0.421	0.016	0.001	2.63E-31	0.417	0.015	0.002	1.26E-18	0.408	0.045	0.002	8.80E-118	0.408	0.042	0.002	1.20E-98
rs7707628*	5	153546900	154167340	MFAP3	C	T	0.664	-0.006	0.001	6.17E-09	0.669	-0.003	0.002	1.02E-01	0.665	-0.006	0.001	6.74E-06	0.656	-0.006	0.002	2.87E-04	0.646	-0.015	0.002	9.20E-14	0.646	-0.011	0.002	1.10E-07
rs60186497*	14	30479220	30010014	PRKD1	T	C	0.037	0.014	0.003	2.76E-08	0.039	0.013	0.005	1.16E-02	0.036	0.015	0.004	2.96E-05	0.037	0.011	0.005	1.98E-02	0.044	0.030	0.005	2.70E-10	0.044	0.021	0.005	1.50E-05
rs2601781*	16	4017392	3967391	ADCY9	A	T	0.632	0.006	0.001	4.20E-09	0.631	0.006	0.002	2.70E-03	0.631	0.006	0.001	1.56E-05	0.633	0.004	0.002	1.80E-02	0.618	0.017	0.002	4.20E-18	0.618	0.016	0.002	2.10E-14
rs13130484*	4	45175691	45173674	THAP12P9; PRDX4P1	T	C	0.414	0.006	0.001	7.04E-11	0.415	0.004	0.002	4.59E-02	0.413	0.008	0.001	1.34E-08	0.419	0.006	0.002	1.18E-04	0.432	0.023	0.002	4.20E-32	0.432	0.017	0.002	7.80E-17
rs6098816*	20	54385642	55810586	CBLN4	A	C	0.174	-0.007	0.001	4.74E-08	0.173	-0.004	0.003	1.34E-01	0.173	-0.008	0.002	2.72E-05	0.174	-0.003	0.002	1.57E-01	0.808	-0.015	0.002	4.80E-09	0.808	-0.002	0.002	4.00E-01
rs28693508*	9	120361418	117599140	TLR4	G	C	0.354	-0.005	0.001	2.19E-08	0.351	-0.005	0.002	2.09E-02	0.354	-0.005	0.001	1.82E-04	0.352	-0.007	0.002	2.79E-04	0.339	-0.011	0.002	1.10E-07	0.339	-0.009	0.002	1.10E-05
rs62155011*	2	105565719	104949261	MRPS9	C	T	0.404	0.006	0.001	1.19E-08	0.411	0.006	0.002	4.21E-03	0.405	0.006	0.001	8.57E-06	0.395	0.005	0.002	8.91E-03	0.375	0.004	0.002	3.30E-02	0.375	0.007	0.002	9.10E-04
rs574367*	1	177873210	177904075	LINC01741, SEC16B	T	G	0.226	0.014	0.001	2.82E-35	0.226	0.013	0.002	2.22E-08	0.227	0.012	0.002	1.24E-14	0.221	0.013	0.002	8.58E-12	0.209	0.053	0.002	1.30E-112	0.209	0.036	0.002	6.60E-51
rs59086897*	2	25145173	24922304	ADCY3,DNAJC27	A	T	0.489	0.010	0.001	1.27E-24	0.494	0.012	0.002	1.38E-10	0.489	0.010	0.001	4.85E-14	0.487	0.008	0.002	3.181E-07	0.487	0.040	0.002	1.30E-95	0.487	0.024	0.002	2.60E-34
rs10191896*	2	213421764	212557040	ERBB4	T	C	0.371	-0.010	0.002	3.50E-08	0.362	0.000	0.004	9.94E-01	0.368	-0.011	0.002	5.95E-07	0.375	-0.007	0.003	1.59E-02	0.376	-0.001	0.002	5.60E-01	0.376	-0.005	0.002	1.00E-02
rs11615823*	12	26815139	26662206	BHLHE41	C	T	0.031	-0.015	0.003	3.17E-08	0.033	-0.010	0.005	5.29E-02	0.031	-0.019	0.004	4.40E-07	0.031	-0.082	0.143	5.67E-01	0.030	-0.003	0.006	6.80E-01	0.030	-0.010	0.006	9.40E-02
rs2764261*	6	108927842	108606639	FOXO3	G	A	0.632	0.005	0.001	2.52E-08	0.628	0.006	0.002	2.74E-03	0.637	0.006	0.001	4.35E-05	0.627	0.004	0.002	2.18E-02	0.625	0.008	0.002	4.00E-05	0.625	0.012	0.002	1.40E-08

rs7613360*	3	49916710	49879277	MST1R,ACTL11P	T	C	0.392	0.006	0.001	3.22E-09	0.392	0.003	0.002	8.60E-02	0.397	0.007	0.001	1.56E-07	0.385	0.004	0.002	2.60E-02	0.394	0.005	0.002	1.60E-02	0.394	0.014	0.002	3.90E-12
rs13387091*	2	650980	650980	LINC01875, TMEM18	A	G	0.166	-0.013	0.001	1.83E-26	0.167	-0.010	0.003	3.90E-05	0.165	-0.014	0.002	3.40E-16	0.169	-0.014	0.002	1.95E-10	0.172	-0.044	0.003	1.20E-67	0.172	-0.036	0.003	1.40E-43
rs6494678*	15	67946247	67653909	MAP2K5	T	C	0.241	-0.006	0.001	3.88E-08	0.239	-0.004	0.002	5.77E-02	0.240	-0.009	0.002	1.80E-08	0.236	-0.005	0.002	8.62E-03	0.254	-0.021	0.002	1.10E-20	0.254	-0.016	0.002	1.20E-12
rs114670539*	2	207064335	206199611	CMKLR2	T	C	0.041	0.014	0.002	2.08E-09	0.040	0.014	0.005	6.06E-03	0.041	0.015	0.003	4.55E-06	0.045	0.016	0.004	6.94E-05	0.058	0.041	0.004	9.80E-23	0.058	0.007	0.004	8.60E-02
rs7442885*	5	87682877	88387060	TMEM161B-DT	G	C	0.197	-0.007	0.001	4.31E-08	0.199	-0.004	0.002	8.42E-02	0.198	-0.008	0.002	4.55E-06	0.197	-0.008	0.002	1.97E-04	0.214	-0.012	0.002	2.00E-06	0.214	-0.017	0.002	1.50E-12
rs7462788*	8	143335170	142253809	PTK2	T	G	0.398	0.006	0.001	3.34E-09	0.398	0.003	0.002	1.17E-01	0.398	0.008	0.001	2.40E-08	0.394	0.003	0.002	8.67E-02	0.395	0.003	0.002	1.00E-01	0.395	0.008	0.002	7.70E-05
rs10840050*	11	8473707	8452160	STK33	A	G	0.365	-0.005	0.001	4.39E-08	0.362	-0.005	0.002	6.17E-03	0.363	-0.004	0.001	2.12E-03	0.367	-0.008	0.002	1.14E-05	0.373	-0.006	0.002	1.20E-03	0.373	-0.011	0.002	1.00E-07
rs2180454*	14	29690513	29221307	LINC02327	C	T	0.734	0.007	0.001	1.28E-11	0.733	0.005	0.002	1.36E-02	0.737	0.007	0.002	4.66E-06	0.731	0.007	0.002	3.92E-04	0.772	0.006	0.002	4.60E-03	0.772	0.012	0.002	1.10E-07
rs3115665*	6	31589264	31621487	PRRC2A	C	G	0.805	-0.009	0.001	6.47E-11	0.802	-0.005	0.003	3.47E-02	0.805	-0.010	0.002	5.00E-07	0.806	-0.010	0.002	3.56E-05	0.804	-0.016	0.002	2.00E-11	0.804	-0.014	0.002	7.00E-09
rs815339*	1	190116575	190147445	BRINP3	A	T	0.488	0.005	0.001	7.31E-09	0.484	0.007	0.002	3.24E-04	0.490	0.006	0.001	1.47E-06	0.486	0.003	0.002	5.00E-02	0.505	0.011	0.002	2.50E-08	0.505	0.011	0.002	1.40E-08
rs2076308*	6	50791640	50823927	TFAP2B	C	G	0.185	0.009	0.001	6.74E-14	0.187	0.008	0.002	1.22E-03	0.183	0.011	0.002	1.27E-10	0.188	0.005	0.002	1.08E-02	0.180	0.027	0.003	8.90E-27	0.180	0.025	0.003	3.90E-23
rs1902666*	10	88011779	86252022	GRID1	T	C	0.885	0.008	0.002	3.92E-08	0.888	0.009	0.003	3.09E-03	0.887	0.008	0.002	8.11E-05	0.884	0.009	0.003	6.90E-04	0.870	0.007	0.003	2.10E-02	0.870	0.006	0.003	2.90E-02
rs3810291*	19	47569003	47065746	ZC3H4	A	G	0.677	0.006	0.001	4.82E-08	0.677	0.006	0.002	9.27E-03	0.677	0.006	0.002	1.21E-04	0.677	0.006	0.002	6.44E-04	0.676	0.015	0.002	2.60E-13	0.676	0.015	0.002	4.70E-13
rs663129*	18	57838401	60171168	RNU4-17P,MC4R	A	G	0.254	0.010	0.001	7.80E-20	0.254	0.008	0.002	2.54E-04	0.253	0.011	0.002	9.89E-12	0.251	0.012	0.002	1.87E-09	0.233	0.035	0.002	1.20E-55	0.233	0.038	0.002	5.00E-61
rs7132908*	12	50263148	49869365	FAIM2	A	G	0.398	0.008	0.001	7.33E-18	0.396	0.009	0.002	8.39E-06	0.398	0.008	0.001	2.18E-09	0.402	0.008	0.002	2.20E-06	0.384	0.031	0.002	1.80E-56	0.384	0.019	0.002	8.20E-21
rs7525548*	1	75001474	74535790	TNNI3K,FPGT-TNNI3K	T	A	0.567	-0.008	0.001	4.00E-19	0.564	-0.009	0.002	2.86E-06	0.567	-0.012	0.001	1.96E-18	0.568	-0.003	0.002	7.54E-02	0.563	-0.032	0.002	2.20E-58	0.563	-0.013	0.002	4.00E-11
rs141224959*	2	380055	380055	LINC01865	A	G	0.028	-0.024	0.003	1.67E-16	0.028	-0.018	0.006	1.93E-03	0.027	-0.025	0.004	8.23E-10	0.029	-0.026	0.005	1.77E-07	0.038	-0.067	0.005	1.30E-40	0.038	-0.047	0.005	6.90E-20
rs11030377*	11	28576651	28555104	METTL15	G	A	0.498	-0.006	0.001	6.57E-11	0.503	0.005	0.002	1.02E-02	0.503	0.006	0.001	2.60E-06	0.500	0.007	0.002	4.64E-05	0.502	0.009	0.002	9.50E-07	0.502	0.005	0.002	6.60E-03
rs10182181*	2	25150296	24927427	ADCY3,DNAJC27	G	A	0.489	0.010	0.001	1.15E-61	0.494	0.012	0.002	1.24E-10	0.949	0.010	0.001	1.06E-13	0.487	0.009	0.002	2.22E-07	0.486	0.040	0.002	1.10E-96	0.486	0.024	0.002	2.10E-34
rs2094510	1	177843479	177874344	SEC16B	T	C	0.227	-0.013	0.001	2.78E-30	0.227	-0.014	0.002	7.01E-10	0.229	-0.011	0.002	1.31E-11	0.222	-0.012	0.002	2.79E-09	0.790	-0.048	0.002	3.40E-92	0.790	-0.032	0.002	3.60E-40
rs539515	1	177889025	177919890	LINC01741, SEC16B	C	A	0.223	0.014	0.001	1.87E-34	0.224	0.012	0.002	2.90E-08	0.225	0.012	0.002	1.04E-14	0.217	0.013	0.002	4.20E-12	0.205	0.055	0.002	3.60E-116	0.205	0.038	0.002	9.60E-54
rs77165542	2	430975	430975	LINC01865, LINC01874	T	C	0.025	0.032	0.003	4.09E-25	0.025	0.021	0.006	5.95E-04	0.025	-0.033	0.004	1.64E-14	0.027	0.035	0.005	9.74E-12	0.036	-0.090	0.005	1.50E-66	0.036	-0.068	0.005	3.00E-37
rs688671	18	57867526	60200293	RNU4-17P,MC4R	G	A	0.287	0.009	0.001	2.70E-19	0.287	0.007	0.002	1.28E-03	0.287	0.011	0.002	1.18E-14	0.285	0.010	0.002	8.51E-08	0.269	0.031	0.002	1.40E-46	0.269	0.035	0.002	1.20E-56
rs5017302	2	631069	631069	LINC01875, TMEM18	A	G	0.832	-0.013	0.001	2.95E-26	0.832	-0.010	0.003	5.36E-05	0.834	0.015	0.002	1.26E-16	0.830	-0.014	0.002	4.08E-10	0.824	0.044	0.003	2.10E-66	0.824	0.036	0.003	4.30E-44
rs55872725	16	53809123	53775211	FTO	T	C	0.413	-0.016	0.001	4.11E-61	0.413	-0.014	0.002	2.61E-14	0.415	0.016	0.001	6.84E-32	0.410	-0.015	0.002	5.97E-19	0.402	0.048	0.002	6.40E-133	0.402	0.045	0.002	4.50E-113
rs7553158	1	75005238	74539554	TNNI3K,FPGT-TNNI3K	A	G	0.567	0.008	0.001	4.19E-19	0.564	0.009	0.002	2.76E-06	0.567	-0.012	0.001	1.73E-18	0.568	0.003	0.002	7.63E-02	0.563	-0.032	0.002	3.40E-58	0.563	-0.013	0.002	3.40E-11
rs12507026	4	45181334	45179317	THAP12P9, PRDX4P1	T	A	0.415	0.006	0.001	8.84E-11	0.416	0.004	0.002	6.21E-02	0.414	0.008	0.001	9.26E-09	0.419	0.006	0.002	1.94E-04	0.433	0.023	0.002	3.00E-32	0.433	0.017	0.002	2.90E-17

rs4633164	8	143334191	142252830	TSNARE1	T	C	0.397	-0.006	0.001	4.93E-09	0.398	-0.003	0.002	1.50E-01	0.398	0.008	0.001	2.17E-08	0.394	-0.003	0.002	7.79E-02	0.395	0.003	0.002	1.00E-01	0.395	0.008	0.002	5.10E-05
rs62048402	16	53803223	53769311	FTO	A	G	0.412	-0.016	0.001	2.26E-61	0.413	-0.014	0.002	2.07E-14	0.415	-0.016	0.001	1.23E-31	0.409	0.015	0.002	2.89E-19	0.402	0.048	0.002	2.00E-133	0.402	0.045	0.002	1.70E-113
rs10188334	2	653874	653874	LINC01875, TMEM18	T	C	0.166	0.013	0.001	2.85E-25	0.167	0.010	0.003	6.23E-05	0.165	0.014	0.002	5.73E-15	0.169	-0.014	0.002	7.53E-11	0.172	-0.044	0.003	1.10E-65	0.172	-0.036	0.003	3.80E-43
rs509325	1	177894591	177925456	SEC16B	G	T	0.225	0.014	0.001	8.21E-35	0.226	0.013	0.002	1.78E-08	0.227	0.012	0.002	1.73E-14	0.219	0.014	0.002	4.18E-12	0.205	0.055	0.002	7.40E-116	0.205	0.037	0.002	6.60E-53
rs571312	18	57839769	60172536	RNU4-17P,MC4R	A	C	0.254	-0.010	0.001	8.74E-20	0.254	-0.008	0.002	2.54E-04	0.253	-0.011	0.002	1.04E-11	0.251	0.012	0.002	1.74E-09	0.233	0.035	0.002	3.00E-55	0.233	0.038	0.002	4.00E-61

Table S2. Heterogeneity Statistics for SNP-Exposure Associations in the Meta-Analysed GWAS of Body Mass Index Between Menarche and <40 Years. Heterogeneity statistics for SNP-exposure associations across cohorts in the meta-analysed genome-wide association study (GWAS) of body mass index (BMI) in nulliparous women between menarche and <40 years.

SNP - single nucleotide polymorphism identifier, HetChiSq - Cochran's Q statistic for heterogeneity in SNP-exposure associations across cohorts, HetPVal - unadjusted P-value from Cochran's Q test, QBonf - Bonferroni-adjusted P-value to account for multiple testing across variants.

SNP	Chromosome	Base position (build hg19)	Base position (build hg38)	Closest gene	Effect allele	Other allele	HetChiSq	HetPVal	QBonf
rs62033406	16	53824226	53790314	FTO	G	A	10.92	0.03	0.85
rs7707628	5	153546900	154167340	MFAP3	C	T	2.71	0.61	1.00
rs60186497	14	30479220	30010014	PRKD1	T	C	2.33	0.68	1.00
rs2601781	16	4017392	3967391	ADCY9	A	T	3.18	0.53	1.00
rs13130484	4	45175691	45173674	THAP12P9; PRDX4P1	T	C	4.04	0.40	1.00
rs6098816	20	54385642	55810586	CBLN4	A	C	3.57	0.31	1.00
rs28693508	9	120361418	117599140	TLR4	G	C	1.89	0.76	1.00
rs62155011	2	105565719	104949261	MRPS9	C	T	1.06	0.79	1.00
rs574367	1	177873210	177904075	LINC01741, SEC16B	T	G	4.13	0.39	1.00
rs59086897	2	25145173	24922304	ADCY3,DNAJC27	A	T	5.68	0.22	1.00
rs10191896	2	213421764	212557040	ERBB4	T	C	2.47	0.48	1.00
rs11615823	12	26815139	26662206	BHLHE41	C	T	7.66	0.11	1.00
rs2764261	6	108927842	108606639	FOXO3	G	A	2.95	0.57	1.00
rs7613360	3	49916710	49879277	MST1R,ACTL11P	T	C	1.81	0.77	1.00
rs13387091	2	650980	650980	LINC01875,TMEM18	A	G	1.73	0.79	1.00
rs6494678	15	67946247	67653909	MAP2K5	T	C	4.04	0.40	1.00

rs114670539	2	207064335	206199611	CMKLR2	T	C	2.50	0.64	1.00
rs7442885	5	87682877	88387060	TMEM161B-DT	G	C	2.06	0.73	1.00
rs7462788	8	143335170	142253809	PTK2	T	G	0.11	1.00	1.00
rs10840050	11	8473707	8452160	STK33	A	G	5.19	0.27	1.00
rs2180454	14	29690513	29221307	LINC02327	C	T	6.60	0.16	1.00
rs3115665	6	31589264	31621487	PRRC2A	C	G	2.09	0.35	1.00
rs815339	1	190116575	190147445	BRINP3	A	T	0.29	0.99	1.00
rs2076308	6	50791640	50823927	TFAP2B	C	G	2.28	0.68	1.00
rs1902666	10	88011779	86252022	GRID1	T	C	1.55	0.82	1.00
rs3810291	19	47569003	47065746	ZC3H4	A	G	4.50	0.21	1.00
rs663129	18	57838401	60171168	RNU4-17P,MC4R	A	G	7.41	0.12	1.00
rs7132908	12	50263148	49869365	FAIM2	A	G	5.41	0.25	1.00
rs7525548	1	75001474	74535790	TNNI3K,FPGT-TNNI3K	T	A	6.86	0.14	1.00
rs141224959	2	380055	380055	LINC01865	A	G	4.65	0.33	1.00
rs11030377	11	28576651	28555104	METTL15	G	A	3.25	0.52	1.00

Table S3. Genetic Correlation Between Childhood Body Size and Life-Stage–Specific GWAS of Body Mass Index. Genetic correlation coefficients between childhood body size (used as a proxy for BMI) and the four genome-wide association studies (GWAS) of body mass index (BMI) in nulliparous women: between menarche and <40 years, menarche and <20 years, 20 and <30 years, and 30 and <40 years.

rG – genetic correlation, SE – standard error of rG, P - corresponding p-value

Phenotype A (age group or life stage and sample)	Phenotype B (age group or life stage and sample)	Sample size in Phenotype A	Sample size in Phenotype B	Total sample size	rG	SE	P
Prepubertal body size (UKB)	BMI in nulliparous women between menarche and <40 years (meta-analysis)	246511	56628	303139	0.76	0.02	2.13E-223
Prepubertal body size (UKB)	BMI in nulliparous women between menarche and <20 years (meta-analysis)	246511	11396	257907	0.88	0.07	3.10E-41
Prepubertal body size (UKB)	BMI in nulliparous women between 20 and <30 years (meta-analysis)	246511	30272	276783	0.74	0.03	2.39E-121
Prepubertal body size (UKB)	BMI in nulliparous women between 30 and <40 years (meta-analysis)	246511	16565	263076	0.71	0.05	1.67E-47
Prepubertal body size (UKB)	Later life adult body size (UKB)	246511	246511	493022	0.50	0.02	1.92E-101
Later life adult body size (UKB)	BMI in nulliparous women between menarche and 40 years (meta-analysis)	246511	56628	303139	0.85	0.02	0.00E+00
Later life adult body size (UKB)	BMI in nulliparous women between menarche and <20 years (meta-analysis)	246511	11396	257907	0.68	0.06	4.99E-33
Later life adult body size (UKB)	BMI in nulliparous women between 20 and <30 years (meta-analysis)	246511	30272	276783	0.83	0.03	5.35E-190
Later life adult body size (UKB)	BMI in nulliparous women between 30 and <40 years (meta-analysis)	246511	16565	263076	0.95	0.05	6.41E-75
BMI in nulliparous women between menarche and <20 years (meta-analysis)	BMI in nulliparous women between 20 and <30 years (meta-analysis)	11396	30272	41668	0.81	0.07	2.10E-30
BMI in nulliparous women between menarche and <20 years (meta-analysis)	BMI in nulliparous women between 30 and <40 years (meta-analysis)	11396	1656500	1667896	0.88	0.10	1.13E-17
BMI in nulliparous women between menarche and <20 years (meta-analysis)	BMI in nulliparous women between menarche and <40 years (meta-analysis)	11396	56628	68024	0.99	0.05	2.59E-76

BMI in nulliparous women between 20 and <30 years (meta-analysis)	BMI in nulliparous women between 30 and <40 years (meta-analysis)	30272	16565	46837	0.99	0.06	2.24E-53
BMI in nulliparous women between 20 and <30 years (meta-analysis)	BMI in nulliparous women between menarche and <40 years (meta-analysis)	30272	56628	86900	0.97	0.01	0.00E+00
BMI in nulliparous women between 30 and <40 years (meta-analysis)	BMI in nulliparous women between menarche and <40 years (meta-analysis)	16565	56628	73193	1.30	0.05	8.28E-159

Table S4. Univariable and Multivariable Mendelian Randomization Analyses of Body Mass Index on Breast Cancer Risk. Univariable and multivariable Mendelian randomization (MR) analyses for body mass index (BMI) in nulliparous women between menarche and <40 years on overall breast cancer risk and breast cancer subtypes.

nSNP - number of single nucleotide polymorphism identifiers, beta - effect estimate per 1 SD higher log-transformed BMI on overall or subtype-specific breast cancer. SE- standard error of the effect estimate, LCI - lower confidence interval, UCI - upper confidence interval, P - corresponding p-value, IVW - inverse variance weighted, MR - Mendelian randomization

Exposure	Accounting for	Outcome	F-statistic	nSNP	beta	SE	P	Odds ratio	LCI	UCI	Method	MR
BMI in nulliparous women between menarche and <40 years	--	Overall breast cancer	63.7	22	-0.27	0.06	1.27E-05	0.76	0.67	0.86	IVW	Univariable
BMI in nulliparous women between menarche and <40 years	--	Overall breast cancer	63.7	22	-0.33	0.05	9.95E-11	0.72	0.65	0.79	Weighted median	Univariable
BMI in nulliparous women between menarche and <40 years	--	Overall breast cancer	63.7	22	-0.66	0.14	1.31E-04	0.52	0.39	0.68	MR Egger	Univariable
BMI in nulliparous women between menarche and <20 years	--	Overall breast cancer	48.4	3	-0.41	0.06	7.40E-13	0.66	0.59	0.74	IVW	Univariable
BMI in nulliparous women between menarche and <20 years	--	Overall breast cancer	48.4	3	-0.42	0.06	3.84E-11	0.66	0.58	0.75	Weighted median	Univariable
BMI in nulliparous women between menarche and <20 years	--	Overall breast cancer	48.4	3	-0.72	0.69	4.85E-01	0.48	0.12	1.88	MR Egger	Univariable
BMI in nulliparous women between 20 and <30 years	--	Overall breast cancer	59.2	11	-0.32	0.08	4.31E-05	0.73	0.62	0.85	IVW	Univariable
BMI in nulliparous women between 20 and <30 years	--	Overall breast cancer	59.2	11	-0.33	0.06	1.82E-07	0.72	0.63	0.81	Weighted median	Univariable
BMI in nulliparous women between 20 and <30 years	--	Overall breast cancer	59.2	11	-0.47	0.27	1.18E-01	0.63	0.37	1.06	MR Egger	Univariable
BMI in nulliparous women between 30 and <40 years	--	Overall breast cancer	49.2	5	-0.37	0.10	1.70E-04	0.69	0.57	0.84	IVW	Univariable
BMI in nulliparous women between 30 and <40 years	--	Overall breast cancer	49.2	5	-0.34	0.07	1.10E-06	0.71	0.62	0.82	Weighted median	Univariable
BMI in nulliparous women between 30 and <40 years	--	Overall breast cancer	49.2	5	0.02	0.38	9.62E-01	1.02	0.48	2.15	MR Egger	Univariable
BMI in nulliparous women between menarche and <40 years	Prepubertal body size	Overall breast cancer	2.9	16	-0.13	0.06	3.13E-02	0.88	0.79	0.99	IVW	Multivariable

Prepubertal body size	BMI in nulliparous women between menarche and <40 years	Overall breast cancer	3.6	79	-0.15	0.08	4.92E-02	0.86	0.74	1.00	IVW	Multivariable
BMI in nulliparous women between menarche and <40 years	Adult body size	Overall breast cancer	3.6	18	-0.27	0.06	1.77E-06	0.76	0.68	0.85	IVW	Multivariable
Adult body size	BMI in nulliparous women between menarche and <40 years	Overall breast cancer	4.9	115	0.06	0.07	3.93E-01	1.07	0.92	1.23	IVW	Multivariable
BMI in nulliparous women between menarche and <20 years	Prepubertal body size	Overall breast cancer	1.2	3	-0.05	0.05	3.31E-01	0.95	0.86	1.05	IVW	Multivariable
Prepubertal body size	BMI in nulliparous women between menarche and <20 years	Overall breast cancer	1.9	73	-0.24	0.08	1.43E-03	0.79	0.68	0.91	IVW	Multivariable
BMI in nulliparous women between menarche and <20 years	Adult body size	Overall breast cancer	1.8	3	-0.17	0.05	3.09E-04	0.84	0.77	0.92	IVW	Multivariable
Adult body size	BMI in nulliparous women between menarche and <20 years	Overall breast cancer	3.9	114	-0.08	0.06	2.36E-01	0.93	0.82	1.05	IVW	Multivariable
BMI in nulliparous women between 20 and <30 years	Prepubertal body size	Overall breast cancer	2.1	10	-0.06	0.06	3.64E-01	0.95	0.84	1.07	IVW	Multivariable
Prepubertal body size	BMI in nulliparous women between 20 and <30 years	Overall breast cancer	2.8	77	-0.24	0.08	2.31E-03	0.79	0.67	0.92	IVW	Multivariable
BMI in nulliparous women between 20 and <30 years	Adult body size	Overall breast cancer	2.1	10	-0.20	0.06	1.65E-03	0.82	0.73	0.93	IVW	Multivariable
Adult body size	BMI in nulliparous women between 20 and <30 years	Overall breast cancer	3.2	116	-0.03	0.08	7.40E-01	0.97	0.83	1.14	IVW	Multivariable
BMI in nulliparous women between 30 and <40 years	Prepubertal body size	Overall breast cancer	1.7	3	-0.02	0.05	6.32E-01	0.98	0.90	1.07	IVW	Multivariable
Prepubertal body size	BMI in nulliparous women between 30 and <40 years	Overall breast cancer	3.3	75	-0.27	0.07	3.45E-05	0.76	0.67	0.87	IVW	Multivariable
BMI in nulliparous women between 30 and <40 years	Adult body size	Overall breast cancer	1.8	3	-0.18	0.05	2.62E-04	0.84	0.76	0.92	IVW	Multivariable
Adult body size	BMI in nulliparous women between 30 and <40 years	Overall breast cancer	4.5	114	-0.07	0.07	3.17E-01	0.94	0.82	1.06	IVW	Multivariable
BMI in nulliparous women between menarche and <40 years	--	ER+ breast cancer	63.7	22	-0.27	0.06	5.18E-06	0.76	0.68	0.86	IVW	Univariable
BMI in nulliparous women between menarche and <40 years	--	ER+ breast cancer	63.7	22	-0.24	0.06	7.41E-05	0.79	0.70	0.89	Weighted median	Univariable

BMI in nulliparous women between menarche and <40 years	--	ER+ breast cancer	63.7	22	-0.56	0.14	8.42E-04	0.57	0.43	0.75	MR Egger	Univariable
BMI in nulliparous women between menarche and <20 years	--	ER+ breast cancer	48.4	3	-0.40	0.06	1.66E-11	0.67	0.60	0.75	IVW	Univariable
BMI in nulliparous women between menarche and <20 years	--	ER+ breast cancer	48.4	3	-0.41	0.07	8.71E-09	0.66	0.57	0.76	Weighted median	Univariable
BMI in nulliparous women between menarche and <20 years	--	ER+ breast cancer	48.4	3	-0.69	0.73	5.18E-01	0.50	0.12	2.09	MR Egger	Univariable
BMI in nulliparous women between 20 and <30 years	--	ER+ breast cancer	58.3	11	-0.33	0.07	5.61E-06	0.72	0.62	0.83	IVW	Univariable
BMI in nulliparous women between 20 and <30 years	--	ER+ breast cancer	58.3	11	-0.31	0.06	3.70E-07	0.73	0.65	0.83	Weighted median	Univariable
BMI in nulliparous women between 20 and <30 years	--	ER+ breast cancer	58.3	11	-0.50	0.25	7.73E-02	0.61	0.37	0.99	MR Egger	Univariable
BMI in nulliparous women between 30 and <40 years	--	ER+ breast cancer	49.2	5	-0.36	0.09	3.64E-05	0.70	0.59	0.83	IVW	Univariable
BMI in nulliparous women between 30 and <40 years	--	ER+ breast cancer	49.2	5	-0.28	0.07	1.26E-04	0.75	0.65	0.87	Weighted median	Univariable
BMI in nulliparous women between 30 and <40 years	--	ER+ breast cancer	49.2	5	-0.29	0.40	5.16E-01	0.75	0.34	1.63	MR Egger	Univariable
BMI in nulliparous women between menarche and <40 years	Prepubertal body size	ER+ breast cancer	2.9	16	-0.14	0.06	2.54E-02	0.87	0.76	0.98	IVW	Multivariable
Prepubertal body size	BMI in nulliparous women between menarche and <40 years	ER+ breast cancer	3.6	79	-0.09	0.08	2.72E-01	0.91	0.77	1.08	IVW	Multivariable
BMI in nulliparous women between menarche and <40 years	Adult body size	ER+ breast cancer	3.7	18	-0.19	0.04	5.09E-06	0.82	0.76	0.90	IVW	Multivariable
Adult body size	BMI in nulliparous women between menarche and <40 years	ER+ breast cancer	5.6	114	0.08	0.08	3.27E-01	1.09	0.92	1.28	IVW	Multivariable
BMI in nulliparous women between menarche and <20 years	Prepubertal body size	ER+ breast cancer	1.2	3	-0.06	0.06	2.82E-01	0.94	0.84	1.05	IVW	Multivariable
Prepubertal body size	BMI in nulliparous women between menarche and <20 years	ER+ breast cancer	1.9	73	-0.18	0.08	3.19E-02	0.84	0.71	0.98	IVW	Multivariable
BMI in nulliparous women between menarche and <20 years	Adult body size	ER+ breast cancer	1.8	3	-0.19	0.05	3.77E-04	0.83	0.74	0.92	IVW	Multivariable

Adult body size	BMI in nulliparous women between menarche and <20 years	ER+ breast cancer	3.9	113	-0.05	0.07	4.61E-01	0.95	0.82	1.09	IVW	Multivariable
BMI in nulliparous women between 20 and <30 years	Prepubertal body size	ER+ breast cancer	2.2	10	-0.04	0.07	5.49E-01	0.96	0.84	1.09	IVW	Multivariable
Prepubertal body size	BMI in nulliparous women between 20 and <30 years	ER+ breast cancer	4.2	77	-0.21	0.09	1.32E-02	0.81	0.68	0.96	IVW	Multivariable
BMI in nulliparous women between 20 and <30 years	Adult body size	ER+ breast cancer	2.2	10	-0.21	0.07	2.66E-03	0.81	0.70	0.93	IVW	Multivariable
Adult body size	BMI in nulliparous women between 20 and <30 years	ER+ breast cancer	4.5	115	-0.01	0.09	9.10E-01	0.99	0.83	1.19	IVW	Multivariable
BMI in nulliparous women between 30 and <40 years	Prepubertal body size	ER+ breast cancer	1.7	3	-0.02	0.05	6.51E-01	0.98	0.89	1.08	IVW	Multivariable
Prepubertal body size	BMI in nulliparous women between 30 and <40 years	ER+ breast cancer	3.3	75	-0.22	0.07	2.33E-03	0.80	0.69	0.92	IVW	Multivariable
BMI in nulliparous women between 30 and <40 years	Adult body size	ER+ breast cancer	1.8	3	-0.21	0.05	7.92E-05	0.81	0.73	0.90	IVW	Multivariable
Adult body size	BMI in nulliparous women between 30 and <40 years	ER+ breast cancer	4.4	113	-0.03	0.07	7.21E-01	0.97	0.85	1.12	IVW	Multivariable
BMI in nulliparous women between menarche and <40 years	--	ER- breast cancer	63.7	22	-0.42	0.10	1.59E-05	0.66	0.55	0.80	IVW	Univariable
BMI in nulliparous women between menarche and <40 years	--	ER- breast cancer	63.7	22	-0.37	0.10	3.55E-04	0.69	0.56	0.85	Weighted median	Univariable
BMI in nulliparous women between menarche and <40 years	--	ER- breast cancer	63.7	22	-0.73	0.25	7.74E-03	0.48	0.30	0.78	MR Egger	Univariable
BMI in nulliparous women between menarche and <20 years	--	ER- breast cancer	48.4	3	-0.54	0.14	8.30E-05	0.58	0.45	0.76	IVW	Univariable
BMI in nulliparous women between menarche and <20 years	--	ER- breast cancer	48.4	3	-0.64	0.11	2.17E-09	0.53	0.43	0.65	Weighted median	Univariable
BMI in nulliparous women between menarche and <20 years	--	ER- breast cancer	48.4	3	-0.74	1.80	7.54E-01	0.48	0.01	16.46	MR Egger	Univariable
BMI in nulliparous women between 20 and <30 years	--	ER- breast cancer	58.3	11	-0.50	0.10	1.33E-07	0.60	0.50	0.73	IVW	Univariable
BMI in nulliparous women between 20 and <30 years	--	ER- breast cancer	58.3	11	-0.43	0.10	3.43E-05	0.65	0.53	0.80	Weighted median	Univariable

BMI in nulliparous women between 20 and <30 years	--	ER- breast cancer	58.3	11	-0.57	0.34	1.24E-01	0.56	0.29	1.09	MR Egger	Univariable
BMI in nulliparous women between 30 and <40 years	--	ER- breast cancer	49.2	5	-0.48	0.15	1.79E-03	0.62	0.46	0.84	IVW	Univariable
BMI in nulliparous women between 30 and <40 years	--	ER- breast cancer	49.2	5	-0.33	0.14	1.65E-02	0.72	0.55	0.94	Weighted median	Univariable
BMI in nulliparous women between 30 and <40 years	--	ER- breast cancer	49.2	5	-0.08	0.67	9.11E-01	0.92	0.25	3.40	MR Egger	Univariable
BMI in nulliparous women between menarche and <40 years	Prepubertal body size	ER- breast cancer	3.0	16	-0.15	0.09	8.05E-02	0.86	0.72	1.02	IVW	Multivariable
Prepubertal body size	BMI in nulliparous women between menarche and <40 years	ER- breast cancer	4.4	79	-0.21	0.11	6.97E-02	0.81	0.65	1.02	IVW	Multivariable
BMI in nulliparous women between menarche and <40 years	Adult body size	ER- breast cancer	3.7	18	-0.24	0.06	4.24E-05	0.79	0.70	0.88	IVW	Multivariable
Adult body size	BMI in nulliparous women between menarche and <40 years	ER- breast cancer	5.6	114	0.15	0.11	1.87E-01	1.16	0.93	1.45	IVW	Multivariable
BMI in nulliparous women between menarche and <20 years	Prepubertal body size	ER- breast cancer	1.2	3	-0.04	0.08	5.77E-01	0.96	0.82	1.12	IVW	Multivariable
Prepubertal body size	BMI in nulliparous women between menarche and <20 years	ER- breast cancer	1.9	73	-0.32	0.11	4.82E-03	0.72	0.58	0.91	IVW	Multivariable
BMI in nulliparous women between menarche and <20 years	Adult body size	ER- breast cancer	1.8	3	-0.27	0.07	2.92E-04	0.77	0.66	0.89	IVW	Multivariable
Adult body size	BMI in nulliparous women between menarche and <20 years	ER- breast cancer	3.9	113	0.03	0.10	7.53E-01	1.03	0.85	1.25	IVW	Multivariable
BMI in nulliparous women between 20 and <30 years	Prepubertal body size	ER- breast cancer	2.2	10	-0.17	0.09	5.79E-02	0.84	0.70	1.01	IVW	Multivariable
Prepubertal body size	BMI in nulliparous women between 20 and <30 years	ER- breast cancer	4.2	77	-0.21	0.12	8.44E-02	0.81	0.65	1.03	IVW	Multivariable
BMI in nulliparous women between 20 and <30 years	Adult body size	ER- breast cancer	2.2	10	-0.38	0.09	5.93E-05	0.68	0.57	0.82	IVW	Multivariable
Adult body size	BMI in nulliparous women between 20 and <30 years	ER- breast cancer	4.5	115	0.18	0.12	1.58E-01	1.19	0.93	1.52	IVW	Multivariable
BMI in nulliparous women between 30 and <40 years	Prepubertal body size	ER- breast cancer	1.7	3	0.04	0.07	5.85E-01	1.04	0.91	1.19	IVW	Multivariable

Prepubertal body size	BMI in nulliparous women between 30 and <40 years	ER- breast cancer	3.3	75	-0.42	0.10	4.26E-05	0.66	0.54	0.81	IVW	Multivariable
BMI in nulliparous women between 30 and <40 years	Adult body size	ER- breast cancer	1.8	3	-0.15	0.08	4.87E-02	0.86	0.74	1.00	IVW	Multivariable
Adult body size	BMI in nulliparous women between 30 and <40 years	ER- breast cancer	4.4	113	-0.07	0.11	5.14E-01	0.93	0.76	1.15	IVW	Multivariable
BMI in nulliparous women between menarche and <40 years	--	Luminal A-like tumours	63.7	22	-0.24	0.07	2.92E-04	0.79	0.69	0.90	IVW	Univariable
BMI in nulliparous women between menarche and <40 years	--	Luminal A-like tumours	63.7	22	-0.22	0.07	5.80E-04	0.80	0.70	0.91	Weighted median	Univariable
BMI in nulliparous women between menarche and <40 years	--	Luminal A-like tumours	63.7	22	-0.65	0.14	2.12E-04	0.52	0.40	0.69	MR Egger	Univariable
BMI in nulliparous women between menarche and <20 years	--	Luminal A-like tumours	48.4	3	-0.39	0.07	6.08E-09	0.67	0.59	0.77	IVW	Univariable
BMI in nulliparous women between menarche and <20 years	--	Luminal A-like tumours	48.4	3	-0.39	0.08	4.75E-07	0.68	0.58	0.79	Weighted median	Univariable
BMI in nulliparous women between menarche and <20 years	--	Luminal A-like tumours	48.4	3	-0.90	0.75	4.41E-01	0.41	0.09	1.76	MR Egger	Univariable
BMI in nulliparous women between 20 and <30 years	--	Luminal A-like tumours	59.2	11	-0.27	0.09	1.41E-03	0.76	0.64	0.90	IVW	Univariable
BMI in nulliparous women between 20 and <30 years	--	Luminal A-like tumours	59.2	11	-0.15	0.07	2.43E-02	0.86	0.75	0.98	Weighted median	Univariable
BMI in nulliparous women between 20 and <30 years	--	Luminal A-like tumours	59.2	11	-0.52	0.29	1.06E-01	0.60	0.34	1.05	MR Egger	Univariable
BMI in nulliparous women between 30 and <40 years	--	Luminal A-like tumours	49.2	5	-0.34	0.12	4.10E-03	0.71	0.56	0.90	IVW	Univariable
BMI in nulliparous women between 30 and <40 years	--	Luminal A-like tumours	49.2	5	-0.22	0.08	7.92E-03	0.80	0.68	0.94	Weighted median	Univariable
BMI in nulliparous women between 30 and <40 years	--	Luminal A-like tumours	49.2	5	-0.25	0.53	6.71E-01	0.78	0.27	2.21	MR Egger	Univariable
BMI in nulliparous women between menarche and <40 years	Prepubertal body size	Luminal A-like tumours	2.9	16	-0.07	0.07	3.58E-01	0.94	0.81	1.08	IVW	Multivariable
Prepubertal body size	BMI in nulliparous women between menarche and <40 years	Luminal A-like tumours	3.6	79	-0.16	0.09	9.74E-02	0.86	0.71	1.03	IVW	Multivariable

BMI in nulliparous women between menarche and <40 years	Adult body size	Luminal A-like tumours	3.4	18	-0.28	0.07	8.04E-05	0.76	0.66	0.87	IVW	Multivariable
Adult body size	BMI in nulliparous women between menarche and <40 years	Luminal A-like tumours	4.8	115	0.12	0.09	1.81E-01	1.13	0.94	1.36	IVW	Multivariable
BMI in nulliparous women between menarche and <20 years	Prepubertal body size	Luminal A-like tumours	1.2	3	-0.02	0.06	7.75E-01	0.98	0.87	1.11	IVW	Multivariable
Prepubertal body size	BMI in nulliparous women between menarche and <20 years	Luminal A-like tumours	1.7	73	-0.23	0.09	1.18E-02	0.79	0.66	0.95	IVW	Multivariable
BMI in nulliparous women between menarche and <20 years	Adult body size	Luminal A-like tumours	1.7	3	-0.21	0.06	2.66E-04	0.81	0.72	0.91	IVW	Multivariable
Adult body size	BMI in nulliparous women between menarche and <20 years	Luminal A-like tumours	3.2	114	0.01	0.08	9.15E-01	1.01	0.86	1.18	IVW	Multivariable
BMI in nulliparous women between 20 and <30 years	Prepubertal body size	Luminal A-like tumours	2.1	10	0.04	0.07	6.11E-01	1.04	0.90	1.20	IVW	Multivariable
Prepubertal body size	BMI in nulliparous women between 20 and <30 years	Luminal A-like tumours	2.8	77	-0.28	0.10	3.11E-03	0.75	0.62	0.91	IVW	Multivariable
BMI in nulliparous women between 20 and <30 years	Adult body size	Luminal A-like tumours	2.1	10	-0.18	0.08	1.94E-02	0.83	0.72	0.97	IVW	Multivariable
Adult body size	BMI in nulliparous women between 20 and <30 years	Luminal A-like tumours	3.2	116	0.01	0.10	9.38E-01	1.01	0.83	1.23	IVW	Multivariable
BMI in nulliparous women between 30 and <40 years	Prepubertal body size	Luminal A-like tumours	1.7	3	0.04	0.05	4.13E-01	1.05	0.94	1.16	IVW	Multivariable
Prepubertal body size	BMI in nulliparous women between 30 and <40 years	Luminal A-like tumours	3.2	75	-0.29	0.08	2.98E-04	0.75	0.64	0.88	IVW	Multivariable
BMI in nulliparous women between 30 and <40 years	Adult body size	Luminal A-like tumours	1.8	3	-0.18	0.06	3.15E-03	0.84	0.75	0.94	IVW	Multivariable
Adult body size	BMI in nulliparous women between 30 and <40 years	Luminal A-like tumours	4.3	114	-0.01	0.05	8.59E-01	0.99	0.89	1.09	IVW	Multivariable
BMI in nulliparous women between menarche and <40 years	--	Luminal B-like (HER2+) tumours	63.7	22	-0.22	0.07	1.54E-03	0.80	0.70	0.92	IVW	Univariable
BMI in nulliparous women between menarche and <40 years	--	Luminal B-like (HER2+) tumours	63.7	22	-0.26	0.10	8.88E-03	0.77	0.63	0.94	Weighted median	Univariable
BMI in nulliparous women between menarche and <40 years	--	Luminal B-like (HER2+) tumours	63.7	22	-0.64	0.18	1.80E-03	0.53	0.37	0.75	MR Egger	Univariable

BMI in nulliparous women between menarche and <20 years	--	Luminal B-like (HER2+) tumours	48.4	3	-0.34	0.13	6.22E-03	0.71	0.55	0.91	IVW	Univariable
BMI in nulliparous women between menarche and <20 years	--	Luminal B-like (HER2+) tumours	48.4	3	-0.27	0.12	2.85E-02	0.77	0.60	0.97	Weighted median	Univariable
BMI in nulliparous women between menarche and <20 years	--	Luminal B-like (HER2+) tumours	48.4	3	-1.88	0.91	2.88E-01	0.15	0.03	0.91	MR Egger	Univariable
BMI in nulliparous women between <20 years and 30	--	Luminal B-like (HER2+) tumours	59.2	11	-0.29	0.08	1.25E-04	0.75	0.64	0.87	IVW	Univariable
BMI in nulliparous women between <20 years and 30	--	Luminal B-like (HER2+) tumours	59.2	11	-0.29	0.11	9.21E-03	0.75	0.60	0.93	Weighted median	Univariable
BMI in nulliparous women between <20 years and 30	--	Luminal B-like (HER2+) tumours	59.2	11	-0.61	0.24	3.15E-02	0.54	0.34	0.87	MR Egger	Univariable
BMI in nulliparous women between 30 and <40 years	--	Luminal B-like (HER2+) tumours	49.2	5	-0.37	0.09	2.87E-05	0.69	0.58	0.82	IVW	Univariable
BMI in nulliparous women between 30 and <40 years	--	Luminal B-like (HER2+) tumours	49.2	5	-0.31	0.11	6.73E-03	0.73	0.59	0.92	Weighted median	Univariable
BMI in nulliparous women between 30 and <40 years	--	Luminal B-like (HER2+) tumours	49.2	5	-0.38	0.34	3.41E-01	0.68	0.35	1.32	MR Egger	Univariable
BMI in nulliparous women between menarche and <40 years	Prepubertal body size	Luminal B-like (HER2+) tumours	2.9	16	-0.11	0.09	2.48E-01	0.90	0.74	1.08	IVW	Multivariable
Prepubertal body size	BMI in nulliparous women between menarche and <40 years	Luminal B-like (HER2+) tumours	3.6	79	-0.21	0.12	9.27E-02	0.81	0.64	1.04	IVW	Multivariable
BMI in nulliparous women between menarche and <40 years	Adult body size	Luminal B-like (HER2+) tumours	3.4	18	-0.25	0.10	1.21E-02	0.78	0.64	0.95	IVW	Multivariable
Adult body size	BMI in nulliparous women between menarche and <40 years	Luminal B-like (HER2+) tumours	4.8	115	0.00	0.13	9.88E-01	1.00	0.78	1.30	IVW	Multivariable
BMI in nulliparous women between menarche and <20 years	Prepubertal body size	Luminal B-like (HER2+) tumours	1.2	3	-0.12	0.09	1.61E-01	0.88	0.75	1.05	IVW	Multivariable
Prepubertal body size	BMI in nulliparous women between menarche and <20 years	Luminal B-like (HER2+) tumours	1.7	73	-0.20	0.13	1.14E-01	0.82	0.64	1.05	IVW	Multivariable
BMI in nulliparous women between menarche and <20 years	Adult body size	Luminal B-like (HER2+) tumours	1.7	3	-0.11	0.08	1.83E-01	0.89	0.76	1.05	IVW	Multivariable
Adult body size	BMI in nulliparous women between menarche and <20 years	Luminal B-like (HER2+) tumours	3.2	114	-0.18	0.11	1.13E-01	0.84	0.67	1.04	IVW	Multivariable

BMI in nulliparous women between <20 years and 30	Prepubertal body size	Luminal B-like (HER2+) tumours	2.1	10	-0.09	0.10	3.62E-01	0.91	0.75	1.11	IVW	Multivariable
Prepubertal body size	BMI in nulliparous women between 20 and <30 years	Luminal B-like (HER2+) tumours	2.8	77	-0.24	0.13	6.22E-02	0.79	0.61	1.01	IVW	Multivariable
BMI in nulliparous women between <20 years and 30	Adult body size	Luminal B-like (HER2+) tumours	2.1	10	-0.20	0.11	6.54E-02	0.82	0.67	1.01	IVW	Multivariable
Adult body size	BMI in nulliparous women between 20 and <30 years	Luminal B-like (HER2+) tumours	3.2	116	-0.08	0.14	5.84E-01	0.93	0.70	1.22	IVW	Multivariable
BMI in nulliparous women between 30 and <40 years	Prepubertal body size	Luminal B-like (HER2+) tumours	1.7	3	-0.10	0.07	1.59E-01	0.90	0.78	1.04	IVW	Multivariable
Prepubertal body size	BMI in nulliparous women between 30 and <40 years	Luminal B-like (HER2+) tumours	3.2	75	-0.23	0.11	3.18E-02	0.79	0.64	0.98	IVW	Multivariable
BMI in nulliparous women between 30 and <40 years	Adult body size	Luminal B-like (HER2+) tumours	1.8	3	-0.26	0.08	1.46E-03	0.77	0.65	0.90	IVW	Multivariable
Adult body size	BMI in nulliparous women between 30 and <40 years	Luminal B-like (HER2+) tumours	4.3	114	-0.03	0.11	7.75E-01	0.97	0.78	1.21	IVW	Multivariable
BMI in nulliparous women between menarche and <40 years	--	Luminal B/HER2-negative-like tumours	63.7	22	-0.29	0.12	1.91E-02	0.75	0.59	0.95	IVW	Univariable
BMI in nulliparous women between menarche and <40 years	--	Luminal B/HER2-negative-like tumours	63.7	22	-0.45	0.13	8.94E-04	0.64	0.49	0.83	Weighted median	Univariable
BMI in nulliparous women between menarche and <40 years	--	Luminal B/HER2-negative-like tumours	63.7	22	-0.74	0.31	2.63E-02	0.48	0.26	0.87	MR Egger	Univariable
BMI in nulliparous women between menarche and <20 years	--	Luminal B/HER2-negative-like tumours	48.4	3	-0.60	0.11	1.49E-07	0.55	0.44	0.69	IVW	Univariable
BMI in nulliparous women between menarche and <20 years	--	Luminal B/HER2-negative-like tumours	48.4	3	-0.55	0.15	1.74E-04	0.58	0.43	0.77	Weighted median	Univariable
BMI in nulliparous women between menarche and <20 years	--	Luminal B/HER2-negative-like tumours	48.4	3	-0.52	1.07	7.11E-01	0.59	0.07	4.83	MR Egger	Univariable
BMI in nulliparous women between 20 and <30 years	--	Luminal B/HER2-negative-like tumours	59.2	11	-0.35	0.15	1.67E-02	0.71	0.53	0.94	IVW	Univariable

BMI in nulliparous women between 20 and <30 years	--	Luminal B/HER2-negative-like tumours	59.2	11	-0.42	0.14	3.38E-03	0.66	0.50	0.87	Weighted median	Univariable
BMI in nulliparous women between 20 and <30 years	--	Luminal B/HER2-negative-like tumours	59.2	11	-0.67	0.50	2.08E-01	0.51	0.19	1.35	MR Egger	Univariable
BMI in nulliparous women between 30 and <40 years	--	Luminal B/HER2-negative-like tumours	49.2	5	-0.42	0.16	9.40E-03	0.66	0.48	0.90	IVW	Univariable
BMI in nulliparous women between 30 and <40 years	--	Luminal B/HER2-negative-like tumours	49.2	5	-0.46	0.15	2.29E-03	0.63	0.47	0.85	Weighted median	Univariable
BMI in nulliparous women between 30 and <40 years	--	Luminal B/HER2-negative-like tumours	49.2	5	0.28	0.58	6.65E-01	1.32	0.42	4.13	MR Egger	Univariable
BMI in nulliparous women between menarche and <40 years	Prepubertal body size	Luminal B/HER2-negative-like tumours	2.9	16	-0.29	0.11	7.99E-03	0.75	0.60	0.93	IVW	Multivariable
Prepubertal body size	BMI in nulliparous women between menarche and <40 years	Luminal B/HER2-negative-like tumours	3.6	79	0.07	0.15	6.13E-01	1.08	0.81	1.43	IVW	Multivariable
BMI in nulliparous women between menarche and <40 years	Adult body size	Luminal B/HER2-negative-like tumours	3.4	18	-0.36	0.11	9.49E-04	0.70	0.57	0.87	IVW	Multivariable
Adult body size	BMI in nulliparous women between menarche and <40 years	Luminal B/HER2-negative-like tumours	4.8	115	0.17	0.14	2.37E-01	1.18	0.90	1.55	IVW	Multivariable
BMI in nulliparous women between menarche and <20 years	Prepubertal body size	Luminal B/HER2-negative-like tumours	1.2	3	-0.15	0.10	1.38E-01	0.86	0.71	1.05	IVW	Multivariable
Prepubertal body size	BMI in nulliparous women between menarche and <20 years	Luminal B/HER2-negative-like tumours	1.7	73	-0.07	0.15	6.25E-01	0.93	0.70	1.24	IVW	Multivariable
BMI in nulliparous women between menarche and <20 years	Adult body size	Luminal B/HER2-negative-like tumours	1.7	3	-0.19	0.09	3.38E-02	0.83	0.70	0.99	IVW	Multivariable
Adult body size	BMI in nulliparous women between menarche and <20 years	Luminal B/HER2-negative-like tumours	3.2	114	-0.07	0.12	5.76E-01	0.94	0.74	1.18	IVW	Multivariable

BMI in nulliparous women between 20 and <30 years	Prepubertal body size	Luminal B/HER2-negative-like tumours	2.1	10	-0.15	0.12	2.04E-01	0.86	0.69	1.08	IVW	Multivariable
Prepubertal body size	BMI in nulliparous women between 20 and <30 years	Luminal B/HER2-negative-like tumours	2.8	77	-0.06	0.15	6.68E-01	0.94	0.70	1.26	IVW	Multivariable
BMI in nulliparous women between 20 and <30 years	Adult body size	Luminal B/HER2-negative-like tumours	2.1	10	-0.18	0.12	1.29E-01	0.84	0.67	1.05	IVW	Multivariable
Adult body size	BMI in nulliparous women between 20 and <30 years	Luminal B/HER2-negative-like tumours	3.2	116	-0.03	0.15	8.27E-01	0.97	0.72	1.30	IVW	Multivariable
BMI in nulliparous women between 30 and <40 years	Prepubertal body size	Luminal B/HER2-negative-like tumours	1.7	3	-0.10	0.09	2.36E-01	0.90	0.76	1.07	IVW	Multivariable
Prepubertal body size	BMI in nulliparous women between 30 and <40 years	Luminal B/HER2-negative-like tumours	3.2	75	-0.12	0.13	3.54E-01	0.89	0.69	1.14	IVW	Multivariable
BMI in nulliparous women between 30 and <40 years	Adult body size	Luminal B/HER2-negative-like tumours	1.8	3	-0.22	0.09	1.47E-02	0.81	0.68	0.96	IVW	Multivariable
Adult body size	BMI in nulliparous women between 30 and <40 years	Luminal B/HER2-negative-like tumours	4.3	114	-0.02	0.12	8.37E-01	0.98	0.77	1.23	IVW	Multivariable
BMI in nulliparous women between menarche and <40 years	--	HER2-enriched-like tumours	63.7	22	-0.27	0.16	8.07E-02	0.76	0.56	1.03	IVW	Univariable
BMI in nulliparous women between menarche and <40 years	--	HER2-enriched-like tumours	63.7	22	-0.28	0.19	1.33E-01	0.75	0.52	1.09	Weighted median	Univariable
BMI in nulliparous women between menarche and <40 years	--	HER2-enriched-like tumours	63.7	22	-0.99	0.38	1.56E-02	0.37	0.18	0.77	MR Egger	Univariable
BMI in nulliparous women between menarche and <20 years	--	HER2-enriched-like tumours	48.4	3	-0.58	0.17	4.69E-04	0.56	0.40	0.78	IVW	Univariable
BMI in nulliparous women between menarche and <20 years	--	HER2-enriched-like tumours	48.4	3	-0.58	0.21	5.82E-03	0.56	0.37	0.84	Weighted median	Univariable
BMI in nulliparous women between menarche and <20 years	--	HER2-enriched-like tumours	48.4	3	0.27	1.60	8.95E-01	1.30	0.06	30.08	MR Egger	Univariable

BMI in nulliparous women between <20 years and 30	--	HER2-enriched-like tumours	59.2	11	-0.44	0.17	9.08E-03	0.01	0.46	0.90	IVW	Univariable
BMI in nulliparous women between <20 years and 30	--	HER2-enriched-like tumours	59.2	11	-0.39	0.20	4.42E-02	0.67	0.46	0.99	Weighted median	Univariable
BMI in nulliparous women between <20 years and 30	--	HER2-enriched-like tumours	59.2	11	-0.70	0.59	2.69E-01	0.50	0.16	1.59	MR Egger	Univariable
BMI in nulliparous women between 30 and <40 years	--	HER2-enriched-like tumours	49.2	5	-0.46	0.25	6.32E-02	0.63	0.39	1.03	IVW	Univariable
BMI in nulliparous women between 30 and <40 years	--	HER2-enriched-like tumours	49.2	5	-0.44	0.22	4.26E-02	0.65	0.42	0.99	Weighted median	Univariable
BMI in nulliparous women between 30 and <40 years	--	HER2-enriched-like tumours	49.2	5	0.28	1.04	8.07E-01	1.32	0.17	10.08	MR Egger	Univariable
BMI in nulliparous women between menarche and <40 years	Prepubertal body size	HER2-enriched-like tumours	2.9	16	-0.04	0.16	8.14E-01	0.96	0.71	1.31	IVW	Multivariable
Prepubertal body size	BMI in nulliparous women between menarche and <40 years	HER2-enriched-like tumours	3.6	79	-0.24	0.20	2.37E-01	0.79	0.53	1.17	IVW	Multivariable
BMI in nulliparous women between menarche and <40 years	Adult body size	HER2-enriched-like tumours	3.4	18	-0.26	0.15	7.43E-02	0.77	0.57	1.03	IVW	Multivariable
Adult body size	BMI in nulliparous women between menarche and <40 years	HER2-enriched-like tumours	4.8	115	0.19	0.19	3.22E-01	1.21	0.83	1.77	IVW	Multivariable
BMI in nulliparous women between menarche and <20 years	Prepubertal body size	HER2-enriched-like tumours	1.2	3	0.01	0.14	9.41E-01	1.01	0.77	1.32	IVW	Multivariable
Prepubertal body size	BMI in nulliparous women between menarche and <20 years	HER2-enriched-like tumours	1.7	73	-0.36	0.20	7.16E-02	0.70	0.47	1.03	IVW	Multivariable
BMI in nulliparous women between menarche and <20 years	Adult body size	HER2-enriched-like tumours	1.7	3	-0.25	0.12	3.94E-02	0.78	0.61	0.99	IVW	Multivariable
Adult body size	BMI in nulliparous women between menarche and <20 years	HER2-enriched-like tumours	3.2	114	0.10	0.16	5.30E-01	1.11	0.80	1.53	IVW	Multivariable
BMI in nulliparous women between <20 years and 30	Prepubertal body size	HER2-enriched-like tumours	2.1	10	0.03	0.16	8.57E-01	1.03	0.75	1.42	IVW	Multivariable
Prepubertal body size	BMI in nulliparous women between 20 and <30 years	HER2-enriched-like tumours	2.8	77	-0.33	0.21	1.27E-01	0.72	0.48	1.10	IVW	Multivariable
BMI in nulliparous women between <20 years and 30	Adult body size	HER2-enriched-like tumours	2.1	10	-0.18	0.16	2.72E-01	0.84	0.61	1.15	IVW	Multivariable

Adult body size	BMI in nulliparous women between 20 and <30 years	HER2-enriched-like tumours	3.2	116	0.07	0.21	7.24E-01	1.08	0.72	1.62	IVW	Multivariable
BMI in nulliparous women between 30 and <40 years	Prepubertal body size	HER2-enriched-like tumours	1.7	3	-0.04	0.12	7.64E-01	0.96	0.76	1.22	IVW	Multivariable
Prepubertal body size	BMI in nulliparous women between 30 and <40 years	HER2-enriched-like tumours	3.2	75	-0.28	0.18	1.24E-01	0.76	0.53	1.08	IVW	Multivariable
BMI in nulliparous women between 30 and <40 years	Adult body size	HER2-enriched-like tumours	1.8	3	-0.23	0.12	6.54E-02	0.80	0.63	1.01	IVW	Multivariable
Adult body size	BMI in nulliparous women between 30 and <40 years	HER2-enriched-like tumours	4.3	114	0.10	0.17	5.33E-01	1.11	0.80	1.54	IVW	Multivariable
BMI in nulliparous women between menarche and <40 years	--	Triple-negative tumours	63.7	22	-0.34	0.10	8.31E-04	0.71	0.59	0.87	IVW	Univariable
BMI in nulliparous women between menarche and <40 years	--	Triple-negative tumours	63.7	22	-0.36	0.11	1.44E-03	0.70	0.56	0.87	Weighted median	Univariable
BMI in nulliparous women between menarche and <40 years	--	Triple-negative tumours	63.7	22	-0.83	0.24	2.55E-03	0.44	0.27	0.70	MR Egger	Univariable
BMI in nulliparous women between menarche and <20 years	--	Triple-negative tumours	48.4	3	-0.56	0.21	8.90E-03	0.57	0.38	0.87	IVW	Univariable
BMI in nulliparous women between menarche and <20 years	--	Triple-negative tumours	48.4	3	-0.62	0.15	1.90E-05	0.54	0.40	0.71	Weighted median	Univariable
BMI in nulliparous women between menarche and <20 years	--	Triple-negative tumours	48.4	3	-1.57	2.64	6.59E-01	0.21	0.00	36.90	MR Egger	Univariable
BMI in nulliparous women between 20 and <30 years	--	Triple-negative tumours	59.2	11	-0.41	0.11	1.51E-04	0.66	0.54	0.82	IVW	Univariable
BMI in nulliparous women between 20 and <30 years	--	Triple-negative tumours	59.2	11	-0.34	0.11	2.93E-03	0.71	0.57	0.89	Weighted median	Univariable
BMI in nulliparous women between 20 and <30 years	--	Triple-negative tumours	59.2	11	-0.46	0.38	2.55E-01	0.63	0.30	1.32	MR Egger	Univariable
BMI in nulliparous women between 30 and <40 years	--	Triple-negative tumours	49.2	5	-0.40	0.18	2.55E-02	0.67	0.47	0.95	IVW	Univariable
BMI in nulliparous women between 30 and <40 years	--	Triple-negative tumours	49.2	5	-0.21	0.14	1.32E-01	0.81	0.62	1.07	Weighted median	Univariable
BMI in nulliparous women between 30 and <40 years	--	Triple-negative tumours	49.2	5	-0.08	0.79	9.21E-01	0.92	0.20	4.31	MR Egger	Univariable

BMI in nulliparous women between menarche and <40 years	Prepubertal body size	Triple-negative tumours	2.9	16	-0.14	0.10	1.66E-01	0.87	0.71	1.06	IVW	Multivariable
Prepubertal body size	BMI in nulliparous women between menarche and <40 years	Triple-negative tumours	3.6	79	-0.20	0.14	1.48E-01	0.82	0.63	1.07	IVW	Multivariable
BMI in nulliparous women between menarche and <40 years	Adult body size	Triple-negative tumours	3.4	18	-0.31	0.10	2.45E-03	0.73	0.60	0.90	IVW	Multivariable
Adult body size	BMI in nulliparous women between menarche and <40 years	Triple-negative tumours	4.8	115	0.06	0.14	6.38E-01	1.07	0.82	1.39	IVW	Multivariable
BMI in nulliparous women between menarche and <20 years	Prepubertal body size	Triple-negative tumours	1.2	3	0.02	0.10	8.34E-01	1.02	0.84	1.23	IVW	Multivariable
Prepubertal body size	BMI in nulliparous women between menarche and <20 years	Triple-negative tumours	1.7	73	-0.35	0.14	1.17E-02	0.70	0.53	0.92	IVW	Multivariable
BMI in nulliparous women between menarche and <20 years	Adult body size	Triple-negative tumours	1.7	3	-0.17	0.09	4.86E-02	0.84	0.71	1.00	IVW	Multivariable
Adult body size	BMI in nulliparous women between menarche and <20 years	Triple-negative tumours	3.2	114	-0.09	0.12	4.23E-01	0.91	0.72	1.15	IVW	Multivariable
BMI in nulliparous women between 20 and <30 years	Prepubertal body size	Triple-negative tumours	2.1	10	-0.27	0.11	1.45E-02	0.76	0.61	0.95	IVW	Multivariable
Prepubertal body size	BMI in nulliparous women between 20 and <30 years	Triple-negative tumours	2.8	77	-0.07	0.14	6.41E-01	0.94	0.71	1.24	IVW	Multivariable
BMI in nulliparous women between 20 and <30 years	Adult body size	Triple-negative tumours	2.1	10	-0.36	0.11	1.32E-03	0.70	0.56	0.87	IVW	Multivariable
Adult body size	BMI in nulliparous women between 20 and <30 years	Triple-negative tumours	3.2	116	0.11	0.15	4.53E-01	1.12	0.84	1.48	IVW	Multivariable
BMI in nulliparous women between 30 and <40 years	Prepubertal body size	Triple-negative tumours	1.7	3	-0.01	0.08	8.66E-01	0.99	0.84	1.16	IVW	Multivariable
Prepubertal body size	BMI in nulliparous women between 30 and <40 years	Triple-negative tumours	3.2	75	-0.32	0.12	8.91E-03	0.72	0.57	0.92	IVW	Multivariable
BMI in nulliparous women between 30 and <40 years	Adult body size	Triple-negative tumours	1.8	3	-0.15	0.09	1.00E-01	0.86	0.72	1.03	IVW	Multivariable
Adult body size	BMI in nulliparous women between 30 and <40 years	Triple-negative tumours	4.3	114	-0.12	0.12	3.16E-01	0.89	0.70	1.12	IVW	Multivariable

Table S5. Steiger Filtering Results Testing the Direction of Causality Between Life-Stage Body Mass Index Categories and Breast Cancer Risk. Steiger filtering results testing the direction of causality between body mass index (BMI) in nulliparous women between menarche and <40 years and breast cancer risk.

R^2 (Exposure) - proportion of variance in the exposure explained by the genetic instruments; R^2 (Outcome) - proportion of variance in the outcome explained by the same instruments; Correct causal direction - whether the direction of effect from exposure to outcome is supported; Steiger P value - statistical evidence supporting the inferred causal direction based on variance explained.

Exposure	Outcome	R^2 (Exposure)	R^2 (Outcome)	Correct causal direction	Steiger P value
BMI in nulliparous women between menarche and <40 years	Overall breast cancer	0.106	0.0008	TRUE	<1.00E-200
BMI in nulliparous women between menarche and <20 years	Overall breast cancer	0.029	0.0005	TRUE	3.89E-26
BMI in nulliparous women between 20 and <30 years	Overall breast cancer	0.086	0.0007	TRUE	2.14E-183
BMI in nulliparous women between 30 and <40 years	Overall breast cancer	0.086	0.0006	TRUE	4.32E-86

Table S6. Univariable MR-RAPS Estimates for the Effect of Genetically Predicted Body Mass Index at Different Life Stages on Breast Cancer Risk. Univariable MR-RAPS estimates (instrument selection threshold $P < 1 \times 10^{-5}$) for the effect of genetically predicted body mass index (BMI) in nulliparous women at different life stages on overall breast cancer risk.

nSNP - number of single nucleotide polymorphism identifiers, beta - effect estimate per 1 SD higher log-transformed BMI on overall or subtype-specific breast cancer, SE- standard error of the effect estimate, P - corresponding p-value, , LCI - lower confidence interval, UCI - upper confidence interval, MR-RAPS - MR-RAPS – Mendelian randomization robust adjusted profile score, MR - Mendelian randomization

Exposure	Outcome	nSNP	beta	SE	P	Odds ratio	LCI	UCI	Method	MR
BMI in nulliparous women between menarche and <40 years	Overall breast cancer	129	-0.11	0.02	0.00	0.89	0.87	0.92	MR-RAPS	Univariable
BMI in nulliparous women between menarche and <20 years	Overall breast cancer	34	-0.01	0.02	0.35	0.99	0.95	1.03	MR-RAPS	Univariable
BMI in nulliparous women between 20 and <30 years	Overall breast cancer	77	-0.25	0.04	0.00	0.78	0.73	0.84	MR-RAPS	Univariable
BMI in nulliparous women between 30 and <40 years	Overall breast cancer	61	-0.01	0.01	0.03	0.99	0.97	1.00	MR-RAPS	Univariable

Table S7. Univariable Mendelian Randomization Analyses for the Effect of Parity on Body Mass Index. Univariable Mendelian randomization (MR) analyses assessing the effect of parity on body mass index (BMI) in nulliparous women between menarche and <40 years.

nSNP - number of single nucleotide polymorphism identifiers, beta – effect estate of number of live births on BMI and per 1 SD higher log-transformed BMI on number of life births, SE- standard error of the effect estimate, LCI - lower confidence interval, UCI - upper confidence interval, P - corresponding p-value, IVW - inverse variance weighted

Exposure	Outcome	nSNP	beta	SE	P	Odds ratio	LCI	UCI	Method
Number of live births	BMI in nulliparous women between menarche and <40 years	10	-0.01	0.03	0.58	0.99	0.94	1.04	IVW
Number of live births	BMI in nulliparous women between menarche and <40 years	10	-0.02	0.02	0.36	0.98	0.94	1.02	Weighted median
Number of live births	BMI in nulliparous women between menarche and <40 years	10	0.21	0.24	0.41	1.23	0.77	1.95	MR Egger
Number of live births	BMI in nulliparous women between menarche and <20 years	10	0.01	0.04	0.75	1.01	0.94	1.09	IVW
Number of live births	BMI in nulliparous women between menarche and <20 years	10	-0.03	0.04	0.52	0.97	0.90	1.06	Weighted median
Number of live births	BMI in nulliparous women between menarche and <20 years	10	-0.27	0.34	0.45	0.76	0.39	1.48	MR Egger
Number of live births	BMI in nulliparous women between 20 and <30 years	10	0.00	0.03	0.94	1.00	0.94	1.06	IVW
Number of live births	BMI in nulliparous women between 20 and <30 years	10	-0.01	0.03	0.63	0.99	0.93	1.05	Weighted median
Number of live births	BMI in nulliparous women between 20 and <30 years	10	0.30	0.29	0.32	1.35	0.77	2.37	MR Egger
Number of live births	BMI in nulliparous women between 30 and <40 years	10	-0.07	0.03	0.05	0.93	0.87	1.00	IVW
Number of live births	BMI in nulliparous women between 30 and <40 years	10	-0.07	0.04	0.07	0.94	0.87	1.00	Weighted median
Number of live births	BMI in nulliparous women between 30 and <40 years	10	0.08	0.33	0.81	1.09	0.57	2.07	MR Egger
BMI in nulliparous women between menarche and <40 years	Number of live births	28	0.01	0.02	0.66	1.01	0.96	1.06	IVW

BMI in nulliparous women between menarche and <40 years	Number of live births	28	0.01	0.01	0.29	1.01	0.99	1.02	Weighted median
BMI in nulliparous women between menarche and <40 years	Number of live births	28	-0.02	0.03	0.64	0.98	0.92	1.05	MR Egger
BMI in nulliparous women between menarche and <20 years	Number of live births	3	-0.01	0.02	0.73	0.99	0.95	1.03	IVW
BMI in nulliparous women between menarche and <20 years	Number of live births	3	-0.01	0.01	0.27	0.99	0.97	1.01	Weighted median
BMI in nulliparous women between menarche and <20 years	Number of live births	3	-0.12	0.23	0.69	0.89	0.56	1.40	MR Egger
BMI in nulliparous women between 20 and <30 years	Number of live births	10	0.00	0.02	0.92	1.00	0.96	1.04	IVW
BMI in nulliparous women between 20 and <30 years	Number of live births	10	-0.01	0.01	0.64	0.99	0.97	1.01	Weighted median
BMI in nulliparous women between 20 and <30 years	Number of live births	10	0.00	0.05	0.94	1.00	0.91	1.10	MR Egger
BMI in nulliparous women between 30 and <40 years	Number of live births	5	-0.01	0.02	0.76	0.99	0.96	1.03	IVW
BMI in nulliparous women between 30 and <40 years	Number of live births	5	-0.01	0.01	0.40	0.99	0.96	1.01	Weighted median
BMI in nulliparous women between 30 and <40 years	Number of live births	5	-0.06	0.06	0.44	0.94	0.83	1.07	MR Egger

Table S8. Multivariable Mendelian Randomization Analyses of Body Mass Index on Breast Cancer Risk Accounting for Age at Menarche. Multivariable Mendelian randomization (MR) analyses assessing the effect of body mass index (BMI) in nulliparous women between menarche and <40 years on overall and subtype-specific breast cancer risk, accounting for age at menarche.

nSNP - number of single nucleotide polymorphism identifiers, beta - effect estimate per 1 SD higher log-transformed BMI and increase in age at menarche (years) on overall or subtype-specific breast cancer, SE-standard error of the effect estimate, LCI - lower confidence interval, UCI - upper confidence interval, P - corresponding p-value, IVW - inverse variance weighted, MR - Mendelian randomization

Exposure	Accounting for	Outcome	nSNP	beta	SE	P	Odds ratio	LCI	UCI	Method	MR
BMI in nulliparous women between menarche and <40 years	Age at menarche	Overall breast cancer	13	-0.32	0.05	4.04E-11	0.72	0.66	0.79	IVW	Multivariable
Age at menarche	BMI in nulliparous women between menarche and <40 years	Overall breast cancer	70	-0.05	0.03	1.37E-01	0.95	0.90	1.01	IVW	Multivariable
BMI in nulliparous women between menarche and <20 years	Age at menarche	Overall breast cancer	3	-0.31	0.05	4.56E-09	0.74	0.67	0.81	IVW	Multivariable
Age at menarche	BMI in nulliparous women between menarche and <20 years	Overall breast cancer	68	-0.05	0.03	1.35E-01	0.95	0.90	1.01	IVW	Multivariable
BMI in nulliparous women between 20 and <30 years	Age at menarche	Overall breast cancer	7	-0.25	0.06	2.10E-05	0.78	0.70	0.87	IVW	Multivariable
Age at menarche	BMI in nulliparous women between 20 and <30 years	Overall breast cancer	69	-0.04	0.03	2.08E-01	0.96	0.91	1.02	IVW	Multivariable
BMI in nulliparous women between 30 and <40 years	Age at menarche	Overall breast cancer	5	-0.28	0.06	1.14E-06	0.76	0.68	0.85	IVW	Multivariable
Age at menarche	BMI in nulliparous women between menarche and <40 years	Overall breast cancer	69	-0.05	0.03	1.56E-01	0.95	0.90	1.01	IVW	Multivariable
BMI in nulliparous women between menarche and <40 years	Age at menarche	ER+ breast cancer	13	-0.33	0.06	2.24E-10	0.72	0.64	0.80	IVW	Multivariable
Age at menarche	BMI in nulliparous women between menarche and <40 years	ER+ breast cancer	70	-0.06	0.03	6.47E-02	0.94	0.89	1.00	IVW	Multivariable
BMI in nulliparous women between menarche and <20 years	Age at menarche	ER+ breast cancer	3	-0.32	0.05	8.32E-09	0.73	0.66	0.81	IVW	Multivariable
Age at menarche	BMI in nulliparous women between menarche and <20 years	ER+ breast cancer	68	-0.07	0.03	5.33E-02	0.93	0.88	0.99	IVW	Multivariable

BMI in nulliparous women between 20 and <30 years	Age at menarche	ER+ breast cancer	7	-0.27	0.06	6.14E-06	0.76	0.68	0.85	IVW	Multivariable
Age at menarche	BMI in nulliparous women between 20 and <30 years	ER+ breast cancer	69	-0.06	0.04	9.07E-02	0.94	0.87	1.02	IVW	Multivariable
BMI in nulliparous women between 30 and <40 years	Age at menarche	ER+ breast cancer	5	-0.28	0.06	3.72E-06	0.76	0.68	0.85	IVW	Multivariable
Age at menarche	BMI in nulliparous women between menarche and <40 years	ER+ breast cancer	69	-0.06	0.04	7.58E-02	0.94	0.87	1.02	IVW	Multivariable
BMI in nulliparous women between menarche and <40 years	Age at menarche	ER- breast cancer	13	-0.37	0.08	2.97E-06	0.69	0.59	0.80	IVW	Multivariable
Age at menarche	BMI in nulliparous women between menarche and <40 years	ER- breast cancer	70	0.00	0.05	9.34E-01	1.00	0.91	1.10	IVW	Multivariable
BMI in nulliparous women between menarche and <20 years	Age at menarche	ER- breast cancer	3	-0.36	0.08	3.35E-05	0.70	0.60	0.82	IVW	Multivariable
Age at menarche	BMI in nulliparous women between menarche and <20 years	ER- breast cancer	68	0.01	0.05	9.07E-01	1.01	0.92	1.11	IVW	Multivariable
BMI in nulliparous women between 20 and <30 years	Age at menarche	ER- breast cancer	7	-0.33	0.09	2.41E-04	0.72	0.60	0.86	IVW	Multivariable
Age at menarche	BMI in nulliparous women between 20 and <30 years	ER- breast cancer	69	0.01	0.05	9.20E-01	1.01	0.92	1.11	IVW	Multivariable
BMI in nulliparous women between 30 and <40 years	Age at menarche	ER- breast cancer	5	-0.30	0.10	1.45E-03	0.74	0.61	0.89	IVW	Multivariable
Age at menarche	BMI in nulliparous women between menarche and <40 years	ER- breast cancer	69	0.02	0.06	7.81E-01	1.02	0.91	1.15	IVW	Multivariable
BMI in nulliparous women between menarche and <40 years	Age at menarche	Luminal A-like tumours	13	-0.29	0.06	1.77E-07	0.75	0.67	0.83	IVW	Multivariable
Age at menarche	BMI in nulliparous women between menarche and <40 years	Luminal A-like tumours	70	-0.05	0.04	1.20E-01	0.95	0.88	1.03	IVW	Multivariable
BMI in nulliparous women between menarche and <20 years	Age at menarche	Luminal A-like tumours	3	-0.29	0.06	1.08E-06	0.75	0.67	0.85	IVW	Multivariable
Age at menarche	BMI in nulliparous women between menarche and <20 years	Luminal A-like tumours	68	-0.06	0.04	1.02E-01	0.94	0.87	1.02	IVW	Multivariable
BMI in nulliparous women between 20 and <30 years	Age at menarche	Luminal A-like tumours	7	-0.24	0.07	2.19E-04	0.79	0.69	0.90	IVW	Multivariable

Age at menarche	BMI in nulliparous women between 20 and <30 years	Luminal A-like tumours	69	-0.06	0.04	1.25E-01	0.94	0.87	1.02	IVW	Multivariable
BMI in nulliparous women between 30 and <40 years	Age at menarche	Luminal A-like tumours	5	-0.24	0.06	1.46E-04	0.79	0.69	0.89	IVW	Multivariable
Age at menarche	BMI in nulliparous women between 30 and <40 years	Luminal A-like tumours	69	-0.06	0.04	1.35E-01	0.94	0.87	1.02	IVW	Multivariable
BMI in nulliparous women between menarche and <40 years	Age at menarche	Luminal B-like (HER2+) tumours	13	-0.40	0.09	2.84E-06	0.67	0.56	0.79	IVW	Multivariable
Age at menarche	BMI in nulliparous women between menarche and <40 years	Luminal B-like (HER2+) tumours	70	-0.06	0.05	2.70E-01	0.94	0.85	1.04	IVW	Multivariable
BMI in nulliparous women between menarche and <20 years	Age at menarche	Luminal B-like (HER2+) tumours	3	-0.40	0.09	6.38E-06	0.67	0.56	0.80	IVW	Multivariable
Age at menarche	BMI in nulliparous women between menarche and <20 years	Luminal B-like (HER2+) tumours	68	-0.06	0.05	2.54E-01	0.94	0.85	1.04	IVW	Multivariable
BMI in nulliparous women between 20 and <30 years	Age at menarche	Luminal B-like (HER2+) tumours	7	-0.32	0.09	1.13E-03	0.73	0.60	0.87	IVW	Multivariable
Age at menarche	BMI in nulliparous women between 20 and <30 years	Luminal B-like (HER2+) tumours	69	-0.05	0.06	4.16E-01	0.95	0.85	1.07	IVW	Multivariable
BMI in nulliparous women between 30 and <40 years	Age at menarche	Luminal B-like (HER2+) tumours	5	-0.28	0.10	4.30E-03	0.76	0.63	0.92	IVW	Multivariable
Age at menarche	BMI in nulliparous women between 30 and <40 years	Luminal B-like (HER2+) tumours	69	-0.05	0.06	3.57E-01	0.95	0.85	1.07	IVW	Multivariable
BMI in nulliparous women between menarche and <40 years	Age at menarche	Luminal B/HER2-negative-like tumours	13	-0.32	0.08	4.64E-05	0.73	0.62	0.85	IVW	Multivariable
Age at menarche	BMI in nulliparous women between menarche and <40 years	Luminal B/HER2-negative-like tumours	70	-0.09	0.05	5.86E-02	0.91	0.83	1.01	IVW	Multivariable
BMI in nulliparous women between menarche and <20 years	Age at menarche	Luminal B/HER2-negative-like tumours	3	-0.34	0.08	3.26E-05	0.71	0.61	0.84	IVW	Multivariable
Age at menarche	BMI in nulliparous women between menarche and <20 years	Luminal B/HER2-negative-like tumours	68	-0.10	0.05	4.58E-02	0.90	0.82	1.00	IVW	Multivariable
BMI in nulliparous women between 20 and <30 years	Age at menarche	Luminal B/HER2-negative-like tumours	7	-0.26	0.09	3.94E-03	0.77	0.64	0.92	IVW	Multivariable
Age at menarche	BMI in nulliparous women between 20 and <30 years	Luminal B/HER2-negative-like tumours	69	-0.09	0.05	8.97E-02	0.91	0.83	1.01	IVW	Multivariable

BMI in nulliparous women between 30 and <40 years	Age at menarche	Luminal B/HER2-negative-like tumours	5	-0.36	0.08	2.95E-05	0.70	0.59	0.82	IVW	Multivariable
Age at menarche	BMI in nulliparous women between 30 and <40 years	Luminal B/HER2-negative-like tumours	69	-0.11	0.05	3.58E-02	0.90	0.81	0.99	IVW	Multivariable
BMI in nulliparous women between menarche and <40 years	Age at menarche	HER2-enriched-like tumours	13	-0.19	0.13	1.24E-01	0.83	0.64	1.06	IVW	Multivariable
Age at menarche	BMI in nulliparous women between menarche and <40 years	HER2-enriched-like tumours	70	0.11	0.08	1.63E-01	1.12	0.95	1.31	IVW	Multivariable
BMI in nulliparous women between menarche and <20 years	Age at menarche	HER2-enriched-like tumours	3	-0.27	0.13	3.50E-02	0.76	0.59	0.98	IVW	Multivariable
Age at menarche	BMI in nulliparous women between menarche and <20 years	HER2-enriched-like tumours	68	0.11	0.08	1.98E-01	1.12	0.95	1.31	IVW	Multivariable
BMI in nulliparous women between 20 and <30 years	Age at menarche	HER2-enriched-like tumours	7	-0.16	0.14	2.65E-01	0.85	0.65	1.12	IVW	Multivariable
Age at menarche	BMI in nulliparous women between 20 and <30 years	HER2-enriched-like tumours	69	0.11	0.08	1.91E-01	1.12	0.95	1.31	IVW	Multivariable
BMI in nulliparous women between 30 and <40 years	Age at menarche	HER2-enriched-like tumours	5	-0.28	0.14	4.52E-02	0.75	0.57	0.99	IVW	Multivariable
Age at menarche	BMI in nulliparous women between 30 and <40 years	HER2-enriched-like tumours	69	0.11	0.08	1.77E-01	1.12	0.95	1.31	IVW	Multivariable
BMI in nulliparous women between menarche and <40 years	Age at menarche	Triple-negative tumours	13	-0.31	0.08	2.00E-04	0.73	0.63	0.86	IVW	Multivariable
Age at menarche	BMI in nulliparous women between menarche and <40 years	Triple-negative tumours	70	-0.03	0.05	5.94E-01	0.97	0.88	1.07	IVW	Multivariable
BMI in nulliparous women between menarche and <20 years	Age at menarche	Triple-negative tumours	3	-0.31	0.09	5.09E-04	0.74	0.62	0.88	IVW	Multivariable
Age at menarche	BMI in nulliparous women between menarche and <20 years	Triple-negative tumours	68	-0.03	0.05	6.10E-01	0.97	0.88	1.07	IVW	Multivariable
BMI in nulliparous women between 20 and <30 years	Age at menarche	Triple-negative tumours	7	-0.28	0.09	1.70E-03	0.75	0.63	0.91	IVW	Multivariable
Age at menarche	BMI in nulliparous women between 20 and <30 years	Triple-negative tumours	69	-0.03	0.05	5.96E-01	0.97	0.88	1.07	IVW	Multivariable
BMI in nulliparous women between 30 and <40 years	Age at menarche	Triple-negative tumours	5	-0.24	0.09	7.28E-03	0.78	0.66	0.93	IVW	Multivariable

Age at menarche	BMI in nulliparous women between 30 and <40 years	Triple-negative tumours	69	-0.02	0.05	6.74E-01	0.98	0.89	1.08	IVW	Multivariable

Table S9. STROBE-MR Reporting Checklist for Mendelian Randomization Studies. Strengthening the Reporting of Observational Studies in Epidemiology using Mendelian Randomization (STROBE-MR) checklist of recommended items to address when reporting Mendelian randomization studies (98, 99).

Item No.	Section	Checklist item	Relevant text from manuscript
1	**TITLE and ABSTRACT**	Indicate Mendelian randomization (MR) as the study's design in the title and/or the abstract if that is a main purpose of the study	Abstract:<br><br>Results were meta-analysed, and two-sample univariable and multivariable Mendelian randomisation was applied within a lifecourse framework to assess the causal effect of BMI on breast cancer risk.
	**INTRODUCTION**		
2	**Background**	Explain the scientific background and rationale for the reported study. What is the exposure? Is a potential causal relationship between exposure and outcome plausible? Justify why MR is a helpful method to address the study question	See entire Introduction and specifically:<br><br>We aimed to estimate the effect of higher BMI between menarche and first full-term pregnancy – or throughout early adulthood (<40 years) in individuals who remain nulliparous – on breast cancer risk later in life. This previously understudied period represents a critical window of susceptibility to exposures influencing breast cancer risk. We first conducted genome-wide association studies (GWAS) of BMI in nulliparous women from five population-based cohorts across the full period from menarche to <40 years, and then stratified the analyses into three groups: menarche to <20 years, 20 to <30 years, and 30 to <40 years. This approach allowed us to assess the consistency of genetic variant effects on BMI across early adulthood and to apply these profiles in downstream lifecourse MR analyses. We then assessed these effects on overall breast cancer and different subtypes, including ER status (ER+/ER-) and five molecular subtypes: Luminal A-like, Luminal B-like (HER2-positive), Luminal B (HER2-negative-like), HER2-enriched, and triple-negative breast cancer using data from the Breast Cancer Association Consortium (BCAC) (*28, 29*). While we were not able to assess effects by menopausal status directly, we examined subtype-specific effects as a means of providing preliminary insights given that some evidence indicates that certain subtypes may be more common before verses after menopause (*30-32*).
3	**Objectives**	State specific objectives clearly, including pre-specified causal hypotheses (if any). State that MR is a method that, under specific assumptions, intends to estimate causal effects	See above and the following:<br><br>Mendelian randomization (MR) is a technique that exploits the quasi-random distribution of genetic variants from parents to offspring, independent of the influence from other traits. Under specific assumptions, MR aims to estimate causal effects by reducing susceptibility to confounding factors, including confounding by undiagnosed existing disease and disease processes (reverse causation) (*8, 9*). Recent developments in lifecourse MR methodology include

				multivariable MR (MVMR) (*10-12*). This approach enables the direct estimation of the effects of an exposure measured at a specific life stage, controlling for the same exposure measured at another life stage, on later life outcomes.
	**METHODS**			
4	**Study design and data sources**		Present key elements of the study design early in the article. Consider including a table listing sources of data for all phases of the study. For each data source contributing to the analysis, describe the following:	See entire 'Data sources and study design' section, specifically:<br>This study included five large, population-based prospective cohorts with available genomic and phenotypic BMI data from nulliparous women between menarche and age <40 years: the Avon Longitudinal Study of Parents and Children (ALSPAC), the Trøndelag Health Study (HUNT), the Norwegian Mother, Father and Child Cohort Study (MoBa), Generation R, and Generation Scotland. Participant selection is shown in Figure 1.
		a)	Setting: Describe the study design and the underlying population, if possible. Describe the setting, locations, and relevant dates, including periods of recruitment, exposure, follow-up, and data collection, when available.	See entire 'Data sources and study design' section and Supplementary Text
		b)	Participants: Give the eligibility criteria, and the sources and methods of selection of participants. Report the sample size, and whether any power or sample size calculations were carried out prior to the main analysis	See entire 'Data sources and study design' section and Supplementary Text
		c)	Describe measurement, quality control and selection of genetic variants	See entire 'Materials and methods' section, specifically the 'Genotyping, quality control and imputation in each cohort' section and Supplementary text
		d)	For each exposure, outcome, and other relevant variables, describe methods of assessment and diagnostic criteria for diseases	See entire 'Data sources and study design' section and Supplementary Text
		e)	Provide details of ethics committee approval and participant informed consent, if relevant	Informed consent for cohort participation was obtained from all participants and ethical approval was obtained from the Regional Committee for Medical and Health Research Ethics, Central Norway (REK Central application number 2018/2488) (HUNT), the ALSPAC Ethics and Law Committee and the Local Research Ethics Committees (ALSPAC), and the Medical Ethical Committee of Erasmus MC, University Medical Center Rotterdam, approved the study (MEC 198.782/2001/31) (Generation R), and The Regional Committees for Medical and Health Research Ethics (REK application number 2016/1702) (MoBa). Written consent was obtained from all participants in Generation Scotland. All components of Generation Scotland received ethical approval from the NHS Tayside Committee on Medical Research Ethics (REC Reference Number: 05/S1401/89). Generation Scotland has also been granted Research Tissue Bank status by the East of Scotland Research Ethics Service (REC Reference Number: 20-ES-0021).

5	**Assumptions**	Explicitly state the three core IV assumptions for the main analysis (relevance, independence and exclusion restriction) as well assumptions for any additional or sensitivity analysis	See 'Lifecourse Mendelian randomization (MR) analysis' section, specifically:<br><br>Our primary two-sample MR analyses used the inverse variance weighted (IVW) estimator, implemented in the TwoSampleMR R package (*77*). When genetic variants are used as instrumental variables in MR, the assumptions of instrumental variables must be met, i.e., the genetic variants used must (i) be strongly associated with the exposure of interest ("relevance"), (ii) not share common causes with the outcome ("independence"), and (iii) not affect the outcome other than through the exposure ("exclusion-restriction") (*78*). We conducted sensitivity analyses, which relax the assumptions made about horizontal pleiotropy, including MR Egger regression (*79*), and the weighted median-based estimator (*80*). We also applied Steiger filtering (*81*) to test the direction of causality between BMI between menarche and first birth and breast cancer risk, by assessing whether the genetic instruments explained more variance in the exposure than in the outcome. In addition we conducted analyses using the MR-RAPS method which is robust to weak instruments and systematic pleiotropy (*82*). To allow the inclusion of a larger set of instruments, we applied a liberal SNP selection threshold of $P < 1 \times 10^{-5}$. We ran IVW multivariable MR (MVMR), an extension of MR that employs multiple genetic variants associated with multiple measured risk factors, to calculate the direct and indirect effects of BMI between menarche and first birth on breast cancer outcomes. effects of BMI between menarche and first birth on breast cancer outcomes.
6	**Statistical methods: main analysis**	Describe statistical methods and statistics used	See entire 'Statistical methods' section
	a)	Describe how quantitative variables were handled in the analyses (i.e., scale, units, model)	See 'Statistical methods' section, specifically:<br><br>To improve interpretability and biological relevance, all MR effect estimates have been scaled to represent the effect per one standard deviation (SD) increase in BMI at each of the life stages.
	b)	Describe how genetic variants were handled in the analyses and, if applicable, how their weights were selected	See entire 'Statistical methods' section with details varying throughout the manuscript. Variant selection is specified at relevant analytical stages.
	c)	Describe the MR estimator (e.g. two-stage least squares, Wald ratio) and related statistics. Detail the included covariates and, in case of two-sample MR, whether the same covariate set was used for adjustment in the two samples	Our primary two-sample MR analyses used the inverse variance weighted (IVW) estimator, implemented in the TwoSampleMR R package (*77*). When genetic variants are used as instrumental variables in MR, the assumptions of instrumental variables must be met, i.e., the genetic variants used must (i) be strongly associated with the exposure of interest ("relevance"), (ii) not share common causes with the outcome ("independence"), and (iii) not affect the outcome other than through the exposure ("exclusion-restriction") (*78*). We conducted sensitivity analyses, which relax the assumptions made about horizontal pleiotropy, including MR Egger regression (*79*), and the weighted

				median-based estimator (*80*). We also applied Steiger filtering (*81*) to test the direction of causality between BMI between menarche and first birth and breast cancer risk, by assessing whether the genetic instruments explained more variance in the exposure than in the outcome. In addition we conducted analyses using the MR-RAPS method which is robust to weak instruments and systematic pleiotropy (*82*). For these MR-RAPS analyses, we applied a liberal SNP selection threshold of $P < 1 \times 10^{-5}$ to allow the inclusion of a larger set of instruments. We ran IVW multivariable MR (MVMR), an extension of MR that employs multiple genetic variants associated with multiple measured risk factors, to calculate the direct and indirect effects of BMI between menarche and first birth on breast cancer outcomes.
		d)	Explain how missing data were addressed	A complete-case approach was used, excluding individuals with missing values from the analysis. Due to the skewed distribution of BMI in the data, a natural log transformation was applied prior to running the GWAS analyses within each cohort. Each cohort followed a shared GWAS framework using GCTA v1.93.2beta (*65*). While general quality control (QC) guidelines and analysis scripts were provided via a GitHub repository, individual studies conducted pre-imputation QC independently, applying thresholds appropriate to their cohort data (Supplementary Text and elsewhere (*59, 62-64*)). All analyses were restricted post hoc to common genetic variants with a minor allele frequency (MAF) $\geq$ 0.01.
		e)	If applicable, indicate how multiple testing was addressed	NA
7	**Assessment of assumptions**		Describe any methods or prior knowledge used to assess the assumptions or justify their validity	Our primary two-sample MR analyses used the inverse variance weighted (IVW) estimator, implemented in the TwoSampleMR R package (*77*). When genetic variants are used as instrumental variables in MR, the assumptions of instrumental variables must be met, i.e., the genetic variants used must (i) be strongly associated with the exposure of interest ("relevance"), (ii) not share common causes with the outcome ("independence"), and (iii) not affect the outcome other than through the exposure ("exclusion-restriction") (*78*). We conducted sensitivity analyses, which relax the assumptions made about horizontal pleiotropy, including MR Egger regression (*79*), and the weighted median-based estimator (*80*). We also applied Steiger filtering (*81*) to test the direction of causality between BMI between menarche and first birth and breast cancer risk, by assessing whether the genetic instruments explained more variance in the exposure than in the outcome. In addition we conducted analyses using the MR-RAPS method which is robust to weak instruments and systematic pleiotropy (*82*). To allow the inclusion of a larger set of instruments, we applied a liberal SNP selection threshold of $P < 1 \times 10^{-5}$. We ran IVW multivariable MR (MVMR), an extension of MR that employs multiple genetic variants associated with multiple measured risk factors, to calculate the direct

			and indirect effects of BMI between menarche and first birth on breast cancer outcomes.
8	**Sensitivity analyses and additional analyses**	Describe any sensitivity analyses or additional analyses performed (e.g. comparison of effect estimates from different approaches, independent replication, bias analytic techniques, validation of instruments, simulations)	To minimise pregnancy-related confounding, we restricted the BMI exposure GWAS to nulliparous women, enabling a clearer assessment of BMI's direct influence across life stages. In contrast, the breast cancer outcome GWAS included both nulliparous and parous women. This discrepancy in selection mechanisms introduces potential selection bias in the genetic effect estimates for BMI but not for breast cancer. Specifically, conditioning on parity in the BMI GWAS may induce bias in the estimated SNP effects on BMI ($\hat{\beta}_{Gx}$), whereas breast cancer estimates ($\hat{\beta}_{Gy}$), remain unaffected as parity was not conditioned on in the outcome GWAS (Eq. 1).<br><br>In this two-sample MR setting, the MR estimate is given by:<br><br>(Eq. 1)<br><br>$$\hat{\beta}_{MR} = \frac{\hat{\beta}_{Gy}}{\hat{\beta}_{Gx}}$$<br><br>Where:<br><br>$$E(\hat{\beta}_{Gx}) = \beta_{Gx} + \text{bias}$$<br>$$E(\hat{\beta}_{Gy}) = \beta_{Gy}$$<br><br>Since $\hat{\beta}_{Gy}$ is not subject to the same selection mechanism and there is no reason to assume that the selection-induced bias in β_{Gx} is correlated with $\hat{\beta}_{Gy}$, a major spurious association in the MR analysis is unlikely. However, selection bias in the BMI GWAS could distort the MR estimate, with the direction and magnitude depending on whether and how BMI influences parity within the restricted sample. In an extreme scenario it could induce false positive associations with BMI that arise due to collider bias though this is unlikely at current sample sizes.<br><br>To empirically assess this, we performed univariable MR analyses estimating the effect of parity on BMI in nulliparous women across different life stages (menarche to <40 years, <20 years, 20 to <30 years, and 30 to <40 years). While parity itself cannot causally influence BMI in nulliparous women, genetic variants associated with parity may exhibit pleiotropic effects on BMI through shared metabolic and reproductive pathways. If these parity-associated variants also influence BMI within our restricted sample, it would suggest selection bias related to reproductive behaviour, implying that conditioning on parity in the BMI GWAS may have introduced collider bias. We additionally conducted the

reverse analysis, estimating the effect of early-life BMI-associated variants on parity, to assess the potential for bias in the opposite direction.

In addition, a later onset of menarche has been linked to a reduced risk of breast cancer (*85, 86*). Childhood body size has been shown to accelerate the timing of menarche, whilst an earlier menarche increases the likelihood of increased body size in adulthood (*87, 88*). With these traits sharing a complex and interconnected relationship, we conduct sensitivity analyses estimating the effect of BMI in the lifestages analysed in nulliparous women on overall breast cancer accounting for age at menarche in MVMR analyses. Age at menarche is treated as a confounder, given its influence both BMI and breast cancer risk through hormonal and metabolic pathways. Including it in the MVMR model ensures that observed BMI effects are not simply a reflection of differences in pubertal timing. However, we note that MVMR adjustment for age at menarche is complicated by the substantial genetic overlap between prepubertal BMI and menarche timing (*36*).

9 **Software and pre-registration**

a) Name statistical software and package(s), including version and settings used

We meta-analysed summary statistics from the five cohorts for each lifestage-specific GWAS separately, using a fixed effects model employed in METAL version 2020-05-05 (*67*). We carried out further standard quality control QC procedures (*68*) including tests for bias due to population structure (genomic control inflation factor [λ] and Linkage Disequilibrium Score Regression [LDSC] (*69*)).

We used LD clumping with an r2 threshold of 0.001 and a p-value threshold of 5×10^{-8} to select a set of independent instruments for BMI at each life stage. This was performed using PLINK (*71*) and genotype data from European individuals from phase 3 v5 enrolled in the 1000 genomes project as a reference panel (*72*).

Our primary two-sample MR analyses used the inverse variance weighted (IVW) estimator, implemented in the TwoSampleMR R package (*77*).

We also applied Steiger filtering (*81*) to test the direction of causality between BMI between menarche and first birth and breast cancer risk, by assessing whether the genetic instruments explained more variance in the exposure than in the outcome. In addition we conducted analyses using the MR-RAPS method which is robust to weak instruments and systematic pleiotropy (*82*). To allow the inclusion of a larger set of instruments, we applied a liberal SNP selection threshold of $P < 1 \times 10^{-5}$.

We conducted statistical analyses in R version 4.3.3 (*89*).

		b)	State whether the study protocol and details were pre-registered (as well as when and where)	NA
	**RESULTS**			
10	**Descriptive data**			
		a)	Report the numbers of individuals at each stage of included studies and reasons for exclusion. Consider use of a flow diagram	See Table 1 and Figure 1 and:<br><br>Measurements of body mass index (BMI) between menarche and age <40 years<br>Measurements of body mass index (BMI) between menarche and age <40 years were available for 56,863 nulliparous women across five cohorts: the Avon Longitudinal Study of Parents and Children (ALSPAC), the Trøndelag Health Study (HUNT), the Norwegian Mother, Father and Child Cohort Study (MoBa), Generation R, and Generation Scotland. Corresponding phenotype sample sizes and cohort characteristics for age and BMI are presented in Table 1. Within the primary <40 year group, mean ages at measurement ranged from 23.9 years in ALSPAC to 29.4 years in Generation R, while mean BMI values varied between 22.8 kg/m^2 in ALSPAC and 24.5 kg/m^2 in Generation Scotland. To explore patterns across narrower age intervals within this broader period, we also conducted analyses stratified into three groups: menarche to <20 years, 20 to <30 years, and 30 to <40 years. Phenotypic sample sizes and summary measures of age and BMI for the full <40 group and each stratum are shown in Table 1.<br><br>.<br><br>*Lifestage-stratified genome-wide association studies (GWAS) and meta-analysis*<br><br>Genome-wide association analyses (GWAS) for BMI were conducted in up to 56,628 nulliparous women with measures between menarche and age <40 years, and results were meta-analysed across cohorts. Stratified meta-analyses for the three life stages – menarche to <20 years, 20 to <30 years, and 30 to <40 years – included 11,365, 30,272 and 16,565, respectively (Figure 1). For cohorts with repeat measures within one life stage (e.g., menarche to <40 years), we retained the time point with the largest sample size (e.g., for ALSPAC, only BMI data from participants <20 years were included). Considering results across all GWAS time windows (menarche to <20, 20 to <30, 30 to <40, and the combined menarche to <40), we identified a total of 45 independent variants.
		b)	Report summary statistics for phenotypic exposure(s), outcome(s), and other relevant variables (e.g. means, SDs, proportions)	See Table 1 and Table S10 in Supplementary.
		c)	If the data sources include meta-analyses of previous studies, provide the assessments of heterogeneity across these studies	See Table S2 and:<br><br>Cochran's Q statistic was used to evaluate heterogeneity in SNP-exposure associations across the five cohort studies included in the primary analysis. To

				account for multiple testing across the 31 SNPs, a Bonferroni correction was applied. After adjustment, none of the variants showed evidence of heterogeneity (Qbonf<0.05), suggesting consistent effect estimates across studies. This reinforces their validity in downstream MR analyses, reducing concerns that study-level differences may confound associations with the outcome. Full heterogeneity results, including uncorrected P-values and Q statistics, are provided in Table S2.
		d)	For two-sample MR:<br>i. Provide justification of the similarity of the genetic variant-exposure associations between the exposure and outcome samples<br>ii. Provide information on the number of individuals who overlap between the exposure and outcome studies	See 'Data sources and study design' in the 'Materials and methods' section
11	**Main results**			
		a)	Report the associations between genetic variant and exposure, and between genetic variant and outcome, preferably on an interpretable scale	See Table S1 in Supplementary.
		b)	Report MR estimates of the relationship between exposure and outcome, and the measures of uncertainty from the MR analysis, on an interpretable scale, such as odds ratio or relative risk per SD difference	See Table S4 in Supplementary and the 'Lifecourse Mendelian Randomization (MR) analysis' section in Results, specifically:<br>Univariable MR analyses indicated evidence that a one standard deviation (SD) increase in genetically predicted log-transformed BMI in nulliparous women between menarche and <40 years reduced the risk of overall breast cancer (IVW odds ratio (OR), 95% CI: 0.76, 0.67 to 0.86, P =1.27x10-5) (Figure 4; Table S4). Similar inverse effects were observed across most breast cancer subtypes, except for the HER2-enriched subtype, which showed little evidence of protection. In multivariable MR accounting for later life adult body size, higher genetically predicted BMI in this window continued to provide evidence of a reduced risk of overall breast cancer and most subtypes, with HER2-enriched disease remaining the exception. Adjustment for prepubertal body size led to a marked, though not complete, attenuation of these effects, consistent with part of the protection being shared with childhood adiposity. Some evidence of a protective effect was observed for Luminal B-like (HER2+) tumours (Figure 4; Table S4).<br>In the narrower life-stage periods obtained by partitioning the broader fertile-window measure, univariable MR analyses indicated evidence that higher genetically predicted BMI in nulliparous women between menarche to <20, 20 to <30, and 30 to <40 years reduced the risk of overall breast cancer and most subtypes, although the strength and precision of effects varied by subtype and life-stage period. After accounting for later life adult body size, protective effects were largely retained, with most estimates still supporting a reduction in risk. In contrast, adjusting for prepubertal body size removed much of the

				apparent protection in these narrower periods. For triple-negative disease, effect estimates suggested modest residual protection in the 20 to <30 window after accounting for childhood body size.
		c)	If relevant, consider translating estimates of relative risk into absolute risk for a meaningful time period	NA
		d)	Consider plots to visualize results (e.g. forest plot, scatterplot of associations between genetic variants and outcome versus between genetic variants and exposure)	See entire 'Results' section as well as Supplementary Figures S1-S5.
12	**Assessment of assumptions**			
		a)	Report the assessment of the validity of the assumptions	See Table S4 as well as the Lifecourse Mendelian randomization (MR) analysis section in Results, specifically:<br><br>Weighted median estimates were consistent in direction and magnitude. MR-Egger analyses provided little evidence of horizontal pleiotropy for the combined menarche to <40 exposure; for the narrower life-stage windows, estimates were imprecise due to limited statistical power (Table S4). To further assess the validity of the inferred causal direction, we applied Steiger filtering, which tests whether the genetic instruments explain more variance in the exposure than in the outcome. Across all life stages, the proportion of variance explained (R^2) was consistently higher for BMI than for breast cancer, supporting the hypothesised direction of effect from BMI to breast cancer (Table S5).<br><br>Using Mendelian Randomization Robust Adjusted Profile Score (MR-RAPS) with an instrument selection threshold of $P < 1 \times 10^{-5}$, genetically predicted BMI between menarche and <40 years, as well as between 20 and <30 years, showed strong evidence of a protective effect on overall breast cancer risk. Weaker evidence was observed for BMI between 30 and <40 years, while very little evidence of an effect was found for BMI between menarche and <20 years (Table S6).<br><br>Multivariable MR results should be interpreted with caution since conditional F-statistic was <10 for each of the BMI exposures included in these models, indicating potential for weak instrument bias (Table S4).
		b)	Report any additional statistics (e.g., assessments of heterogeneity across genetic variants, such as I^2, Q statistic or E-value)	See entire 'Lifestage-stratified genome-wide association studies (GWAS) and meta-analysis' section in Results and Table S2
13	**Sensitivity analyses and additional analyses**			

	a) Report any sensitivity analyses to assess the robustness of the main results to violations of the assumptions	See Table S4 as well as the Lifecourse Mendelian randomization (MR) analysis section in Results, specifically:<br><br>Weighted median estimates were consistent in direction and magnitude. MR-Egger analyses provided little evidence of horizontal pleiotropy for the combined menarche to <40 exposure; for the narrower life-stage windows, estimates were imprecise due to limited statistical power (Table S4). To further assess the validity of the inferred causal direction, we applied Steiger filtering, which tests whether the genetic instruments explain more variance in the exposure than in the outcome. Across all life stages, the proportion of variance explained (R^2) was consistently higher for BMI than for breast cancer, supporting the hypothesised direction of effect from BMI to breast cancer (Table S5).<br><br>Using Mendelian Randomization Robust Adjusted Profile Score (MR-RAPS) with an instrument selection threshold of $P < 1 \times 10^{-5}$, genetically predicted BMI between menarche and <40 years, as well as between 20 and <30 years, showed strong evidence of a protective effect on overall breast cancer risk. Weaker evidence was observed for BMI between 30 and <40 years, while very little evidence of an effect was found for BMI between menarche and <20 years (Table S6).
	b) Report results from other sensitivity analyses or additional analyses	To minimise pregnancy-related confounding, we restricted the BMI GWAS to nulliparous women, enabling a clearer assessment of BMI's direct influence across life stages. However, this restriction may introduce selection (collider) bias if genetically proxied parity is associated with BMI within the nulliparous sample, as parity was not conditioned on in the breast cancer outcome GWAS. Our analyses indicated very little evidence that genetically proxied parity associated with BMI in nulliparous women between menarche and <40 years, menarche and <20 years, and 20 and <30 years. There was some evidence that genetically proxied parity associated with lower BMI in nulliparous women between 30 and <40 years (Table S7). There was very little evidence of effect in the reverse direction (BMI-associated variants on parity). These results suggest collider bias due to selecting nulliparous women is likely to be of little concern within this study.<br><br>After adjusting for age at menarche, the estimated effect of BMI in nulliparous women between menarche and <40 years on overall breast cancer remains largely consistent with the UVMR analyses (Table S8). Age at menarche itself showed little direct effect on overall breast cancer or most subtypes once BMI was accounted for.
	c) Report any assessment of direction of causal relationship (e.g., bidirectional MR)	To further assess the validity of the inferred causal direction, we applied Steiger filtering, which tests whether the genetic instruments explain more variance in the exposure than in the outcome. Across all life stages, the

				proportion of variance explained (R^2) was consistently higher for BMI than for breast cancer, supporting the hypothesised direction of effect from BMI to breast cancer (Table S5).
		d)	When relevant, report and compare with estimates from non-MR analyses	See Discussion section.
		e)	Consider additional plots to visualize results (e.g., leave-one-out analyses)	See entire 'Results' section.
	**DISCUSSION**			
14	**Key results**		Summarize key results with reference to study objectives	In this study, we undertook GWAS and MR analyses within a lifecourse framework to (i) assess the consistency of the genetic effects of BMI across different life stages, and (ii) investigate the effects of genetically proxied BMI in nulliparous women between menarche and <40 years on the risk of overall breast cancer and seven subtypes. This work builds on previous research to further the investigation into the influence of BMI during critical periods on breast cancer risk (*13, 14, 35*). Motivated by recent findings suggesting that a larger prepubertal body size, used as a proxy for BMI, may offer protection against breast cancer risk, while later life body size shows very little effect, we aimed to better understand the interval between puberty and later life. Specifically, we focused on the period between menarche and first full-term pregnancy, a crucial window of vulnerability for later life breast cancer development.<br><br>We observed variation in genetic effects on BMI across different life stages for nine of the 45 discovery variants identified in this study. Some of this variation may reflect statistical fluctuation introduced by selecting lead SNPs based on age-stratified significance thresholds. However, several variants showed clear changes in the direction of effect across the life stage groups. While some directional shifts may reflect genuine developmental effects, we cannot conclusively distinguish these from statistical artifacts that may arise from mechanisms such as the winner's curse or age-related selection bias, which could exaggerate apparent fluctuations in effect size.In addition, genome-wide cross-lifestage genetic correlations demonstrated variation, e.g., when comparing prepubertal body size and later life body size with BMI from menarche to <40 years.<br><br>In the univariable MR analyses, higher BMI in nulliparous women from menarche to <40 years was found to consistently reduce risk of overall breast cancer and most subtypes. Similar protective effects were observed when BMI was measured within narrower periods of this window (menarche to <20, 20 to <30, and 30 to <40 years). These protective effects largely remained after accounting for later life adult body size. In contrast, adjusting for prepubertal body size in multivariable MR led to greater attenuation, indicating that part of

			the protective effect of BMI in early reproductive life may be explained by body size before puberty. The attenuation was more pronounced for the narrower life-stage periods than for the full fertile-window measure, reflecting both biological and methodological influences. Biologically, part of the apparent effect across all reproductive-age windows overlaps with childhood adiposity, so adjustment for prepubertal body size reduces the estimates. Methodologically, the narrower age-specific GWASs have substantially smaller sample sizes than the childhood GWAS. In multivariable MR, this imbalance can magnify the degree of attenuation by limiting power to detect effects independent of childhood size. By contrast, the broader reproductive-age measure is based on a larger GWAS sample and therefore shows less attenuation, despite adjustment for the same childhood body size data. Importantly, no such attenuation was observed when adjusting for later-life body size, even though similar sample size imbalances exist. This indicates that differential power alone may not fully explain the observed differences. Future methodological work is needed to examine how sample size disparities across life-stage GWASs influence estimates in multivariable MR and to develop strategies for accounting for this in lifecourse applications.
15	**Limitations**	Discuss limitations of the study, taking into account the validity of the IV assumptions, other sources of potential bias, and imprecision. Discuss both direction and magnitude of any potential bias and any efforts to address them	This study has several limitations that should be considered when interpreting the findings. First, low conditional F-statistics were observed in the MVMR analysis, indicating potential for weak instrument bias. As a result, our MVMR findings should be interpreted with some caution. However, the pattern of attenuation observed when adjusting for pre-pubertal body size was not replicated when adjusting for adult body size, despite both exposures being derived from GWAS with similar sample sizes and instrument strength. This asymmetry supports the interpretation that the differences in effect estimates are unlikely to be driven by weak instrument bias alone and likely reflect biologically distinct influences of body size at different developmental stages. In addition, while we combined data from five large European longitudinal cohorts, the sample size remains smaller (N=56,628) than that available for comparable measures of prepubertal and later-life body size derived using in UK Biobank data (N=246,511). This reduced sample size may limit statistical power for detecting smaller effect sizes. Second, we used a prepubertal GWAS based on reported body size rather than measured BMI, as it offered a much larger sample size than any other GWAS of BMI at this life stage. However, this choice may distort MVMR analysis due to measurement error or differences in variance of effect sizes. Future work to precisely estimate childhood body size genetic effects are warranted. Third, there is the possibility of participant overlap between the HUNT and MoBa cohorts. The HUNT Study began in 1984-86, recruiting adults aged ≥20 in Nord-Trøndelag County across four cycles for over

				30 years (*50*). Its Young-HUNT sub-studies recruited adolescents aged 13-19 in four waves between 1995 and 2019. MoBa, in contrast, recruited pregnant women nationally between 1999 and 2008 via routine prenatal care (*51*). While a small number of individuals may have participated in both, up to 10% of MoBa participants were recruited from the catchment area for the HUNT surveys used in this study, suggesting any analytic overlap is likely minimal. Some participants in HUNT may have also participated in MoBa, which could introduce a degree of sample duplication. Fourth, the inclusion criteria for this study resulted in substantially smaller analytic samples compared to the total number of participants enrolled in each cohort. Although genotyping was not based on specific phenotypes, we acknowledge that, as in all large-scale genetic studies, selection bias due to differential data availability may still be present. Lastly, as our analysis was restricted to individuals of European ancestry, the generalisability of our findings to other populations is limited. Further research is needed to confirm these findings in more diverse populations.
16	**Interpretation**			
		a)	Meaning: Give a cautious overall interpretation of results in the context of their limitations and in comparison with other studies	See entire Discussion section, specifically:<br><br>This study offers important insights into the genetic influences on BMI across different life stages and its causal relationship with breast cancer risk, focusing on the period between menarche and under 40 years in nulliparous women as a key window of susceptibility. While higher BMI in this interval appeared strongly protective against breast cancer in univariable analyses, the effect substantially, though not entirely, attenuated after accounting for childhood body size, used as a proxy for BMI. This pattern suggests that the protection may arise from the combined influence of greater adiposity in both childhood and early adulthood. These results have important implications for breast cancer prevention, underscoring earlier life stages as critical periods for potential interventions.
		b)	Mechanism: Discuss underlying biological mechanisms that could drive a potential causal relationship between the investigated exposure and the outcome, and whether the gene-environment equivalence assumption is reasonable. Use causal language carefully, clarifying that IV estimates may provide causal effects only under certain assumptions	Evidence suggests that two distinct early-life pathways influence breast cancer risk: one linked to greater body size and the other to earlier age at menarche (*13*). While a larger body size in early life appears protective, it may also accelerate age at menarche, which is itself a risk factor for breast cancer, suggesting that these pathways are not only independent but may interact in complex ways. To isolate the direct effect of BMI independent of pubertal timing, we applied MVMR controlling for age at menarche. However, the genetic architecture of age at menarche and early-life adiposity overlaps substantially (*36*). A recent genome-wide structural equation model integrating MoBa childhood BMI, recalled adiposity at age 10 in UK Biobank, and age at menarche estimated that nearly half of the genetic variants associated with menarche act through early-life adiposity (*36, 37*). This overlap has also been supported by longitudinal twin studies (*38*). While this indicates strong shared

genetic pathways, a proportion of menarche-associated variants likely do act independently of adiposity. Nonetheless, BMI-related pathways to earlier menarche may contribute more strongly to identified SNP signals than non-BMI influences, complicating interpretation of MVMR estimates. Thus, although MVMR provides a valuable sensitivity analysis, it may not fully disentangle the independent contributions of adiposity and pubertal timing, particularly where shared pathways act differently across breast cancer subtypes. Furthermore, increased prepubertal body size has been shown to decrease breast density, while age at menarche has been linked to higher breast density, which is another important risk factor for breast cancer (*35, 39*). This distinction between pathways highlights the need to examine potential mediating factors, which may help clarify the mechanisms at play. A deeper investigation into these separate but intersecting pathways could reveal novel insights into breast cancer risk.

The relationship between body size and breast cancer risk is further complicated by menopausal status. In conventional epidemiological settings, higher BMI has been linked to an increased risk of breast cancer in postmenopausal women and a decreased risk of breast cancer in premenopausal women (*40-43*). A plausible explanation includes the differing levels of oestrogen exposure between women experiencing overweight and normal-weight (*44*). Premenopausal women experiencing overweight tend to have longer anovulatory cycles, which reduces their exposure to ovarian hormones, potentially lowering their breast cancer risk. After menopause, fat tissue serves as another source of oestrogen production, which increases breast cancer risk among overweight women (*45-48*). Whilst our study did not specifically examine the relationship between overweight status and breast cancer pre- and post- menopause, effect estimates, effect estimates were broadly consistent across subtypes, with only modest differences in magnitude. The most evident departure from this pattern was seen in the HER2-enriched subtype, which showed little evidence of protection in either univariable or multivariable MR, potentially reflecting its distinct biology and the clinical observation that overweight and obesity can worsen prognosis in early-stage HER2-positive breast cancer (*49*). Lacking hormonal regulation, HER2-enriched tumours that depend on aberrant HER2 pathway activation may be less affected by adiposity-related changes in oestrogen, SHBG, or IGF-1. In contrast, Luminal B-like (HER2+) tumours appeared to retain evidence of a residual protective effect across the full reproductive-life window (menarche to <40 years), even after adjustment for prepubertal body size. This difference may reflect the continued influence of hormone-dependent pathways in Luminal B-like (HER2+), which could mediate BMI-related protection through endocrine or metabolic mechanisms despite HER2 expression. Previous MR studies of childhood body size support such subtype-specific mechanisms; for example, mammographic density, particularly dense area, appears to mediate much of the protective effect for ER-positive

			but not ER-negative breast cancer (*35*), and hormonal mediators such as IGF-1 and SHBG appear relevant mainly for ER-positive disease (*13*). These findings underscore the importance of considering breast cancer subtypes when evaluating the long-term effects of adiposity across the lifecourse.
		c) Clinical relevance: Discuss whether the results have clinical or public policy relevance, and to what extent they inform effect sizes of possible interventions	This study is an important and novel analysis with multiple strengths. It focuses on a previously unexamined life stage – the period between menarche and under 40 years in nulliparous women – to investigate the impact of increased BMI on breast cancer risk using causal inference methods. By examining this life stage, we provide new evidence on how adiposity before and during early reproductive life may influence breast cancer development. This focus also minimises potential confounding from the physiological changes associated with a first pregnancy, allowing clearer interpretation of life-stage-specific effects. To strengthen the analysis, we integrated data from five large European longitudinal cohort studies. This approach allowed us to gain key insights into the consistency of BMI-related genetic effects across different stages of life. In addition, these data are not only useful for this particular study but also offer a valuable resource for future research into the effects of BMI in women at this life stage on other health outcomes.
17	**Generalizability**	Discuss the generalizability of the study results (a) to other populations, (b) across other exposure periods/timings, and (c) across other levels of exposure	Lastly, as our analysis was restricted to individuals of European ancestry, the generalisability of our findings to other populations is limited. Further research is needed to confirm these findings in more diverse populations.
	**OTHER INFORMATION**		
18	**Funding**	Describe sources of funding and the role of funders in the present study and, if applicable, sources of funding for the databases and original study or studies on which the present study is based	The UK Medical Research Council and Wellcome (Grant ref: 217065/Z/19/Z) and the University of Bristol provide core support for ALSPAC. GMP, AH, GL, ES, RR, GH, and GDS were supported by the Integrative Epidemiology Unit which receives funding from the UK Medical Research Council and the University of Bristol (MC_UU_00032/1). GMP was additionally supported by the University of Bristol Cancer research fund for this work. RR is supported by Cancer Research UK [grant number C18281/A29019]. GDS conducts research at the NIHR Biomedical Research Centre at the University Hospitals Bristol NHS Foundation Trust and the University of Bristol. The views expressed in this publication are those of the author(s) and not necessarily those of the NHS, the National Institute for Health Research or the Department of Health. The genotyping in HUNT was supported by the National Institutes of Health (NIH); University of Michigan; The Research Council of Norway (RCN); The Liaison Committee for Education, Research and Innovation in Central Norway; and the Joint Research Committee between St. Olavs hospital and the Faculty of Medicine and Health Sciences, NTNU. LB, BOA, and BMB work in a research unit financially supported by the Liaison Committee for education, research and innovation in Central Norway and the Joint Research Committee between St. Olavs Hospital and the

			Faculty of Medicine and Health Sciences, NTNU. This study used data from Medical Birth Registry of Norway (MBRN). Generation Scotland received core support from the Chief Scientist Office of the Scottish Government Health Directorates [CZD/16/6] and the Scottish Funding Council [HR03006] and is currently supported by the Wellcome Trust [216767/Z/19/Z]. Genotyping of the GS:SFHS samples was carried out by the Genetics Core Laboratory at the Edinburgh Clinical Research Facility, University of Edinburgh, Scotland and was funded by the Medical Research Council UK and the Wellcome Trust (Wellcome Trust Strategic Award "STratifying Resilience and Depression Longitudinally" (STRADL) Reference 104036/Z/14/Z. CH was supported by an MRC University Unit core grant MC_UU_00007/10 (QTL in Health and Disease program). BLL acknowledges support from the University of Bristol (Vice-Chancellor's Research Fellowship), Academy of Medical Sciences/Wellcome Trust/the Government Department of Business, Energy and Industrial Strategy/British Heart Foundation/Diabetes UK Springboard Award (SBF003/1170), Elizabeth Blackwell Institute for Health Research (University of Bristol), and Wellcome Trust Institutional Strategic Support Fund (204813/Z/16/Z) and Wellcome Trust Career Development Award (227849/Z/23/Z). AH was supported by the Research Council of Norway (#336085) and the South-Eastern Norway Regional Health Authority (#2020022; #2922083; #2019097; #2018059; #2021045). The general design of the Generation R Study is made possible by financial support from Erasmus MC, University Medical Center Rotterdam, Erasmus University Rotterdam, the Netherlands Organization for Health Research and Development (ZonMw), the Netherlands Organization for Scientific Research (NWO), the Ministry of Health, Welfare and Sport, and the Ministry of Youth and Families. The parental genotyping in Generation R was supported by the ERC under the European Union's Horizon 2020 research and innovation programme (iRISK; grant agreement No 863981). This project received funding from the European Union's Horizon Europe Research and Innovation Programme under grant agreement nº 101137146 (STAGE project). UK participants in Horizon Europe Project STAGE are supported by UKRI grant numbers 10112787 (Beta Technology), 10099041 (University of Bristol) and 10109957 (Imperial College London).
19	**Data and data sharing**	Provide the data used to perform all analyses or report where and how the data can be accessed and reference these sources in the article. Provide the statistical code needed to reproduce the results in the article, or report whether the code is publicly accessible and if so, where	Data access for the Avon Longitudinal Study of Parents and Children (ALSPAC) operates via a managed open access system. Approved proposals are reviewed by the ALSPAC Executive Committee. Full details are provided in the ALSPAC Data Management Plan (www.bristol.ac.uk/alspac/researchers/data-access/documents/alspac-data-management-plan.pdf). Data from the Norwegian Mother, Father and Child (MoBa) Cohort Study is managed by the Norwegian Institute of Public Health. Access is provided upon approval from the Regional Committees for Medical and Health Research Ethics (REC), compliance

			with GDPR, and data owner approval. Participant consent does not allow individual-level data storage in repositories or journals. Researchers seeking access for replication must apply via www.helsedata.no. To request access to data from the Trøndelag Health Study (HUNT), researchers affiliated with Norwegian research institutes can apply for the use of HUNT data and biological samples, subject to approval by the Regional Committee for Medical and Health Research Ethics. Researchers from other countries may also apply if collaborating with a Norwegian Principal Investigator. All applications are reviewed by the HUNT Data Access Committee, and successful applicants are required to enter into data access and/or material transfer agreements. Detailed information on the application process, ethical requirements, and available datasets can be found on the HUNT website (www.ntnu.edu/hunt/data). Data from Generation Scotland are available on application to an independent access committee. More information can be found on the Generation Scotland website (www.generationscotland.org). Data from the Generation R Study are available upon reasonable request to the director of the Generation R Study (generationr@erasmusmc.nl), subject to local, national and European rules and regulations.<br><br>All genetic instruments derived in this study are in the supplementary tables. Genome-wide association study summary statistics will be made available on a public repository upon publication.<br><br>The standard operating procedure (SOP) and analysis scripts used to conduct the genome-wide association study (GWAS) of BMI between menarche and first birth are available in a GitHub repository:<br><br>https://github.com/gracemarionpower/Collaboration_MA_BMI_M2FB.<br>A citable snapshot of the repository is archived on Zenodo: https://doi.org/10.5281/zenodo.17178557<br><br>None of the material has been published or is under consideration for publication elsewhere.
20	**Conflicts of Interest**	All authors should declare all potential conflicts of interest	Daniel McCartney is an employee of Optima Partners Ltd. All authors declare no other competing interests.

This checklist is copyrighted by the Equator Network under the Creative Commons Attribution 3.0 Unported (CC BY 3.0) license.

Table S10. Summary of Breast Cancer Genome-Wide Association Study Datasets. Summary of breast cancer genome-wide association study (GWAS) datasets from the BCAC 2017 and BCAC 2020 releases.

Phenotype	Subtype	Receptor/grade status	Lead author name	Pubmed ID	Release	Sample size (female)	Cases	Controls	% cases
Breast cancer	ER+	ER+	Michailidou K	29059683	2017	175,475	69,501	105,974	39.60%
Breast cancer	ER-	ER-				127,442	21,468	105,974	16.90%
Breast cancer	Luminal A-like	ER+ and/or PR+, HER2−, grades 1 and 2	Zhang H	32424353	2020	155,244	63,767	91,477	41.10%
Breast cancer	Luminal B-like/HER2+	ER+ and/or PR+, HER2+				107,419	15,942	91,477	14.80%
Breast cancer	Luminal B/HER2-negative-like	ER+ and/or PR+, HER2−, grade 3				107,419	15,942	91,477	14.80%
Breast cancer	HER2-enriched-like	ER− and PR−, HER2+				102,105	10,628	91,477	10.40%
Breast cancer	Triple-negative	ER− and PR−, HER2−				100,079	8,602	91,477	8.60%
Prepubertal body size	N/A	N/A	Richardson TG	32376654	2020	246,511	N/A	N/A	N/A
Later life adult body size	N/A	N/A				246,511	N/A	N/A	N/A

REFERENCES

1. M. Arnold, E. Morgan, H. Rumgay, A. Mafra, D. Singh, M. Laversanne, J. Vignat, J. R. Gralow, F. Cardoso, S. Siesling, I. Soerjomataram, Current and future burden of breast cancer: Global statistics for 2020 and 2040. *Breast* **66**, 15–23 (2022).

2. C. S. W. Group., U.S. Cancer Statistics Data Visualizations Tool, based on 2021 submission data (1999–2020): U.S. Department of Health and Human Services, Centers for Disease Control and Prevention and National Cancer Institute. (Updated June 2024).

3. Y. Liu, D. K. Tobias, K. M. Sturgeon, B. Rosner, V. Malik, E. Cespedes, A. D. Joshi, A. H. Eliassen, G. A. Colditz, Physical activity from menarche to first pregnancy and risk of breast cancer. *Int. J. Cancer* **139**, 1223–1230 (2016).

4. G. A. Colditz, K. Bohlke, C. S. Berkey, Breast cancer risk accumulation starts early: Prevention must also. *Breast Cancer Res. Treat.* **145**, 567–579 (2014).

5. M. Ciwinska, H. A. Messal, H. R. Hristova, C. Lutz, L. Bornes, T. Chalkiadakis, R. Harkes, N. S. M. Langedijk, S. J. Hutten, R. X. Menezes, J. Jonkers, S. Prekovic, Grand Challenge PRECISION consortium, B. D. Simons, C. L. G. J. Scheele, J. van Rheenen, Mechanisms that clear mutations drive field cancerization in mammary tissue. *Nature* **633**, 198–206 (2024).

6. Y. Liu, G. A. Colditz, B. Rosner, C. S. Berkey, L. C. Collins, S. J. Schnitt, J. L. Connolly, W. Y. Chen, W. C. Willett, R. M. Tamimi, Alcohol intake between menarche and first pregnancy: A prospective study of breast cancer risk. *J. Natl. Cancer Inst.* **105**, 1571–1578 (2013).

7. IARC Working Group on the Evaluation of Carcinogenic Risks to Humans, Alcohol consumption and ethyl carbamate. *IARC Monogr. Eval. Carcinog. Risks Hum.* **96**, 3–1383 (2010).

8. G. Davey Smith, S. Ebrahim, 'Mendelian randomization': Can genetic epidemiology contribute to understanding environmental determinants of disease? *Int. J. Epidemiol.* **32**, 1–22 (2003).

9. E. Sanderson, M. M. Glymour, M. V. Holmes, H. Kang, J. Morrison, M. R. Munafò, T. Palmer, C. M. Schooling, C. Wallace, Q. Zhao, G. Davey Smith, Mendelian randomization. *Nat. Rev. Methods Primers* **2**, 6 (2022).

10. G. M. Power, E. Sanderson, P. Pagoni, A. Fraser, T. Morris, C. Prince, T. M. Frayling, J. Heron, T. G. Richardson, R. Richmond, J. Tyrrell, N. Warrington, G. Davey Smith, L. D. Howe, K. M. Tilling, Methodological approaches, challenges, and opportunities in the application of Mendelian randomisation to lifecourse epidemiology: A systematic literature review. *Eur. J. Epidemiol.* **39**, 501–520 (2024).

11. E. Sanderson, G. Davey Smith, F. Windmeijer, J. Bowden, An examination of multivariable Mendelian randomization in the single-sample and two-sample summary data settings. *Int. J. Epidemiol.* **48**, 713–727 (2019).

12. E. Sanderson, T. G. Richardson, T. T. Morris, K. Tilling, G. Davey Smith, Estimation of causal effects of a time-varying exposure at multiple time points through multivariable mendelian randomization. *PLOS Genet.* **18**, e1010290 (2022).

13. M. Vabistsevits, G. Davey Smith, E. Sanderson, T. G. Richardson, B. Lloyd-Lewis, R. C. Richmond, Deciphering how early life adiposity influences breast cancer risk using Mendelian randomization. *Commun. Biol.* **5**, 337 (2022).

14. T. G. Richardson, E. Sanderson, B. Elsworth, K. Tilling, G. Davey Smith, Use of genetic variation to separate the effects of early and later life adiposity on disease risk: Mendelian randomisation study. *BMJ* **369**, m1203 (2020).

15. Y. Hao, J. Xiao, Y. Liang, X. Wu, H. Zhang, C. Xiao, L. Zhang, S. Burgess, N. Wang, X. Zhao, P. Kraft, J. Li, X. Jiang, Reassessing the causal role of obesity in breast cancer susceptibility: A comprehensive multivariable Mendelian randomization investigating the distribution and timing of exposure. *Int. J. Epidemiol.* **52**, 58–70 (2023).

16. G. M. Power, T. Palmer, N. Warrington, J. Heron, T. G. Richardson, V. Didelez, K. Tilling, G. Davey Smith, E. Sanderson, A structural mean modelling Mendelian randomization approach

to investigate the lifecourse effect of adiposity: Applied and methodological considerations. *Am. J. Epidemiol.* , kwaf029 (2025).

17. B. W. Jensen, J. Aarestrup, K. Blond, M. E. Jørgensen, A. G. Renehan, D. Vistisen, J. L. Baker, Childhood body mass index trajectories, adult-onset type 2 diabetes, and obesity-related cancers. *J. Natl. Cancer Inst.* **115**, 43–51 (2023).

18. G. Fagherazzi, G. Guillas, M.-C. Boutron-Ruault, F. Clavel-Chapelon, S. Mesrine, Body shape throughout life and the risk for breast cancer at adulthood in the French E3N cohort. *Eur. J. Cancer Prev.* **22**, 29–37 (2013).

19. M. Song, W. C. Willett, F. B. Hu, D. Spiegelman, A. Must, K. Wu, A. T. Chan, E. L. Giovannucci, Trajectory of body shape across the lifespan and cancer risk. *Int. J. Cancer* **138**, 2383–2395 (2016).

20. T. O. Yang, B. J. Cairns, K. Pirie, J. Green, V. Beral, S. Floud, G. K. Reeves, Body size in early life and the risk of postmenopausal breast cancer. *BMC Cancer* **22**, 232 (2022).

21. A. Llewellyn, M. Simmonds, C. G. Owen, N. Woolacott, Childhood obesity as a predictor of morbidity in adulthood: A systematic review and meta-analysis. *Obes. Rev.* **17**, 56–67 (2016).

22. D. Byun, S. Hong, S. Ryu, Y. Nam, H. Jang, Y. Cho, N. Keum, H. Oh, Early-life body mass index and risks of breast, endometrial, and ovarian cancers: A dose-response meta-analysis of prospective studies. *Br. J. Cancer* **126**, 664–672 (2022).

23. Z. J. Andersen, J. L. Baker, K. Bihrmann, I. Vejborg, T. I. Sørensen, E. Lynge, Birth weight, childhood body mass index, and height in relation to mammographic density and breast cancer: A register-based cohort study. *Breast Cancer Res.* **16**, R4 (2014).

24. T. G. Richardson, H. Urquijo, M. V. Holmes, G. Davey Smith, Leveraging family history data to disentangle time-varying effects on disease risk using lifecourse mendelian randomization. *Eur. J. Epidemiol.* **38**, 765–769 (2023).

25. C. M. Schooling, K. Fei, J. V. Zhao, Selection bias as an explanation for the observed protective association of childhood adiposity with breast cancer. *J. Clin. Epidemiol.* **164**, 104–111 (2023).

26. A. Gkatzionis, S. R. Seaman, R. A. Hughes, K. Tilling, Relationship between collider bias and interactions on the log-additive scale. *Stat. Methods Med. Res.* **34**, 1063–1078 (2025).

27. G. M. Power, E. Sanderson, G. Davey Smith, G. Hemani, Selection bias is unlikely to fully explain the protective effect of childhood adiposity on breast cancer risk. medRxiv 25335479 [Preprint] (2025). https://doi.org/10.1101/2025.09.10.25335479.

28. K. Michailidou, S. Lindström, J. Dennis, J. Beesley, S. Hui, S. Kar, A. Lemaçon, P. Soucy, D. Glubb, A. Rostamianfar, M. K. Bolla, Q. Wang, J. Tyrer, E. Dicks, A. Lee, Z. Wang, J. Allen, R. Keeman, U. Eilber, J. D. French, X. Q. Chen, L. Fachal, K. M. Cue, A. E. McCart Reed, M. Ghoussaini, J. S. Carroll, X. Jiang, H. Finucane, M. Adams, M. A. Adank, H. Ahsan, K. Aittomäki, H. Anton-Culver, N. N. Antonenkova, V. Arndt, K. J. Aronson, B. Arun, P. L. Auer, F. Bacot, M. Barrdahl, C. Baynes, M. W. Beckmann, S. Behrens, J. Benitez, M. Bermisheva, L. Bernstein, C. Blomqvist, N. V. Bogdanova, S. E. Bojesen, B. Bonanni, A.-L. Børresen-Dale, J. S. Brand, H. Brauch, P. Brennan, H. Brenner, L. Brinton, P. Broberg, I. W. Brock, A. Broeks, A. Brooks-Wilson, S. Y. Brucker, T. Brüning, B. Burwinkel, K. Butterbach, Q. Cai, H. Cai, T. Caldés, F. Canzian, A. Carracedo, B. D. Carter, J. E. Castelao, T. L. Chan, T.-Y. D. Cheng, K. S. Chia, J.-Y. Choi, H. Christiansen, C. L. Clarke, NBCS Collaborators, M. Collée, D. M. Conroy, E. Cordina-Duverger, S. Cornelissen, D. G. Cox, A. Cox, S. S. Cross, J. M. Cunningham, K. Czene, M. B. Daly, P. Devilee, K. F. Doheny, T. Dörk, I. Dos-Santos-Silva, M. Dumont, L. Durcan, M. Dwek, D. M. Eccles, A. B. Ekici, A. H. Eliassen, C. Ellberg, M. Elvira, C. Engel, M. Eriksson, P. A. Fasching, J. Figueroa, D. Flesch-Janys, O. Fletcher, H. Flyger, L. Fritschi, V. Gaborieau, M. Gabrielson, M. Gago-Dominguez, Y.-T. Gao, S. M. Gapstur, J. A. García-Sáenz, M. M. Gaudet, V. Georgoulias, G. G. Giles, G. Glendon, M. S. Goldberg, D. E. Goldgar, A. González-Neira, G. I. Grenaker Alnæs, M. Grip, J. Gronwald, A. Grundy, P. Guénel, L. Haeberle, E. Hahnen, C. A. Haiman, N. Håkansson, U. Hamann, N. Hamel, S. Hankinson, P. Harrington, S. N. Hart, J. M. Hartikainen, M. Hartman, A. Hein, J. Heyworth, B. Hicks, P. Hillemanns, D. N. Ho, A. Hollestelle, M. J. Hooning, R. N. Hoover, J. L. Hopper, M.-F. Hou, C.-N. Hsiung, G. Huang, K. Humphreys, J. Ishiguro, H.

Ito, M. Iwasaki, H. Iwata, A. Jakubowska, W. Janni, E. M. John, N. Johnson, K. Jones, M. Jones, A. Jukkola-Vuorinen, R. Kaaks, M. Kabisch, K. Kaczmarek, D. Kang, Y. Kasuga, M. J. Kerin, S. Khan, E. Khusnutdinova, J. I. Kiiski, S.-W. Kim, J. A. Knight, V.-M. Kosma, V. N. Kristensen, U. Krüger, A. Kwong, D. Lambrechts, L. L. Marchand, E. Lee, M. H. Lee, J. W. Lee, C. N. Lee, F. Lejbkowicz, J. Li, J. Lilyquist, A. Lindblom, J. Lissowska, W.-Y. Lo, S. Loibl, J. Long, A. Lophatananon, J. Lubinski, C. Luccarini, M. P. Lux, E. S. K. Ma, R. J. MacInnis, T. Maishman, E. Makalic, K. E. Malone, I. M. Kostovska, A. Mannermaa, S. Manoukian, J. A. E. Manson, S. Margolin, S. Mariapun, M. E. Martinez, K. Matsuo, D. Mavroudis, J. M. Kay, C. M. Lean, H. Meijers-Heijboer, A. Meindl, P. Menéndez, U. Menon, J. Meyer, H. Miao, N. Miller, N. A. M. Taib, K. Muir, A. M. Mulligan, C. Mulot, S. L. Neuhausen, H. Nevanlinna, P. Neven, S. F. Nielsen, D.-Y. Noh, B. G. Nordestgaard, A. Norman, O. I. Olopade, J. E. Olson, H. Olsson, C. Olswold, N. Orr, V. S. Pankratz, S. K. Park, T.-W. Park-Simon, R. Lloyd, J. I. A. Perez, P. Peterlongo, J. Peto, K.-A. Phillips, M. Pinchev, D. Plaseska-Karanfilska, R. Prentice, N. Presneau, D. Prokofyeva, E. Pugh, K. Pylkäs, B. Rack, P. Radice, N. Rahman, G. Rennert, H. S. Rennert, V. Rhenius, A. Romero, J. Romm, K. J. Ruddy, T. Rüdiger, A. Rudolph, M. Ruebner, E. J. T. Rutgers, E. Saloustros, D. P. Sandler, S. Sangrajrang, E. J. Sawyer, D. F. Schmidt, R. K. Schmutzler, A. Schneeweiss, M. J. Schoemaker, F. Schumacher, P. Schürmann, R. J. Scott, C. Scott, S. Seal, C. Seynaeve, M. Shah, P. Sharma, C.-Y. Shen, G. Sheng, M. E. Sherman, M. J. Shrubsole, X.-O. Shu, A. Smeets, C. Sohn, M. C. Southey, J. J. Spinelli, C. Stegmaier, S. Stewart-Brown, J. Stone, D. O. Stram, H. Surowy, A. Swerdlow, R. Tamimi, J. A. Taylor, M. Tengström, S. H. Teo, M. B. Terry, D. C. Tessier, S. Thanasitthichai, K. Thöne, R. A. E. M. Tollenaar, I. Tomlinson, L. Tong, D. Torres, T. Truong, C.-C. Tseng, S. Tsugane, H.-U. Ulmer, G. Ursin, M. Untch, C. Vachon, C. J. van Asperen, D. Van Den Berg, A. M. W. van den Ouweland, L. van der Kolk, R. B. van der Luijt, D. Vincent, J. Vollenweider, Q. Waisfisz, S. Wang-Gohrke, C. R. Weinberg, C. Wendt, A. S. Whittemore, H. Wildiers, W. Willett, R. Winqvist, A. Wolk, A. H. Wu, L. Xia, T. Yamaji, X. R. Yang, C. H. Yip, K.-Y. Yoo, J.-C. Yu, W. Zheng, Y. Zheng, B. Zhu, A. Ziogas, E. Ziv, ABCTB Investigators, ConFab/AOCS Investigators, S. R. Lakhani, A. C. Antoniou, A. Droit, I. L. Andrulis, C. I. Amos, F. J. Couch, P. D. P. Pharoah, J. Chang-Claude, P. Hall, D. J. Hunter, R. L. Milne, M. García-Closas, M. K. Schmidt, S. J. Chanock, A. M. Dunning, S. L. Edwards, G. D. Bader, G. Chenevix-Trench, J. Simard, P. Kraft, D. F.

Easton, Association analysis identifies 65 new breast cancer risk loci. *Nature* **551**, 92–94 (2017).

29. H. Zhang, T. U. Ahearn, J. Lecarpentier, D. Barnes, J. Beesley, G. Qi, X. Jiang, T. A. O'Mara, N. Zhao, M. K. Bolla, A. M. Dunning, J. Dennis, Q. Wang, Z. A. Ful, K. Aittomäki, I. L. Andrulis, H. Anton-Culver, V. Arndt, K. J. Aronson, B. K. Arun, P. L. Auer, J. Azzollini, D. Barrowdale, H. Becher, M. W. Beckmann, S. Behrens, J. Benitez, M. Bermisheva, K. Bialkowska, A. Blanco, C. Blomqvist, N. V. Bogdanova, S. E. Bojesen, B. Bonanni, D. Bondavalli, A. Borg, H. Brauch, H. Brenner, I. Briceno, A. Broeks, S. Y. Brucker, T. Brüning, B. Burwinkel, S. S. Buys, H. Byers, T. Caldés, M. A. Caligo, M. Calvello, D. Campa, J. E. Castelao, J. Chang-Claude, S. J. Chanock, M. Christiaens, H. Christiansen, W. K. Chung, K. B. M. Claes, C. L. Clarke, S. Cornelissen, F. J. Couch, A. Cox, S. S. Cross, K. Czene, M. B. Daly, P. Devilee, O. Diez, S. M. Domchek, T. Dörk, M. Dwek, D. M. Eccles, A. B. Ekici, D. G. Evans, P. A. Fasching, J. Figueroa, L. Foretova, F. Fostira, E. Friedman, D. Frost, M. Gago-Dominguez, S. M. Gapstur, J. Garber, J. A. García-Sáenz, M. M. Gaudet, S. A. Gayther, G. G. Giles, A. K. Godwin, M. S. Goldberg, D. E. Goldgar, A. González-Neira, M. H. Greene, J. Gronwald, P. Guénel, L. Häberle, E. Hahnen, C. A. Haiman, C. R. Hake, P. Hall, U. Hamann, E. F. Harkness, B. A. M. Heemskerk-Gerritsen, P. Hillemanns, F. B. L. Hogervorst, B. Holleczek, A. Hollestelle, M. J. Hooning, R. N. Hoover, J. L. Hopper, A. Howell, H. Huebner, P. J. Hulick, E. N. Imyanitov, kConFab Investigators, ABCTB Investigators, C. Isaacs, L. Izatt, A. Jager, M. Jakimovska, A. Jakubowska, P. James, R. Janavicius, W. Janni, E. M. John, M. E. Jones, A. Jung, R. Kaaks, P. M. Kapoor, B. Y. Karlan, R. Keeman, S. Khan, E. Khusnutdinova, C. M. Kitahara, Y.-D. Ko, I. Konstantopoulou, L. B. Koppert, S. Koutros, V. N. Kristensen, A.-V. Laenkholm, D. Lambrechts, S. C. Larsson, P. Laurent-Puig, C. Lazaro, E. Lazarova, F. Lejbkowicz, G. Leslie, F. Lesueur, A. Lindblom, J. Lissowska, W.-Y. Lo, J. T. Loud, J. Lubinski, A. Lukomska, R. J. MacInnis, A. Mannermaa, M. Manoochehri, S. Manoukian, S. Margolin, M. E. Martinez, L. Matricardi, L. M. Guffog, C. M. Lean, N. Mebirouk, A. Meindl, U. Menon, A. Miller, E. Mingazheva, M. Montagna, A. M. Mulligan, C. Mulot, T. A. Muranen, K. L. Nathanson, S. L. Neuhausen, H. Nevanlinna, P. Neven, W. G. Newman, F. C. Nielsen, L. Nikitina-Zake, J. Nodora, K. Offit, E. Olah, O. I. Olopade, H. Olsson, N. Orr, L. Papi, J. Papp, T.-W. Park-Simon, M. T. Parsons, B. Peissel, A. Peixoto, B. Peshkin, P. Peterlongo, J. Peto, K.-A. Phillips, M. Piedmonte, D. Plaseska-Karanfilska, K. Prajzendanc, R. Prentice, D. Prokofyeva, B. Rack, P. Radice, S. J. Ramus, J.

Rantala, M. U. Rashid, G. Rennert, H. S. Rennert, H. A. Risch, A. Romero, M. A. Rookus, M. Rübner, T. Rüdiger, E. Saloustros, S. Sampson, D. P. Sandler, E. J. Sawyer, M. T. Scheuner, R. K. Schmutzler, A. Schneeweiss, M. J. Schoemaker, B. Schöttker, P. Schürmann, L. Senter, P. Sharma, M. E. Sherman, X.-O. Shu, C. F. Singer, S. Smichkoska, P. Soucy, M. C. Southey, J. J. Spinelli, J. Stone, D. Stoppa-Lyonnet, EMBRACE Study, GEMO Study Collaborators, A. J. Swerdlow, C. I. Szabo, R. M. Tamimi, W. J. Tapper, J. A. Taylor, M. R. Teixeira, M. B. Terry, M. Thomassen, D. L. Thull, M. Tischkowitz, A. E. Toland, R. A. E. M. Tollenaar, I. Tomlinson, D. Torres, M. A. Troester, T. Truong, N. Tung, M. Untch, C. M. Vachon, A. M. W. van den Ouweland, L. E. van der Kolk, E. M. van Veen, E. J. vanRensburg, A. Vega, B. Wappenschmidt, C. R. Weinberg, J. N. Weitzel, H. Wildiers, R. Winqvist, A. Wolk, X. R. Yang, D. Yannoukakos, W. Zheng, K. K. Zorn, R. L. Milne, P. Kraft, J. Simard, P. D. P. Pharoah, K. Michailidou, A. C. Antoniou, M. K. Schmidt, G. Chenevix-Trench, D. F. Easton, N. Chatterjee, M. García-Closas, Genome-wide association study identifies 32 novel breast cancer susceptibility loci from overall and subtype-specific analyses. *Nat. Genet.* **52**, 572–581 (2020).

30. V. P. Nimbalkar, S. Rajarajan, S. V. P, A. Alexander, R. Kaluve, S. Selvam, R. Ramesh, S. B. S, J. S. Prabhu, A comparative analysis of clinicopathological features and survival between pre and postmenopausal breast cancer from an Indian cohort. *Sci. Rep.* **13**, 3938 (2023).

31. L. Chollet-Hinton, C. K. Anders, C.-K. Tse, M. B. Bell, Y. C. Yang, L. A. Carey, A. F. Olshan, M. A. Troester, Breast cancer biologic and etiologic heterogeneity by young age and menopausal status in the Carolina Breast Cancer Study: A case-control study. *Breast Cancer Res.* **18**, 79 (2016).

32. T. H. M. Keegan, M. C. DeRouen, D. J. Press, A. W. Kurian, C. A. Clarke, Occurrence of breast cancer subtypes in adolescent and young adult women. *Breast Cancer Res.* **14**, R55 (2012).

33. J. Zheng, A. M. Erzurumluoglu, B. L. Elsworth, J. P. Kemp, L. Howe, P. C. Haycock, G. Hemani, K. Tansey, C. Laurin, Early Genetics and Lifecourse Epidemiology (EAGLE) Eczema Consortium, B. S. Pourcain, N. M. Warrington, H. K. Finucane, A. L. Price, B. K. Bulik-Sullivan, V. Anttila, L. Paternoster, T. R. Gaunt, D. M. Evans, B. M. Neale, LD Hub: A

centralized database and web interface to perform LD score regression that maximizes the potential of summary level GWAS data for SNP heritability and genetic correlation analysis. *Bioinformatics* **33**, 272–279 (2017).

34. M. R. Munafò, K. Tilling, A. E. Taylor, D. M. Evans, G. Davey, Collider scope: When selection bias can substantially influence observed associations. *Int. J. Epidemiol.* **47**, 226–235 (2018).

35. M. Vabistsevits, G. Davey Smith, T. G. Richardson, R. C. Richmond, W. Sieh, J. H. Rothstein, L. A. Habel, S. E. Alexeeff, B. Lloyd-Lewis, E. Sanderson, Mammographic density mediates the protective effect of early-life body size on breast cancer risk. *Nat. Commun.* **15**, 4021 (2024).

36. K. A. Kentistou, J. Sundfjord, R. Karimi, L. R. Kaisinger, R. J. Hofmeister, A. E. Lupu, N. Fragoso-Bargas, Y. Zhao, J. A. Tadross, L. Steuernagel, G. K. C. Dowsett, S. Lockhart, J. C. Bruening, J. Liu, A. Cortes, Y. Lo, J. Davitte, L. Clement, A. Havdahl, O. A. Andreassen, E. Bratland, B. Y. H. Lam, S. O'Rahilly, G. S. H. Yeo, P. R. Njølstad, Z. Kutalik, F. R. Day, M. Vaudel, J. R. B. Perry, K. K. Ong, S. Johansson, The role of common and rare genetic variation on adiposity across childhood. medRxiv 25327505 (2025). https://doi.org/10.1101/2025.05.13.25327505.

37. K. A. Kentistou, L. R. Kaisinger, S. Stankovic, M. Vaudel, E. M. de Oliveira, A. Messina, R. G. Walters, X. Liu, A. S. Busch, H. Helgason, D. J. Thompson, F. Santoni, K. M. Petricek, Y. Zouaghi, I. Huang-Doran, D. F. Gudbjartsson, E. Bratland, K. Lin, E. J. Gardner, Y. Zhao, R. Y. Jia, C. Terao, M. J. Riggan, M. K. Bolla, M. Yazdanpanah, N. Yazdanpanah, J. P. Bradfield, L. Broer, A. Campbell, D. I. Chasman, D. L. Cousminer, N. Franceschini, L. H. Franke, G. Girotto, C. He, M.-R. Järvelin, P. K. Joshi, Y. Kamatani, R. Karlsson, J. Luan, K. L. Lunetta, R. Mägi, M. Mangino, S. E. Medland, C. Meisinger, R. Noordam, T. Nutile, M. P. Concas, O. Polašek, E. Porcu, S. M. Ring, C. Sala, A. V. Smith, T. Tanaka, P. J. van der Most, V. Vitart, C. A. Wang, G. Willemsen, M. Zygmunt, T. U. Ahearn, I. L. Andrulis, H. Anton-Culver, A. C. Antoniou, P. L. Auer, C. L. K. Barnes, M. W. Beckmann, A. B. de Gonzalez, N. V. Bogdanova, S. E. Bojesen, H. Brenner, J. E. Buring, F. Canzian, J. Chang-Claude, F. J. Couch, A. Cox, L. Crisponi, K. Czene, M. B. Daly, E. W. Demerath, J. Dennis, P. Devilee, I.

De Vivo, T. Dörk, A. M. Dunning, M. Dwek, J. G. Eriksson, P. A. Fasching, L. Fernandez-Rhodes, L. Ferreli, O. Fletcher, M. Gago-Dominguez, M. García-Closas, J. A. García-Sáenz, A. González-Neira, H. Grallert, P. Guénel, C. A. Haiman, P. Hall, U. Hamann, H. Hakonarson, R. J. Hart, M. Hickey, M. J. Hooning, R. Hoppe, J. L. Hopper, J.-J. Hottenga, F. B. Hu, H. Huebner, D. J. Hunter, ABCTB Investigators, H. Jernström, E. M. John, D. Karasik, E. K. Khusnutdinova, V. N. Kristensen, J. V. Lacey, D. Lambrechts, L. J. Launer, P. A. Lind, A. Lindblom, P. K. E. Magnusson, A. Mannermaa, M. I. McCarthy, T. Meitinger, C. Menni, K. Michailidou, I. Y. Millwood, R. L. Milne, G. W. Montgomery, H. Nevanlinna, I. M. Nolte, D. R. Nyholt, N. Obi, K. M. O'Brien, K. Offit, A. J. Oldehinkel, S. R. Ostrowski, A. Palotie, O. B. Pedersen, A. Peters, G. Pianigiani, D. Plaseska-Karanfilska, A. Pouta, A. Pozarickij, P. Radice, G. Rennert, F. R. Rosendaal, D. Ruggiero, E. Saloustros, D. P. Sandler, S. Schipf, C. O. Schmidt, M. K. Schmidt, K. Small, B. Spedicati, M. Stampfer, J. Stone, R. M. Tamimi, L. R. Teras, E. Tikkanen, C. Turman, C. M. Vachon, Q. Wang, R. Winqvist, A. Wolk, B. S. Zemel, W. Zheng, K. W. van Dijk, B. Z. Alizadeh, S. Bandinelli, E. Boerwinkle, D. I. Boomsma, M. Ciullo, G. Chenevix-Trench, F. Cucca, T. Esko, C. Gieger, S. F. A. Grant, V. Gudnason, C. Hayward, I. Kolčić, P. Kraft, D. A. Lawlor, N. G. Martin, E. A. Nøhr, N. L. Pedersen, C. E. Pennell, P. M. Ridker, A. Robino, H. Snieder, U. Sovio, T. D. Spector, D. Stöckl, C. Sudlow, N. J. Timpson, D. Toniolo, A. Uitterlinden, S. Ulivi, H. Völzke, N. J. Wareham, E. Widen, J. F. Wilson, Lifelines Cohort Study, Danish Blood Donor Study, Ovarian Cancer Association Consortium, Breast Cancer Association Consortium, Biobank Japan Project, China Kadoorie Biobank Collaborative Group, P. D. P. Pharoah, L. Li, D. F. Easton, P. R. Njølstad, P. Sulem, J. M. Murabito, A. Murray, D. Manousaki, A. Juul, C. Erikstrup, K. Stefansson, M. Horikoshi, Z. Chen, I. S. Farooqi, N. Pitteloud, S. Johansson, F. R. Day, J. R. B. Perry, K. K. Ong, Understanding the genetic complexity of puberty timing across the allele frequency spectrum. *Nat. Genet.* **56**, 1397–1411 (2024).

38. K. Silventoinen, A. Jelenkovic, T. Palviainen, L. Dunkel, J. Kaprio, The association between puberty timing and body mass index in a longitudinal setting: The contribution of genetic factors. *Behav. Genet.* **52**, 186–194 (2022).

39. R. Lloyd, S. Pirikahu, J. Walter, G. Cadby, N. Warrington, D. Perera, M. Hickey, C. Saunders, M. Hackmann, D. D. Sampson, J. Shepherd, L. Lilge, J. Stone, The prospective association between early life growth and breast density in young adult women. *Cancer* **16**, 2418 (2024).

40. T. X. M. Tran, Y. Chang, H. R. Choi, R. Kwon, G.-Y. Lim, E. Y. Kim, S. Ryu, B. Park, Adiposity, body composition measures, and breast cancer risk in Korean premenopausal women. *JAMA Netw. Open* **7**, e245423 (2024).

41. P. A. van den Brandt, D. Spiegelman, S. S. Yaun, H. O. Adami, L. Beeson, A. R. Folsom, G. Fraser, R. A. Goldbohm, S. Graham, L. Kushi, J. R. Marshall, A. B. Miller, T. Rohan, S. A. Smith-Warner, F. E. Speizer, W. C. Willett, A. Wolk, D. J. Hunter, Pooled analysis of prospective cohort studies on height, weight, and breast cancer risk. *Am. J. Epidemiol.* **152**, 514–527 (2000).

42. P. A. van den Brandt, R. G. Ziegler, M. Wang, T. Hou, R. Li, H. O. Adami, C. Agnoli, L. Bernstein, J. E. Buring, Y. Chen, A. E. Connor, A. H. Eliassen, J. M. Genkinger, G. Gierach, G. G. Giles, G. G. Goodman, N. Håkansson, V. Krogh, L. Le Marchand, I. M. Lee, L. M. Liao, M. E. Martinez, A. B. Miller, R. L. Milne, M. L. Neuhouser, A. V. Patel, A. Prizment, K. Robien, T. E. Rohan, N. Sawada, L. J. Schouten, R. Sinha, R. Z. Stolzenberg-Solomon, L. R. Teras, S. Tsugane, K. Visvanathan, E. Weiderpass, K. K. White, W. C. Willett, A. Wolk, A. Zeleniuch-Jacquotte, S. A. Smith-Warner, Body size and weight change over adulthood and risk of breast cancer by menopausal and hormone receptor status: A pooled analysis of 20 prospective cohort studies. *Eur. J. Epidemiol.* **36**, 37–55 (2021).

43. T. Dehesh, S. Fadaghi, M. Seyedi, E. Abolhadi, M. Ilaghi, P. Shams, F. Ajam, M. A. Mosleh-Shirazi, P. Dehesh, The relation between obesity and breast cancer risk in women by considering menstruation status and geographical variations: A systematic review and meta-analysis. *BMC Womens Health* **23**, 392 (2023).

44. C. M. Friedenreich, Review of anthropometric factors and breast cancer risk. *Eur. J. Cancer Prev.* **10**, 15–32 (2001).

45. M. L. Neuhouser, A. K. Aragaki, R. L. Prentice, J. E. Manson, R. Chlebowski, C. L. Carty, H. M. Ochs-Balcom, C. A. Thomson, B. J. Caan, L. F. Tinker, R. P. Urrutia, J. Knudtson, G. L. Anderson, Overweight, obesity, and postmenopausal invasive breast cancer risk: A secondary analysis of the women's health initiative randomized clinical trials. *JAMA Oncol.* **1**, 611–621 (2015).

46. I. Glassman, N. Le, A. Asif, A. Goulding, C. A. Alcantara, A. Vu, A. Chorbajian, M. Mirhosseini, M. Singh, V. Venketaraman, The role of obesity in breast cancer pathogenesis. *Cells* **12**, 2061 (2023).

47. R. S. Cecchini, J. P. Costantino, J. A. Cauley, W. M. Cronin, D. L. Wickerham, S. R. Land, J. L. Weissfeld, N. Wolmark, Body mass index and the risk for developing invasive breast cancer among high-risk women in NSABP P-1 and STAR breast cancer prevention trials. *Cancer Prev. Res.* **5**, 583–592 (2012).

48. P. Zhao, N. Xia, H. Zhang, T. Deng, The metabolic syndrome is a risk factor for breast cancer: A systematic review and meta-analysis. *Obes. Facts* **13**, 384–396 (2020).

49. F. Ligorio, L. Zambelli, G. Fucà, R. Lobefaro, M. Santamaria, E. Zattarin, F. de Braud, C. Vernieri, Prognostic impact of body mass index (BMI) in HER2+ breast cancer treated with anti-HER2 therapies: From preclinical rationale to clinical implications. *Ther. Adv. Med. Oncol.* **14**, 17588359221079123 (2022).

50. B. O. Åsvold, A. Langhammer, T. A. Rehn, G. Kjelvik, T. V. Grøntvedt, E. P. Sørgjerd, J. S. Fenstad, J. Heggland, O. Holmen, M. C. Stuifbergen, S. A. A. Vikjord, B. M. Brumpton, H. K. Skjellegrind, P. Thingstad, E. R. Sund, G. Selbæk, P. J. Mork, V. Rangul, K. Hveem, M. Næss, S. Krokstad, Cohort profile update: The HUNT study, Norway. *Int. J. Epidemiol.* **52**, e80–e91 (2022).

51. P. Magnus, C. Birke, K. Vejrup, A. Haugan, E. Alsaker, A. K. Daltveit, M. Handal, M. Haugen, G. Høiseth, G. P. Knudsen, L. Paltiel, P. Schreuder, K. Tambs, L. Vold, C. Stoltenberg, Cohort profile update: The Norwegian Mother and Child Cohort Study (MoBa). *Int. J. Epidemiol.* **45**, 382–388 (2016).

52. A. Fraser, C. Macdonald-Wallis, K. Tilling, A. Boyd, J. Golding, G. Davey, J. Henderson, J. Macleod, L. Molloy, A. Ness, S. Ring, S. M. Nelson, D. A. Lawlor, Cohort profile: The avon longitudinal study of parents and children: ALSPAC mothers cohort. *Int. J. Epidemiol.* **42**, 97–110 (2013).

53. A. Boyd, J. Golding, J. Macleod, D. A. Lawlor, A. Fraser, J. Henderson, L. Molloy, A. Ness, S. Ring, G. Davey Smith, Cohort profile: The 'children of the 90s'—The index offspring of the Avon Longitudinal Study of Parents and Children. *Int. J. Epidemiol.* **42**, 111–127 (2013).

54. K. Northstone, M. Lewcock, A. Groom, A. Boyd, J. Macleod, N. Timpson, N. Wells, The Avon Longitudinal Study of Parents and Children (ALSPAC): An update on the enrolled sample of index children in 2019. *Wellcome Open Res.* **4**, 51 (2019).

55. P. A. Harris, R. Taylor, R. Thielke, J. Payne, N. Gonzalez, J. G. Conde, Research electronic data capture (REDCap)—A metadata-driven methodology and workflow process for providing translational research informatics support. *J. Biomed. Inform.* **42**, 377–381 (2009).

56. S. Krokstad, A. Langhammer, K. Hveem, T. L. Holmen, K. Midthjell, T. R. Stene, G. Bratberg, J. Heggland, J. Holmen, Cohort profile: The HUNT Study, Norway. *Int. J. Epidemiol.* **42**, 968–977 (2013).

57. L. M. Irgens, The Medical Birth Registry of Norway. Epidemiological research and surveillance throughout 30 years. *Acta Obstet. Gynecol. Scand.* **79**, 435–439 (2000).

58. G.-H. Moen, B. Brumpton, C. Willer, B. O. Åsvold, K. I. Birkeland, G. Wang, M. C. Neale, R. M. Freathy, G. Davey Smith, D. A. Lawlor, R. M. Kirkpatrick, N. M. Warrington, D. M. Evans, Mendelian randomization study of maternal influences on birthweight and future cardiometabolic risk in the HUNT cohort. *Nat. Commun.* **11**, 5404 (2020).

59. B. M. Brumpton, S. Graham, I. Surakka, A. H. Skogholt, M. Løset, L. G. Fritsche, B. Wolford, W. Zhou, J. B. Nielsen, O. L. Holmen, M. E. Gabrielsen, L. Thomas, L. Bhatta, H. Rasheed, H. Zhang, H. M. Kang, W. Hornsby, M. R. Moksnes, E. Coward, M. Melbye, G. F. Giskeødegård, J. Fenstad, S. Krokstad, M. Næss, A. Langhammer, M. Boehnke, G. R. Abecasis, B. O. Åsvold, K. Hveem, C. J. Willer, The HUNT study: A population-based cohort for genetic research. *Cell Genom.* **2**, 100193 (2022).

60. L. Paltiel, H. Anita, T. Skjerden, K. Harbak, S. Bækken, S. N. Kristin, G. P. Knudsen, P. Magnus, The biobank of the Norwegian Mother and Child Cohort Study – present status. *Norsk Epidemiologi* **24**, 10.5324/nje.v24i1-2.1755 (2014).

61. M. N. Kooijman, C. J. Kruithof, C. M. van Duijn, L. Duijts, O. H. Franco, M. H. van IJzendoorn, J. C. de Jongste, C. C. Klaver, A. van der Lugt, J. P. Mackenbach, H. A. Moll, R. P. Peeters, H. Raat, E. H. Rings, F. Rivadeneira, M. P. van der Schroeff, E. A. Steegers, H. Tiemeier, A. G. Uitterlinden, F. C. Verhulst, E. Wolvius, J. F. Felix, V. W. V. Jaddoe, The Generation R Study: Design and cohort update 2017. *Eur. J. Epidemiol.* **31**, 1243–1264 (2016).

62. S. Ghatan, J. de Vries, J.-B. Pingault, V. W. Jaddoe, C. Cecil, J. F. Felix, F. Rivadeneira, C. Medina-Gomez, Genetic nurture: Estimating the direct genetic effects of pediatric anthropometric traits. medRxiv 24318796 [Preprint] (2024). https://doi.org/10.1101/2024.12.10.24318796.

63. M. Næss, K. Kvaløy, E. P. Sørgjerd, K. S. Sætermo, L. Norøy, A. H. Røstad, N. Hammer, T. G. Altø, A. J. Vikdal, K. Hveem, Data resource profile: The HUNT Biobank. *Int. J. Epidemiol.* **53**, dyae073 (2024).

64. E. C. Corfield, A. A. Shadrin, O. Frei, Z. Rahman, A. Lin, L. Athanasiu, B. C. Akdeniz, T. T. Filiz, L. Hannigan, R. E. Wootton, C. Austerberry, A. Hughes, M. Tesli, L. T. Westlye, H. Stefánsson, K. Stefánsson, P. R. Njølstad, P. Magnus, N. M. Davies, V. Appadurai, G. Hemani, E. Hovig, T. Zayats, H. Ask, T. Reichborn-Kjennerud, O. A. Andreassen, A. Havdahl, The Norwegian Mother, Father, and Child cohort study (MoBa) genotyping data resource: MoBaPsychGen pipeline v.1. bioRxiv 496289 [Preprint] (2024). https://doi.org/10.1101/2022.06.23.496289.

65. J. Yang, S. H. Lee, M. E. Goddard, P. M. Visscher, GCTA: A tool for genome-wide complex trait analysis. *Am. J. Hum. Genet.* **88**, 76–82 (2011).

66. L. Jiang, Z. Zheng, T. Qi, K. E. Kemper, N. R. Wray, P. M. Visscher, J. Yang, A resource-efficient tool for mixed model association analysis of large-scale data. *Nat. Genet.* **51**, 1749–1755 (2019).

67. C. J. Willer, Y. Li, G. R. Abecasis, METAL: Fast and efficient meta-analysis of genomewide association scans. *Bioinformatics* **26**, 2190–2191 (2010).

68. A. Ani, P. J. van der Most, H. Snieder, A. Vaez, I. M. Nolte, GWASinspector: Comprehensive quality control of genome-wide association study results. *Bioinformatics* **37**, 129–130 (2021).

69. B. K. Bulik-Sullivan, P.-R. Loh, H. K. Finucane, S. Ripke, J. Yang, Schizophrenia Working Group of the Psychiatric Genomics Consortium, N. Patterson, M. J. Daly, A. L. Price, B. M. Neale, LD Score regression distinguishes confounding from polygenicity in genome-wide association studies. *Nat. Genet.* **47**, 291–295 (2015).

70. S. S. Paria, S. R. Rahman, K. Adhikari, fastman: A fast algorithm for visualizing GWAS results using Manhattan and Q-Q plots. bioRxiv 488738 [Preprint] (2022). https://doi.org/10.1101/2022.04.19.488738.

71. C. C. Chang, C. C. Chow, L. C. Tellier, S. Vattikuti, S. M. Purcell, J. J. Lee, Second-generation PLINK: Rising to the challenge of larger and richer datasets. *Gigascience* **4**, 7 (2015).

72.1000G enomes Project Consortium, G. R. Abecasis, A. Auton, L. D. Brooks, M. A. DePristo, R. M. Durbin, R. E. Handsaker, H. M. Kang, G. T. Marth, G. A. McVean, An integrated map of genetic variation from 1,092 human genomes. *Nature* **491**, 56–65 (2012).

73. C. Sudlow, J. Gallacher, N. Allen, V. Beral, P. Burton, J. Danesh, P. Downey, P. Elliott, J. Green, M. Landray, B. Liu, P. Matthews, G. Ong, J. Pell, A. Silman, A. Young, T. Sprosen, T. Peakman, R. Collins, UK biobank: An open access resource for identifying the causes of a wide range of complex diseases of middle and old age. *PLOS Med.* **12**, e1001779 (2015).

74. C. Bycroft, C. Freeman, D. Petkova, G. Band, L. T. Elliott, K. Sharp, A. Motyer, D. Vukcevic, O. Delaneau, J. O'Connell, A. Cortes, S. Welsh, A. Young, M. Effingham, G. McVean, S. Leslie, N. Allen, P. Donnelly, J. Marchini, The UK Biobank resource with deep phenotyping and genomic data. *Nature* **562**, 203–209 (2018).

75. M. Brandkvist, J. H. Bjørngaard, R. A. Ødegård, B. O. Åsvold, G. Davey Smith, B. Brumpton, K. Hveem, T. G. Richardson, G. Å. Vie, Separating the genetics of childhood and

adult obesity: A validation study of genetic scores for body mass index in adolescence and adulthood in the HUNT Study. *Hum. Mol. Genet.* **29**, 3966–3973 (2021).

76. T. G. Richardson, J. Mykkänen, K. Pahkala, M. Ala-Korpela, J. A. Bell, K. Taylor, J. Viikari, T. Lehtimäki, O. Raitakari, G. Davey Smith, Evaluating the direct effects of childhood adiposity on adult systemic metabolism: A multivariable Mendelian randomization analysis. *Int. J. Epidemiol.* **50**, 1580–1592 (2021).

77. G. Hemani, J. Zheng, B. Elsworth, K. H. Wade, V. Haberland, D. Baird, C. Laurin, S. Burgess, J. Bowden, R. Langdon, V. Y. Tan, J. Yarmolinsky, H. A. Shihab, N. J. Timpson, D. M. Evans, C. Relton, R. M. Martin, G. Davey Smith, T. R. Gaunt, P. C. Haycock, The MR-Base platform supports systematic causal inference across the human phenome. *eLife* **7**, e34408 (2018).

78. R. C. Richmond, G. Davey Smith, Mendelian randomization: Concepts and scope. *Cold Spring Harb. Perspect. Med.* **12**, a040501 (2022).

79. J. Bowden, G. Davey Smith, S. Burgess, Mendelian randomization with invalid instruments: Effect estimation and bias detection through Egger regression. *Int. J. Epidemiol.* **44**, 512–525 (2015).

80. J. Bowden, G. Davey Smith, P. C. Haycock, S. Burgess, Consistent estimation in Mendelian randomization with some invalid instruments using a weighted median estimator. *Genet. Epidemiol.* **40**, 304–314 (2016).

81. G. Hemani, K. Tilling, G. Davey Smith, Orienting the causal relationship between imprecisely measured traits using GWAS summary data. *PLOS Genet.* **13**, e1007081 (2017).

82. Q. Zhao, J. Wang, G. Hemani, J. Bowden, D. S. Small, Statistical inference in two-sample summary-data Mendelian randomization using robust adjusted profile score. *Ann. Statist.* **48**, 1742–1769 (2020).

83. E. Sanderson, W. Spiller, J. Bowden, Testing and correcting for weak and pleiotropic instruments in two-sample multivariable Mendelian randomization. *Stat. Med.* **40**, 5434–5452 (2021).

84. B. Woolf, S. Burgess, The role of estimation in Mendelian randomization: Should Mendelian randomization investigations provide estimates? *AJE Adv.* **1**, uuaf003 (2025).

85. Collaborative Group on Hormonal Factors in Breast Cancer, Menarche, menopause, and breast cancer risk: Individual participant meta-analysis, including 118 964 women with breast cancer from 117 epidemiological studies. *Lancet Oncol.* **13**, 1141–1151 (2012).

86. G. V. Dall, K. L. Britt, Estrogen effects on the mammary gland in early and late life and breast cancer risk. *Front. Oncol.* **7**, 110 (2017).

87. F. Juul, V. W. Chang, P. Brar, N. Parekh, Birth weight, early life weight gain and age at menarche: A systematic review of longitudinal studies. *Obes. Rev.* **18**, 1272–1288 (2017).

88. C. Prince, L. D. Howe, G. C. Sharp, A. Fraser, R. C. Richmond, Establishing the relationships between adiposity and reproductive factors: A multivariable Mendelian randomization analysis. *BMC Med.* **21**, 350 (2023).

89. R. C. Team, R: A language and environment for statistical computing (2020). R Foundation for Statistical Computing, Vienna, Austria. https://www.r-project.org/.

90. R. Wootton, A. Havdahl, Combining Mendelian Randomisation and Depression Trajectories to Better Inform Intervention Timing, Open Science. Framework (2025); https://osf.io/nu3zr/.

91. The Haplotype Reference Consortium, A reference panel of 64,976 haplotypes for genotype imputation. *Nat. Genet.* **48**, 1279–1283 (2016).

92. The 1000 Genomes Project Consortium, A global reference for human genetic variation. *Nature* **526**, 68–74 (2015).

93. ENCODE Project Consortium, An integrated encyclopedia of DNA elements in the human genome. *Nature* **489**, 57–74 (2012).

94. C. Wang, X. Zhan, J. Bragg-Gresham, H. M. Kang, D. Stambolian, E. Y. Chew, K. E. Branham, J. Heckenlively, FUSION Study, R. Fulton, R. K. Wilson, E. R. Mardis, X. Lin, A. Swaroop, S. Zöllner, G. R. Abecasis, Ancestry estimation and control of population stratification for sequence-based association studies. *Nat. Genet.* **46**, 409–415 (2014).

95. J. Z. Li, D. M. Absher, H. Tang, A. M. Southwick, A. M. Casto, S. Ramachandran, H. M. Cann, G. S. Barsh, M. Feldman, L. L. Cavalli-Sforza, R. M. Myers, Worldwide human relationships inferred from genome-wide patterns of variation. *Science* **319**, 1100–1104 (2008).

96. P. R. Loh, P. Danecek, P. F. Palamara, C. Fuchsberger, Y. A. Reshef, H. K. Finucane, S. Schoenherr, L. Forer, S. M. Carthy, G. R. Abecasis, R. Durbin, A. L. Price, Reference-based phasing using the Haplotype Reference Consortium panel. *Nat. Genet.* **48**, 1443–1448 (2016).

97. S. Das, L. Forer, S. Schönherr, C. Sidore, A. E. Locke, A. Kwong, S. I. Vrieze, E. Y. Chew, S. Levy, M. McGue, D. Schlessinger, D. Stambolian, P. R. Loh, W. G. Iacono, A. Swaroop, L. J. Scott, F. Cucca, F. Kronenberg, M. Boehnke, G. R. Abecasis, C. Fuchsberger, Next-generation genotype imputation service and methods. *Nat. Genet.* **48**, 1284–1287 (2016).

98. V. W. Skrivankova, R. C. Richmond, B. A. R. Woolf, J. Yarmolinsky, N. M. Davies, S. A. Swanson, T. J. VanderWeele, J. P. T. Higgins, N. J. Timpson, N. Dimou, C. Langenberg, R. M. Golub, E. W. Loder, V. Gallo, A. Tybjaerg-Hansen, G. Davey, M. Egger, J. B. Richards, Strengthening the reporting of observational studies in epidemiology using mendelian randomization. *JAMA* **326**, 1614–1621 (2021).

99. V. W. Skrivankova, R. C. Richmond, B. A. R. Woolf, N. M. Davies, S. A. Swanson, T. J. VanderWeele, N. J. Timpson, J. P. T. Higgins, N. Dimou, C. Langenberg, E. W. Loder, R. M. Golub, M. Egger, G. Davey Smith, J. B. Richards, Strengthening the reporting of observational studies in epidemiology using mendelian randomisation (STROBE-MR): Explanation and elaboration. *BMJ* **375**, n2233 (2021).
